# Discrete optimal quadratic AGC based cost functional minimization for interconnected power systems

**DOI:** 10.1038/s41598-023-29317-1

**Published:** 2023-02-16

**Authors:** M. Esmail, S. Krishnamurthy

**Affiliations:** Department of Electrical, Electronic and Computer Engineering, Cape Peninsula University of Technology, Symphony Way, Bellville, Cape Town, 7535 South Africa

**Keywords:** Energy science and technology, Engineering, Mathematics and computing

## Abstract

The increasing complexity and difficulty of the Automatic generation control (AGC) problem has resulted from the increasing scale of interconnected power networks and changing daily demands. The primary goals of AGC are to control frequency variations at nominal levels and tie-line power variances at planned levels. To effectively deal with AGC control difficulties, this study introduces Discrete Optimal Quadratic Automatic Generation Control (OQAGC). One advantages of this method is the differentiation of quadratic cost function results into linear terms while minimizing control actions and minimizing state deviations. This developed control method leads to a simple and easy discrete control law that can be implemented for both linear and nonlinear systems. For optimizing the controller, this research work utilized an optimum control theorem using Lagrangian multipliers, while the functional minimization technique is used for systematically selecting the state and control weighting matrices in discrete form for N control regions (where N is the number of interconnected power systems). The discrete cost function needs are derived using this technique in terms of area control errors, integral area control errors, and control energy expenditure. Four interconnected power systems were analyzed with/without disturbances and area control errors, each with one thermal, hydro, and gas-generating unit. A two-area multi-source power system with renewable energy in control area 2 is analyzed for the performance of the proposed controller with generation rate constraints (GRCs). The functional minimization technique simplifies and eases the choosing of weighting matrices. Furthermore, the simulation findings suggest that the developed discrete optimum quadratic AGC control-based cost functional minimization approach enhances power system dynamics in terms of stability, steady-state performance, and the closed-loop control system's robustness to input load disturbances. As a result, the newly developed OQAGC approach demonstrates the significance of the discrete LQR controller for N multi-area power systems.

## Introduction

The control of active power is a major requirement in the day-to-day management for any modern energy power systems^1^. The main objectives of this control are to maintain the frequency deviations at a nominal value, keep the tie-line power changes between areas at a scheduled value, and ensure that the frequency variations are returned to zero^2–4^. In other words, the power losses and loads are sensitive to generator’s speed and frequency. Therefore, for satisfactory operation, the mechanical power and the electric power delivered to consumers must be matched. The system frequency depends on the active power balance. Therefore, a mismatch in active power reflects a change in frequency. Once a load is added to the power system, the power mismatch is initially compensated by extracting kinetic energy from the system's inertial storage, resulting in a drop in power system frequency. A decrease in frequency leads to a decrease in power taken by loads. At equilibrium, the frequency will be constant or at nominal value^5,6^^.^. In contrast, distributed resources have totally different behavior comparing to the classical generators because they are interfaced via power electronic devices^7^. As result, there is no coupling between the rotational speed of the generator and the system frequency^8^, and hence, inverter-connecting generating units does not inherently contribute to the total system inertia^9^. Therefore, the distributed energy resources integrated into power systems act as additional disturbances to the power system under consideration. Due to this, the increasing of the load demands makes this control problem challenging. Besides the interconnected power system are growing in size due to the integration of the new distributed resources i.e. wind-farms and, PV, into the main grid, adopting new concepts i.e. smart grid and digitization of the power systems makes this control even more complex and challenging^10^.

1. *Background:* At the beginning, the bias tie-line control was introduced by Cohn in 1956 for an interconnected power system. The frequency of the power system was controlled via the governor of the synchronous machine using a flywheel mechanism^11^. This technique was later found to be insufficient. Therefore, the idea of supplementary control was added to the governor with the help of the Area control error signal (ACE)^12^. The most widely used supplementary control types in the literature are the classical linear controllers: integral control(I), proportional-integral (PI), and proportional integral Derivative (PID) controllers because of their simplicity in design and implementation. Hence, their major drawback was the degradation of the controlled system performance due to the nonlinearities of the system and the system operating points^13^. In addition, blackout may result, owing to the increased size and complexity of modern power systems as the oscillations will propagate into interconnected power systems^14^. To address these challenges, optimal quadratic AGC control method based on optimal modern control theories is required to regulate the frequency and tie-line power flow. The discrete quadratic regulator is used because in most modern control applications, the control law is built around a computer (microcontrollers), where the action of the control law is provided during the sampling period. In addition, the linear control law can be applied to both linear and nonlinear systems based on the assumption of linearity that justifies the use of optimal linear control theories. Finally, the differentiation outcome of quadratic cost function leads to a linear term and compromises between minimizing the control action and the state variables^15^.
2. *Literature review*: In the past various control categories have been adopted to deal with AGC problems. These control strategies were ranged from robust control methods, variable structure control, adaptive control schemes, robust control methods, digital control and intelligent techniques and amongst others. The thorough reviews conducted give the complete literature analysis of various AGC control mechanisms along with their benefits and disadvantages^10,12,16,17^. Several AGC studies have been conducted in the past decade to address AGC problems in complex interconnected power systems using soft computing and intelligent control techniques, including; two-degree-of-freedom-fractional order-PID (2-DOF-FOPID) Controller^18^, 2DOF PID controller based on FACTS and firefly optimization methods^19^, multiobjective based optimization of artificial bee colony (ABC) algorithm for load frequency control (LFC)^20^, differential evolution (DE) algorithm^21^, bacterial foraging optimization algorithm (BFOA)^22^, a quasi-oppositional harmony search (QOHS) algorithm based proportional–integral–derivative (PID)^23^, teaching learning based optimisation (TLBO)^24^, two degree of freedom -integral plus double derivative (2DOF-IDD ) based on cuckoo search algorithm (CS)^25^, power system stabilizer (PSS) and static synchronous series compensator (SSSC) coordinated controllers based on seeker optimization algorithm (SOA)^26^, and fuzzy gain scheduling controller based on genetic algorithm (GA)^27^. recently, a novel cascade fuzzy integral derivative-filter (FPIDN) –fractional order PIDN (FPIDN-FOPIDN) controller based on Imperialist Competitive Algorithm (ICA) has been proposed to effectively deal with AGC issues in various two-area control interconnected power systems^28^. This mainly due to their low-cost solution and guarantees to provide practical solutions, as well as their ability to handle uncertainties, nonlinearities, and complexity of the power systems^10^. Besides, Genetic algorithms (GA) based optimal controllers are found to be associated with high correlation of the optimized parameters and GA’s performance and its research capabilities are degraded and reduced due to premature convergence^29^. Further, particle swarm optimization (PSO) is associated with the phenomena of explosion of the swarm (particles diverged to infinity) even if the maximum speed and acceleration are correctly defined^23^. Despite the advantages of the soft computing based AGC control techniques, they give an approximated output values instead of true optimal values.
  A modern optimal control theory has also been investigated in the literature as one of the control strategies to deal with the complexity of interconnected power systems. The first optimal AGC control was introduced by Fosha and Elgerd^30^, and Elgerd and Fosha^31^ for two-area interconnected power systems. In these papers, the authors proposed an optimal AGC controlled using full state feedback control based on the law of a proportional-integral (PI) control law to minimize the cost function and determine a gain feedback matrix. The design of optimal control aims to determine the optimal control law that can transfer the system from its initial state to the final state such that a given cost function is minimized^32^. Ibraheem and Kumar^33^ proposed optimal AGC based on state feedback control and three different approaches for selecting weighting matrices to minimize the cost function and find the optimal gains of the feedback matrix for two area interconnected power system with non-reheat turbines. The three approaches are the controllability and observability indices of the system, functional minimization method (FMM), and engineering judgement based on the selection of all states of the power system. In the first approach, the state matrix of the power system is transformed to diagonal matrix form by using the Eigenvalues and Eigenvectors decomposition method. in the second approach, the weighting matrices are obtained by the minimum values of the functional of the cost function, which can be obtained through the partial differentials of the functional of the cost function. This approach considers few output variables, namely the minimum of area control error, minimum of the integral area control error and the minimum of the control signals. Finally, the third approach’s weighting matrices are selected by considering all state variables involved in the control action. Weighting matrices are generally constructed based on identity matrices based on power system order. Also, to consider the communication delays and ensure the stability of interconnected power system in real-time environment, the authors (Pathak et al.^34^) proposed optimal centralized PI control based on state feedback control and functional minimization method (FMM) for identical two area interconnected power system. To minimize the follow of the tie-line power and frequency deviations, the authors (Tungadio et al.^2^) designed an optimal controller based on fmincon to control the active power balance of two micro-grids linked by two AC tie-lines. fmincon is a built-in MATLAB function used to solve optimization problems. Each micro -grid consists of wind farm, hydro plant, battery energy storage system and the load demand. This control method can handle robustness, reliability and nonlinearities associated with the power system when compared to PID linear controller. In another study, Yang et al.^35^ combined the Lyapunov energy function, optimization design theory, and an iterative linear matrix to design optimal AGC control for two and five area interconnected power systems respectively. Further, a distributed LQR design is proposed by Vlahakis et al. for a large-scale multi-area power system to guarantee the overall stability of the network and disturbance rejections of power-load step variations^36^. This method maximizes stability margins and robust against load disturbances. Recently, optimal AGC control based on full state feedback control for two area interconnected power system with multiple generators is proposed to minimize the cost function and find the gain of the feedback matrix. This method show that optimal control methods are simple to design, offer low cost and robust performances. Besides, it is robust, reliable, against nonlinearities and modeling uncertainties associated with power systems^2^. Therefore, in this paper discrete optimal quadratic AGC based cost functional minimization is adopted for interconnected power systems.
  In the design of the optimal quadratic control, the most important first step is choice of the state and control weighting matrices Q and R. The weighting matrices Q and R play a vital role in determining the amount of steady state error, energy expenditure, and system performance^21^. In the literature, various approaches have been used for the selecting weighting matrices. The authors used a trial and error method based on the characteristics of the system state and controller^37^, Bryson’s rule in which the matrices Q and R, are taken as diagonal matrices with diagonal entries^38^ for selecting the weighting matrices. Also, Q and R were also selected based on the desired natural frequency and damping ratio of the closed-loop system for LQR based linear controllers^39^. Authors (Das et al.^40^) proposed using global optimization techniques, i.e. real coded genetic algorithm to optimally find the weighing matrices that are associated with LQR based PID control design. Besides, the controllability and observability indices of the system, functional minimization method (FMM), and engineering judgement based on the selection of all states of the power system. In the middle method, the optimal quadratic AGC control is designed to transfer the system state from an arbitrary initial state to the final state in an infinite amount of time so that the cost function is minimized. The weighting matrix Q was defined for the dynamic system under study by considering the excursion of area control errors, the excursion of the integral of area control errors and the excursion of the control vector about the steady state. This method was recently applied to a realistic model of optimal AGC in a real-time-time environment for two areas of control in the existence of communication delays^34^. The results revealed that the cost functional minimization method provides more realistic responses and it can be easily extended to the class of large dynamical systems i.e. power systems coupled with interaction signals. Besides, the discussed method, has the advantage of using part of the state variables to build the state weighting matrix, and so it is not requiring an observer to estimates the power system states.
3. *Research gap and motivation:* Based on the literature review, two studies have only been conducted for the functional minimization method for two-area control system with an identical non-reheat turbines. There is no research related to hydro and gas generators, as well as the combination of these two generators and other types of generators. Therefore, the discrete optimal quadratic automatic generation control (OQAGC) based on functional cost minimization is developed for interconnected power systems in this paper. Since it provides a simple and straightforward systematic approach, takes into account partially known state variables, and enables the cost function to optimize the state feedback gains matrix using the well-known linear quadratic theory, state and input weighting matrices are constructed using the functional minimization approach. Further, due to high precision, smaller controller size and adoptable and less noise, of the digital control methods^16,17^, the state space models are converted from continuous forms to discrete forms. Moreover, OQAGC compared with existing control approaches.
4. *Contribution*
  Considering the above literature, the significant contributions of this paper are:
  State space modeling of continuous and discrete forms of four area interconnected power systems are presented.
  A general cost functional minimization method is proposed for selecting discrete weighing matrices for N control areas and four area control with non-reheat, reheat, hydro and gas generators.
  State and input weighting matrices are developed based on functional minimization method to be implemented in the cost function for N control areas and four area control with non-reheat, reheat, hydro and gas generators.
  The steady-state Riccati Equation Matrix is implemented effectively to optimize the feedback control gains.
  Discrete optimal quadratic automatic generation control (OQAGC) is proposed based on functional minimization approach and an optimal control theory framework for N interconnected power systems with load disturbances.
  The performance of the OQAGC on power system dynamics has been studied with/without disturbances area control errors and sensitivity analysis the by considering four control areas. The results revealed that states deviations can converge to zero and that OQAGC shows robustness against disturbances. Thus, OQAGC is promising to be applied widely for more complex decentralized systems
  Finally, the formulation of the cost functional using the minimization approach is simple and easy to implement.
5. *Paper organization*: The paper is organized as follows: “The design of OQAGC control” section describes the design of the OQAGC controller in details. “A functional minimization method” section describes the developed functional minimization approach in a general framework for N control areas. Design of a OQAGC for the Four Area Power System is discussed in “Case study: design of a discrete OQAGC for four area power systems” section. The results and discussion are described in “Results and discussions” and “Conclusion” sections respectively.

## The design of OQAGC control

**Figure 1:**
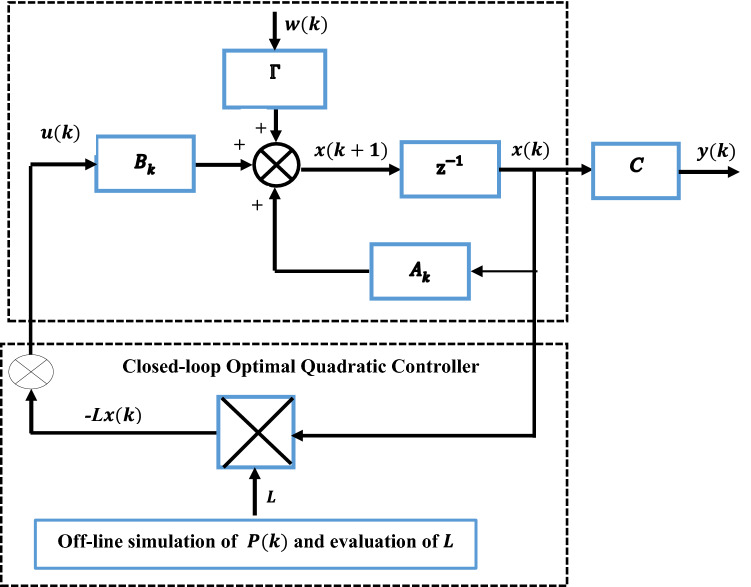
Closed-loop for an optimal control system with disturbances.

For the OQAGC control design the control system can be optimized using optimal theorem and lagrangian multipliers. Figure 1 depicts a closed-loop discrete optimal quadratic control system with a load disturbance signal $w(k)$ and an output signal $y\left(k\right).$

Consider the steady-state optimization problem for an interconnected power system with an N number of control areas and a cost function:

1 $$\mathrm{min}J= \frac{1}{2}\sum_{k={k}_{0}}^{\infty }{x}^{T}\left(k\right){Q}_{k}x\left(k\right)+{u}^{T}\left(k\right){R}_{k}u\left(k\right)$$

Subject to the equality constraint: a discrete linear control system

2 $$\begin{aligned} x\left(k+1\right) & ={A}_{k}x\left(k\right)+{B}_{k}u\left(k\right)+\Gamma w(k) \\x\left({k}_{0}\right)& ={x}_{0}, x\left({k}_{f}\right)={x}_{{k}_{f}}\end{aligned}$$

where ${Q}_{k}\in {\mathbb{R}}^{n\times n}$ and ${R}_{k}\in {\mathbb{R}}^{r\times r}$ are a symmetric positive semi-definite matrices $k={k}_{0},{k}_{1},\ldots , {k}_{f-1}$, ${k}_{f}=\infty$, $x(k)$ is ${n}{th}$ order state vector, $u(k)$ is ${r }^{th}$ order control vector and ${A}_{k}$ and ${B}_{k}$ are matrices of $n\times n$ and $n\times r$ dimensions respectively,$x({k}_{0})$ and $x({k}_{f})$ are the initial and final state conditions respectively $, w(k)$ is ${m}^{th}$ order disturbance input vector, and $\Gamma$ $\in {\mathbb{R}}^{n\times r}$ is a disturbance matrix.

A set of Lagrange multipliers $\lambda \left(1\right),$ $\lambda \left(2\right), \lambda \left(3\right)$,…,$\lambda \left({k}_{f}\right)$ are used to adjoin the cost functional in Eq. (1) and the equality constraint given in Eq. (2) so that the following augmented cost functional is minimized^41^.

3 $${J}_{a}=\frac{1}{2}\sum_{k={k}_{0}}^{k={k}_{f}=\infty }{[x}^{T}\left(k\right){Q}_{k}x\left(k\right)+{u}^{T}\left(k\right){R}_{k}u\left(k\right)]+\lambda (k+1)\left[{A}_{k}x\left(k\right)+{B}_{k}u(k)-x(k+1)\right]$$

where $\lambda (k +1)$ is the Lagrange multiplier. From the definition of the augmented cost functional, the Lagrangian and Hamiltonian functionals are defined as follows:

4 $$L(.)=\frac{1}{2}{x}^{T}\left(k\right){Q}_{k}x\left(k\right)+\frac{1}{2}{u}^{T}\left(k\right){R}_{k}u\left(k\right)+{\lambda }^{T}(k+1)\left[{A}_{k}x\left(k\right)+{B}_{k}u(k)-x(k+1)\right]$$

5 $$H(.)=\frac{1}{2}{x}^{T}\left(k\right){Q}_{k}x\left(k\right)+\frac{1}{2}{u}^{T}\left(k\right){R}_{k}u\left(k\right)+{\lambda }^{T}(k+1)\left[{A}_{k}x\left(k\right)+{B}_{k}u(k)\right]$$

The Lagrangian function in Eq. (4) and the Hamiltonian function in Eq. (5) are related by

6 $$L(.))=H \left(.\right)-\lambda (k+1)x(k+1)$$

The necessary conditions that minimize the augmented cost function, ${J}_{a}$ are obtained by taking the partial derivatives of the Hamiltonian functional with respect to the state $x(k),$ co-state $\lambda \left(k+1\right)$ and the transforming control $u(k)$ respectively as follows:

7 $$x(k)=\frac{\partial H(x\left(k-1\right), u\left(k-1\right),\lambda (k))}{\partial \lambda (k)}$$

at the next time interval $\left(k+1\right),$ the Eq. (7) can be written as

8 $$x\left(k+1\right)=\frac{\partial H\left(x\left(k\right), u\left(k\right),\lambda \left(k+1\right)\right)}{\partial \lambda \left(k+1\right)}=x\left(k+1\right)={A}_{k}x\left(k\right)+{B}_{k}u\left(k\right)$$

9 $$\lambda \left(k\right)=\frac{\partial H\left(u\left(k\right), x\left(k\right),\lambda \left(k+1\right)\right)}{\partial x\left(k\right)}={Q}_{k}x\left(k\right)+{A}_{k}^{T} \lambda \left(k+1\right)$$

10 $$0=\frac{\partial H(u\left(k\right), x\left(k\right),\lambda (k+1))}{\partial u(k)}={R}_{k}u\left(k\right)+{B}_{k }^{T}\lambda (k+1)$$

Then, the optimal open-loop $u(k)$ from Eq. (10) is obtained by

11 $$u\left(k\right)=-{R}_{k}^{-1}{B}_{k }^{T}\lambda (k+1)$$

Thus, by substituting the optimal open-loop control law into the state Eq. (8), the Hamiltonian system or the State and Co-state system is obtained as follows^42^:

12 $$\left[\begin{array}{c}x\left(k+1\right)\\ \lambda \left(k\right)\end{array}\right]=\left[\begin{array}{cc}{A}_{k}& {-E}_{k}\\ {Q}_{k}& {A}_{k }^{T}\end{array}\right]\left[\begin{array}{c}x\left(k\right)\\ \lambda \left(k+1\right)\end{array}\right]$$

where ${E}_{k}={B}_{k}{R}_{k}^{-1}{B}_{k }^{T}.$ Then, the steady-state Riccati Equation Matrix can be defined as

13 $$P(k)={Q}_{k}+{A}_{k}^{T}\left\{P(k+1)-P(k+1){\left[{B}_{k}^{T}P{B}_{k}+{R}_{k}\right]}^{-1}{B}_{k}^{T}P(k+1)\right\} {A}_{k}$$

Hence, the optimal feedback control law is given by the formula,

14 $$u\left(k\right)=-Lx\left(k\right)$$

where $L$ is the optimal feedback gain matrix and it is given by the formula,

15 $$L={\left[{R}_{k}+{B}_{k}^{T}P\left(k\right){B}_{k}\right]}^{-1}{B}_{k}^{T}P(k){A}_{k}$$

The optimal closed-loop system with disturbances is given by substituting the optimal feedback control law in the discrete model of the interconnected power system:

16 $$x\left(k+1\right)=\left[{A}_{k}-{B}_{k}\left({\left[{R}_{k}+{B}_{k}^{T}P\left(k\right){B}_{k}\right]}^{-1}{B}_{k}^{T}P(k){A}_{k}\right)\right]x\left(k\right)+\Gamma w(k)$$

where w is the vector of the load disturbance set-points. The closed-loop system will be stable if the real parts of Eigenvalues of the closed-loop matrix, $\left[{A}_{k}-{B}_{k}{[{R}_{k}+{B}_{k}^{T}P{B}_{k}]}^{-1}{B}_{k}^{T}P{A}_{k}\right]$ are located in the left half plane of the complex plane. Therefore, the solution of the steady-state Riccati Equation P is obtained iteratively starting from the initial $P(0)$ matrix. Then, at the steady-state the feedback control gain $K$ causes the optimal cost function J to be derived by

17 $$J(k)={x}^{T}\left(k\right)Px\left(k\right)$$

The mathematical equations from (1) to (17) were adopted from the books written by Ogata^41^ and Naido^42^.

## A functional minimization method

In this section, the functional minimization approach will be considered for developing the state and control-weighting matrices (${Q}_{k}$ and ${R}_{k}$) from Eq. (1). In this approach, the cost function is defined in terms of area control errors (ACEs), the integral of the area control errors (IACEs), and the summation of all control efforts. Then, the concept of partial derivatives is applied to each state and each control effort, and finally the summation of the partial derivatives of the states and the control efforts is combined to construct state weighting and control weighing matrices^34^. Accordingly, the requirements for the design are transformed into a cost function so that ACEs, $\sum {AEC}_{s}$, and the control vector $u(k)$ are minimized across all control areas, and the steady-state values of ACEs and $\sum {AEC}_{s}$ are zero, while the steady-state value of the control vector is constant.

Consider the cost function that meets the design requirements for N control areas $(i = \mathrm{1,2},\dots ,N)$

18 $$J=\frac{1}{2}\sum_{k={k}_{0}}^{\infty } \left(\left\{{\left({AEC}_{1}\right)}^{2}+{\left({AEC}_{2}\right)}^{2}+\dots +{\left({AEC}_{N}\right)}^{2}\right\}+\left\{{\left(\sum_{k={k}_{0}}^{\infty }{AEC}_{1}\right)}^{2}+{\left(\sum_{k={k}_{0}}^{\infty }{AEC}_{2}\right)}^{2}+\dots +{\left(\sum_{k={k}_{0}}^{\infty }{AEC}_{N}\right)}^{2}\right\}+\alpha \left\{{{u}_{1}}^{2}+{{u}_{2}}^{2}+\dots +{{u}_{N}}^{2}\right\} \right)$$

where ${ACE}_{1},{ACE}_{2,}\dots ,{ACE}_{N}$ are the area control errors and $\sum {AEC}_{1},\sum {AEC}_{2},\dots ,\sum {AEC}_{N}$ integral of area control errors, ${u}_{1},{u}_{2},..,{u}_{N}$ are the control vector signals for the entire interconnected power system and $\alpha$ is the constant factor used to limit the control effort of the controller.

For N control areas, the area control errors and their integrals can be defined as follows:

19 $$\begin{aligned}{ACE}_{1} & ={\beta }_{1}\Delta {f}_{1}+{\Delta P}_{tie1} \\ {ACE}_{2} & ={\beta }_{2}\Delta {f}_{2}+{a}_{12}{\Delta P}_{tie2}\\{ACE}_{3} & ={\beta }_{3}\Delta {f}_{3}+{a}_{13}{\Delta P}_{tie3} \\ \quad \quad \quad \quad \quad \quad \vdots \\ {ACE}_{N} & ={\beta }_{N}\Delta {f}_{N}+{a}_{1N}{\Delta P}_{tieN}\end{aligned}$$

and

20 $$\begin{aligned}I{ACE}_{1} & =\sum_{k={k}_{0}}^{\infty }({\beta }_{1}\Delta {f}_{1}+{\Delta P}_{tie1}) \\ I{ACE}_{2} & =\sum_{k={k}_{0}}^{\infty }{\beta }_{2}\Delta {f}_{2}+{{a}_{12}\Delta P}_{tie2} \\ {ACE}_{3} &=\sum_{k={k}_{0}}^{\infty }{\beta }_{3}\Delta {f}_{3}+{{a}_{13}\Delta P}_{tie3}\\ \quad \quad \quad \quad \quad \quad \vdots \\ I{ACE}_{N}&=\sum_{k={k}_{0}}^{\infty }({\beta }_{N}\Delta {f}_{N}+{a}_{1N}{\Delta P}_{tieN})\end{aligned}$$

where $\Delta {f}_{1}$, $\Delta {f}_{2}$, $\ldots ,\Delta {f}_{N}$ are the frequency deviations for area1, area 2,$\ldots$ and area N respectively, ${\Delta P}_{tie12}$ and ${\Delta P}_{tie1N}$ are the Tie line power deviations from area 1 to area 2,$\ldots$ and from area 1 to area N, ${a}_{12}=-1$ is the constant coefficient which changes the sign of the tie-line power towards area2, $I{ACE}_{1}$, $I{ACE}_{2},$ $I{ACE}_{3}$, …, $I{ACE}_{N}$ are the integrals of ${ACE}_{1},{ACE}_{2,}\dots ,{ACE}_{N}$.

By substituting Eqs. (19 and 20) into Eq. (18), we can find that the cost function expression is

21 $$J=\frac{1}{2}\sum_{k={k}_{0}}^{\infty }\left(\left\{{\left({\beta }_{1}\Delta {f}_{1}+{\Delta P}_{tie1}\right)}^{2}+{({\beta }_{2}\Delta {f}_{2}+{a}_{12}{\Delta P}_{tie2})}^{2}+\dots +{({\beta }_{N}\Delta {f}_{N}+{a}_{1N}{\Delta P}_{tieN})}^{2}\right\}+\left\{{\left(I{ACE}_{1}\right)}^{2}+{\left(I{ACE}_{2}\right)}^{2}+\dots +{\left(I{ACE}_{N}\right)}^{2}\right\}+\alpha \left\{{{u}_{1}}^{2}+{{u}_{2}}^{2}+\dots +{{u}_{N}}^{2}\right\}\right)$$

with regard to the cost functional

22 $$f(.)=\frac{1}{2} \left(\left\{{\left({\beta }_{1}\Delta {f}_{1}+{\Delta P}_{tie1}\right)}^{2}+{\left({\beta }_{2}\Delta {f}_{2}+{a}_{12}{\Delta P}_{tie2}\right)}^{2}+\dots +{\left({\beta }_{N}\Delta {f}_{N}+{a}_{1N}{\Delta P}_{tieN}\right)}^{2}\right\}+\left\{{\left(I{ACE}_{1}\right)}^{2}+{\left(I{ACE}_{2}\right)}^{2}+\dots +{\left(I{ACE}_{N}\right)}^{2}\right\}+\rho \left\{{{u}_{1}}^{2}+{{u}_{2}}^{2}+\dots +{{u}_{N}}^{2}\right\} \right)$$

We assume ${\Delta P}_{tie12}={\Delta P}_{tie13}=\dots ={\Delta P}_{tie1N}$ for N control areas. Also $u$ is the control vector signal. Now, the cost functional $f(.)$ can be used systematically to formulate the weighting matrices ${Q}_{k}$ and ${R}_{k}$. First, write the ACEs and IACEs in terms of the state variables and substitute them into the cost functional in Eq. (22). Hence, the cost function becomes a function of the state variables and control signals. The aim is to represent the functional Eq. (23) by a standard quadratic functional using the matrices ${Q}_{k}$ and ${R}_{k}$ for the state and control variables. The length of the state`s vector depends on the types and numbers of the power generators installed in each control area as well as the configuration of the Tie-lines. For instance, if we have N control areas and frequency deviation is selected as the first state in an N control area that contains non-reheat thermal, reheat, hydro and gas power plants, there will be one generator in each control area. Then, the states vector $${{\varvec{x}}}^{{\varvec{T}}}=\left|\begin{array}{ccccc}{{\varvec{x}}}_{1}& {{\varvec{x}}}_{2}& {{\varvec{x}}}_{3}& \cdots & {{\varvec{x}}}_{{\varvec{N}}}\end{array}\right|$$ for N control areas is given by:

23 $${{\varvec{x}}}^{{\varvec{T}}}=[\Delta {f}_{1}\Delta {P}_{T1} {P}_{G1} {IACE}_{1 }{\Delta P}_{tie12}\Delta {f}_{2} \cdots {IACE}_{2} {\Delta P}_{tie23} \cdots\Delta {f}_{N} \cdots {IACE}_{N}{ \Delta P}_{tieN-1}]$$

Secondly, we take the first partial derivatives of the functional $f(.)$ of the state variables and the control signal with respect to all states and the control signals, the ${Q}_{k}$ and ${R}_{k}$ matrices can be constructed. Then, the partial derivatives with respect to frequency states, the Tie-line states and the integral of the area control errors states can be obtained in the form of first order Ordinary Differential Equations (ODEs) as follows:

24 $$\begin{aligned} df(.)/d\Delta {f}_{1} & ={\beta }_{1}^{2}\Delta {f}_{1}+{\Delta P}_{tie1} \\ df(.)/d\Delta {f}_{2} &={\beta }_{2}^{2}\Delta {f}_{2}+{a}_{12}{\Delta P}_{tie2} \\ \quad \quad \quad \quad \quad \quad \vdots \\ df(.)/d\Delta {f}_{N}& ={\beta }_{N}^{2}\Delta {f}_{N}+{a}_{1N}{\Delta P}_{tieN} \end{aligned}$$

and

25 $$\begin{aligned} df(.)/d{\Delta P}_{tie12} & ={\beta }_{1}\Delta {f}_{1}+{\Delta P}_{tie1} \\ df(.)/d{\Delta P}_{tie23} & ={{a}_{12}\beta }_{2}\Delta {f}_{2}+{\Delta P}_{tie2} \\ \quad \quad \quad \quad \quad \quad \vdots \\ df(.)/d{\Delta P}_{tie(N-1)N}&={{a}_{1N}\beta }_{N}\Delta {f}_{N}+{\Delta P}_{tieN} \end{aligned}$$

In the same way, taking the partial derivatives with respect to the integral of the area control errors states, the following first-order ODEs are obtained:

26 $$\begin{aligned}df(.)/dI{ACE}_{1} &=I{ACE}_{1} \\ df(.)/dI{ACE}_{2}&=I{ACE}_{2} \\ \quad \quad \quad \quad \quad \quad \vdots \\ df(.)/dI{ACE}_{N} &=I{ACE}_{N} \end{aligned}$$

For solving the complex problem, in this paper the assumption is made that power demands on the interconnected power systems are the same, therefore tie-line power deviations are the same. Also, the first control area has non-reheat thermal power systems to simplify the state weighting matrix development process. Then, from Eqs. (24–26) the state weighing matrix ${Q}_{k}$ with dimension n by n is constructed as follows:

$${Q}_{k}=\left[\begin{array}{cccccccccccccc}\left(N+{a}_{12}^{2}\right)& {\beta }_{1}& 0& 0& (N+{a}_{12}){\beta }_{2}& 0& 0& 0& 0& 0& 0& \cdots & 0& 0\\ {\beta }_{1}& {\beta }_{1}^{2}& 0& 0& 0& 0& 0& 0& 0& 0& 0& \cdots & 0& 0\\ 0& 0& 0& 0& 0& 0& 0& 0& 0& 0& 0& \cdots & 0& 0\\ 0& 0& 0& 0& 0& 0& 0& 0& 0& 0& 0& \cdots & 0& 0\\ {a}_{12}{\beta }_{2}& 0& 0& 0& {\beta }_{2}^{2}& 0& 0& 0& 0& 0& 0& \cdots & 0& 0\\ \vdots & \vdots & \vdots & \vdots & \vdots & \vdots & \vdots & \vdots & \vdots & \vdots & \vdots & \cdots & 0& 0\\ {\beta }_{N}& 0& 0& 0& 0& 0& 0& 0& 0& \cdots & {\beta }_{N}^{2}& \cdots & 0& 0\\ 0& 0& 0& 0& 0& 0& 0& 1& 0& 0& 0& \cdots & 0& 0\\ 0& 0& 0& 0& 0& 0& 0& 0& 1& 0& 0& \cdots & 0& 0\\ \vdots & \vdots & \vdots & \vdots & \vdots & \vdots & \vdots & \vdots & \vdots & \vdots & \vdots & \vdots & 0& 0\\ 0& 0& 0& 0& 0& 0& 0& 0& 0& 0& 1& \cdots & 0& 0\\ 0& 0& 0& 0& 0& 0& 0& 0& 0& 0& 0& 1& 0& 0\\ \vdots & \vdots & \vdots & \vdots & \vdots & \vdots & \vdots & \vdots & \vdots & \vdots & 0& \vdots & \ddots & 0\\ 0& 0& 0& 0& 0& 0& 0& 0& 0& 0& 0& 0& 0& 1\end{array}\right]$$

*Proof:* The following steps illustrate the construction details of the matrix ${Q}_{k}$:

1. Rewrite and extend the functional $f(.)$ as follows:
  27 $$f\left(.\right)=\frac{1}{2} \left(\left\{\left[{\left({\beta }_{1}\Delta {f}_{1}\right)}^{2}+2{\beta }_{1}\Delta {f}_{1}{\Delta P}_{tie1}+{{\Delta P}_{tie1}}^{2}\right]+\left[{\left({\beta }_{2}\Delta {f}_{2}\right)}^{2}+2 {a}_{12}{\beta }_{2}\Delta {f}_{2}{\Delta P}_{tie2}+{{({a}_{12}\Delta P}_{tie2})}^{2}\right]+\dots +\left[{\left({\beta }_{n}\Delta {f}_{N}\right)}^{2}+2{a}_{1N}{\beta }_{N}\Delta {f}_{N}{\Delta P}_{tieN}+{{\Delta P}_{tieN}}^{2}\right]\right\}+\left\{{\left(I{ACE}_{1}\right)}^{2}+{\left(I{ACE}_{2}\right)}^{2}+\dots +{\left(I{ACE}_{N}\right)}^{2}\right\}+\alpha \left\{{{u}_{1}}^{2}+{{u}_{2}}^{2}+\dots +{{u}_{N}}^{2}\right\} \right)$$
2. Define the state variables i.e. ${x}_{1}={\Delta P}_{tie1}={\Delta P}_{tie2}=\dots ={\Delta P}_{tieN},$ ${x}_{2}=\Delta {f}_{1},$ ${x}_{3}=\Delta {P}_{T1}$, ${x}_{4}=\Delta {P}_{G1}$, ${x}_{5}=\Delta {f}_{2}$, ${x}_{6}=\Delta {P}_{T2}$, ${x}_{7}=\Delta {P}_{G2}$,…,${x}_{3N-1}=\Delta {f}_{N},$ ${x}_{3N}=\Delta {P}_{TN}$, ${x}_{3N+1}=\Delta {P}_{GN}$, ${x}_{3N+2}=I{ACE}_{1}$, ${x}_{3N+3}=I{ACE}_{2}\dots {x}_{4N+1}=I{ACE}_{N}.$ Then substitute them into Eq. (27) which becomes
  28 $$f\left(.\right)=\frac{1}{2}\left(\left\{\left[{\left({\beta }_{1}{x}_{2}\right)}^{2}+2{\beta }_{1}{x}_{2}{x}_{1}+{{x}_{1}}^{2}\right]+\left[{\left({\beta }_{2}{x}_{5}\right)}^{2}+2 {a}_{12}{\beta }_{2}{x}_{5}{x}_{1}+{{({a}_{12}x}_{1})}^{2}\right]+\dots +\left[{\left({\beta }_{N}{x}_{3N-1}\right)}^{2}+2{a}_{1N}{\beta }_{N}{x}_{3N-1}{x}_{1}+{{a}_{1N}}^{2}{{x}_{1}}^{2}\right]\right\} +\left\{{\left({x}_{3N+2}\right)}^{2}+{\left({x}_{3N+3}\right)}^{2}+\dots +{\left({x}_{4N+1}\right)}^{2}\right\}+\rho \left\{{{u}_{1}}^{2}+{{u}_{2}}^{2}+\dots +{{u}_{N}}^{2}\right\}\right)$$
3. Partially differentiating the functional $f\left(.\right)$ in Eq. (28) with respect to the state variables $$\begin{array}{ccccc}{x}_{1}& {x}_{2}& {x}_{3}& \cdots & {x}_{N}\end{array}$$, the following first order differential equations are obtained
  29 $$\begin{aligned} &{\begin{aligned} 1/2df(.)/d{x}_{1} &={\beta }_{1}{x}_{2}+{x}_{1}+{a}_{12}{\beta }_{2}{x}_{5}+{{a}_{12}}^{2}{x}_{1}+\dots +{{a}_{1N}\beta }_{N}{x}_{3N-1}+{{{a}_{1N}}^{2}x}_{1}\\ &=\left(1+{{a}_{12}}^{2}+\dots +{{a}_{1N}}^{2}\right){x}_{1}+{\beta }_{1}{x}_{2}{+a}_{12}{\beta }_{2}{x}_{5}+\dots +{{a}_{1N}\beta }_{N}{x}_{3N-1}\end{aligned}} \\ & 1/2df(.)/d{x}_{2} ={\beta }_{1}^{2}{x}_{2}+{\beta }_{1}{x}_{1}+0+\dots +0 \\ & 1/2df(.)/d{x}_{3} =0+0+\dots +0 \\ & 1/2df(.)/d{x}_{4}=0+0+\dots +0 \\ & 1/2df(.)/d{x}_{5} ={a}_{12}{\beta }_{2}{x}_{1}+{\beta }_{2}^{2}{x}_{5}+0+\dots +0 \\ & \vdots \quad \quad \quad \vdots \quad \quad \quad \vdots \quad \quad \quad \vdots \quad \quad \quad \vdots \quad \quad \quad \\ & 1/2df(.)/d{x}_{3N-1} ={{a}_{1N}{\beta }_{N}{x}_{1}+\beta }_{N}^{2}{x}_{3N-1}++0+\dots +0= \\ & 1/2df(.)/d{x}_{3N} =0+0+\dots +0 \\ & 1/2df(.)/d{x}_{3N+2} =0+0+\dots +0 \\ & 1/2df(.)/d{x}_{3N+1} ={x}_{3N+2}+0+0+\dots .+0 \\ & 1/2df(.)/d{x}_{3N+3} ={x}_{3N+2}+0+0+\dots .+0 \\ & \vdots \quad \quad \quad \quad \vdots \quad \quad \quad \quad \ddots \\ & 1/2df(.)/d{x}_{4N+1} =0+\dots +0+0+{x}_{4N+i} \end{aligned}$$
  A similar minimization concept can be applied to control energy expenditure where the first-order partial derivatives with respect to control input signals can be obtained as follows
  30 $$\begin{aligned} & df(.)/d{u}_{1}={u}_{1}+0 \\ & df(.)/d{u}_{2}=0+{u}_{2}+0 \\ & df(.)/d{u}_{3} =0+0+{u}_{3}+0 \\ & \vdots \quad \quad \quad \vdots \quad \quad \quad \quad \ddots \\ & df(.)/d{u}_{N} =0+0+0+\dots +{u}_{N} \end{aligned}$$
  As a result of Eq. (30), the control weighting matrix can be constructed as follows:
  31 $${R}_{k}=\left[\begin{array}{cccccccc}1& \cdots & 0& 0& 0& 0& 0& 0\\ 0& 1& \cdots & 0& 0& 0& 0& 0\\ 0& 0& 1& \cdots & 0& 0& 0& 0\\ 0& 0& 0& 1& 0& 0& 0& 0\\ 0& 0& 0& 0& 1& 0& 0& 0\\ 0& 0& 0& 0& 0& \ddots & 0& 0\\ 0& 0& 0& 0& 0& 0& 1& 0\\ 0& 0& 0& 0& 0& 0& 0& 1\end{array}\right]$$
  The ${R}_{k}$ matrix is obtained as an identity diagonal since it is assumed that there is only one generator in each control area and as a result, the participation factor is 1 per control area.

## Case study: design of a discrete OQAGC for four area power systems

### Dynamics of a four area power system

**Figure 2:**
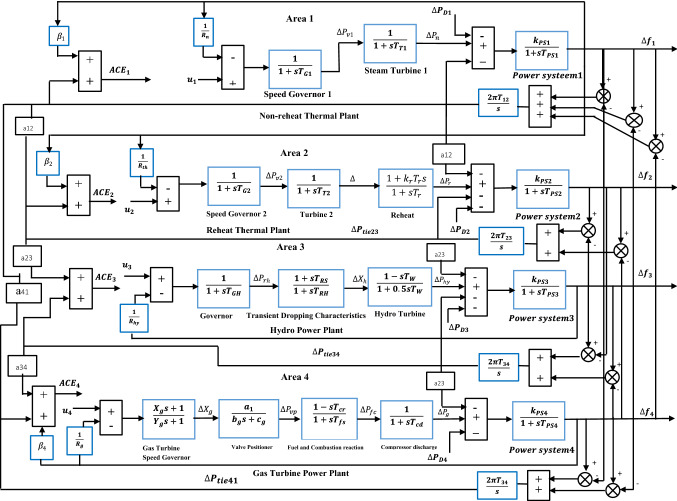
Four areas interconnected power system with area control errors.

The dynamics of electrical power systems are nonlinear in nature. However, for automatic generation control, the linearized model can be used to design the discrete optimal quadratic AGC controllers. Figure 2 shows a block diagram of the four area interconnected power systems. For simplicity, we made each area have non-reheat, reheat, hydro and gas generators respectively. Models and parameters for reheat, hydro, and gas were taken from different studies in the literature^43–50^.

**Figure 3:**
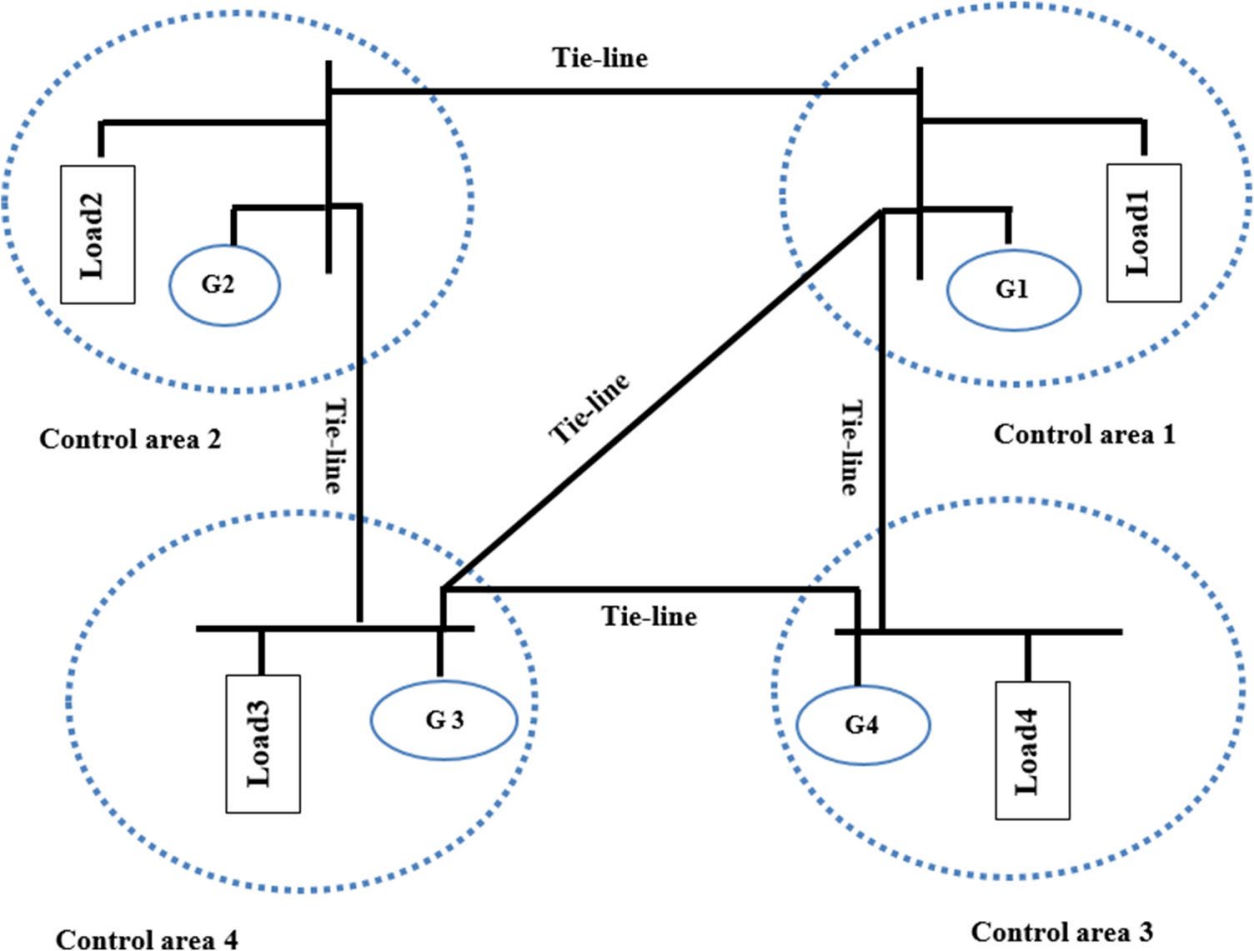
Proposed four area interconnected power system with tie-lines*.*

Based on the principles of load flow equations, the linear model of a tie-line for four area power system can be developed as shown in Fig. 3. This technique assumes that the tie-line resistance is negligible because of the high value of the inductance between the line $, {{\varvec{j}}{\varvec{X}}}_{{\varvec{i}}{\varvec{j}}}$. It is also assumed that the current ${{\varvec{I}}}_{{\varvec{i}}{\varvec{j}}}$ flows from control area 1 to control area 2, control area 2 to control area 3, control area 3 to control area 4, control area 4 to control area 1 and that the resistance of the tie-line transmission is zero $.$ The ${P}_{r1},$ ${P}_{r2}$, ${P}_{r3}$, and ${P}_{r4}$ are the rated power capacities of the control areas 1, 2 ,3 and 4 respectively. Then, the linear model of the Tie-line power deviations between areas (1 and 2), (2 and 3), (3 and 4) and (4 and 1) can be obtained as follows:

32 $$\begin{aligned} \Delta {P}_{tie12}(pu) & =\frac{2\pi {T}_{12}}{s}\left[3\Delta {f}_{1}-\Delta {f}_{2}-\Delta {f}_{3}-\Delta {f}_{4}\right] \\ \Delta {P}_{tie23}\left(pu\right) & =\frac{2\pi {T}_{23}}{s}\left[2\Delta {f}_{2}-\Delta {f}_{3}-\Delta {f}_{4}\right] \\ \Delta {P}_{tie34}(pu) & =\frac{2\pi {T}_{34}}{s}\left[2\Delta {f}_{3}-\Delta {f}_{2}-\Delta {f}_{4}\right] \\ \Delta {P}_{tie41}(pu) &=\frac{2\pi {T}_{41}}{s}\left[2\Delta {f}_{4}-\Delta {f}_{1}-\Delta {f}_{3}\right] \end{aligned}$$

Assuming the power system under study is a four area power systems i.e $. N=4$ as illustrated in Fig. 2. The state-space model of interconnected power system can be described by^36,37^:

33 $$\begin{aligned} \dot{x}\left(t\right) & =Ax\left(t\right)+Bu(t)+\Gamma w(t) \\ y&=Cx(t)+Du(t) \end{aligned}$$

where $x(t)\in {\mathbb{R}}^{n\times 1}$ is the state vector, $u(t)\in {\mathbb{R}}^{2\times 1}$ is the control input vector, and $w(t))\in {\mathbb{R}}^{2\times 1}$ is the load disturbances input vector, whereas the matrices $A,$ $B$, $\Gamma ,$ $C$ and $D$ have the appropriate dimensions. Equation (34) describes the variable vectors of a linear state-space model for a four-area control power system as follows:

34 $$\begin{aligned} {x}^{T} & =\begin{array}{c}[\Delta {f}_{1} \Delta {P}_{T1} \Delta {P}_{G1} {IACE}_{1} {\Delta P}_{tie12} \Delta {f}_{2} \Delta {P}_{T2} \Delta {P}_{TR} \Delta {P}_{G2} {IACE}_{2} {\Delta P}_{tie23} \Delta {f}_{3} \Delta {P}_{TW} \\ \Delta {P}_{TRH} \Delta {P}_{TGH} {IACE}_{3} {\Delta P}_{tie34} \Delta {f}_{4} {\Delta P}_{TCD} \Delta {P}_{TF} \Delta {P}_{YG} \Delta {P}_{bg} {IACE}_{4} {\Delta P}_{tie41}{]}^{T}\end{array} \\ {u}^{T} & ={\left[{u}_{1} {u}_{2} { u}_{3} {u}_{4}\right]}^{T} \\ {w}^{T} & ={\left[{\Delta P}_{d1}\boldsymbol{ }\boldsymbol{ }\boldsymbol{ }\boldsymbol{ }{\Delta P}_{d2} {\Delta P}_{d3} {\Delta P}_{d4}\right]}^{{\varvec{T}}} \\ {y}^{T} &={\left[\boldsymbol{ }{\Delta f}_{1}\boldsymbol{ }\boldsymbol{ }\boldsymbol{ }{\Delta f}_{2} {\Delta f}_{3} { \Delta f}_{4} { \Delta P}_{tie12} { \Delta P}_{tie23 } {\Delta P}_{tie34} { \Delta P}_{tie41}\right]}^{{\varvec{T}}} \end{aligned}$$

The matrices A, B, C, and D of the four interconnected power systems are shown below. These matrices were constructed by converting transfer functions into a set of first-order differential equations. The concept of modeling the interconnected power system as a state-space model can be found in the studies developed and represented by Rakhshani et al.^51^ and Deepak and Abraham^52^.

$$A=\left|\begin{array}{cccccccccccccccccccccccc}-\frac{1}{{T}_{p1}}& \frac{{k}_{ps1}}{{T}_{p1}}& 0& 0& \frac{{k}_{ps1}}{{T}_{p1}}& 0& 0& 0& 0& 0& 0& 0& 0& 0& 0& 0& 0& 0& 0& 0& 0& 0& 0& 0\\ 0& -\frac{1}{{T}_{t1}}& \frac{1}{{T}_{t1}}& 0& 0& 0& 0& 0& 0& 0& 0& 0& 0& 0& 0& 0& 0& 0& 0& 0& 0& 0& 0& 0\\ -\frac{1}{{T}_{g1}{R}_{ n}}& 0& -\frac{1}{{T}_{g1}}& 0& 0& 0& 0& 0& 0& 0& 0& 0& 0& 0& 0& 0& 0& 0& 0& 0& 0& 0& 0& 0\\ {\beta }_{1}& 0& 0& 1& 0& 0& 0& 0& 0& 0& 0& 0& 0& 0& 0& 0& 0& 0& 0& 0& 0& 0& 0& -1\\ {2\pi T}_{12}& 0& 0& 0& 0& -{2\pi T}_{12}& 0& 0& 0& 0& 0& {-2\pi T}_{12}& 0& 0& 0& 0& 0& {-2\pi T}_{12}& 0& 0& 0& 0& 0& 0\\ 0& 0& 0& 0& -\frac{{k}_{ps2}}{{T}_{p2}}& -\frac{1}{{T}_{p2}}& \frac{{k}_{ps2}}{{T}_{p2}}& 0& 0& 0& \frac{{k}_{ps2}}{{T}_{p2}}& 0& 0& 0& 0& 0& 0& 0& 0& 0& 0& 0& 0& 0\\ 0& 0& 0& 0& 0& 0& -\frac{1}{{T}_{r1}}& \frac{1}{{T}_{r1}}-\frac{{k}_{r1}}{{T}_{t2}}& \frac{1}{{T}_{t2}}& 0& 0& 0& 0& 0& 0& 0& 0& 0& 0& 0& 0& 0& 0& 0\\ 0& 0& 0& 0& 0& 0& 0& -\frac{1}{{T}_{t2}}& \frac{1}{{T}_{t2}}& 0& 0& 0& 0& 0& 0& 0& 0& 0& 0& 0& 0& 0& 0& 0\\ 0& 0& 0& 0& 0& -\frac{1}{{T}_{g2}{R}_{th}}& 0& 0& -\frac{1}{{T}_{g2}}& 0& 0& 0& 0& 0& 0& 0& 0& 0& 0& 0& 0& 0& 0& 0\\ 0& 0& 0& 0& -1& {\beta }_{2}& 0& 0& 0& 0& 1& 0& 0& 0& 0& 0& 0& 0& 0& 0& 0& 0& 0& 0\\ 0& 0& 0& 0& 0& {2\pi T}_{12}& 0& 0& 0& 0& 0& -{2\pi T}_{12}& 0& 0& 0& 0& 0& -{2\pi T}_{12}& 0& 0& 0& 0& 0& 0\\ 0& 0& 0& 0& 0& 0& 0& 0& 0& 0& -\frac{{k}_{ps3}}{{T}_{p3}}& -\frac{1}{{T}_{p3}}& \frac{{k}_{ps3}}{{T}_{p3}}& 0& 0& 0& \frac{{k}_{ps3}}{{T}_{p3}}& 0& 0& 0& 0& 0& 0& 0\\ 0& 0& 0& 0& 0& 0& 0& 0& 0& 0& 0& -\frac{2{T}_{r}}{{T}_{gh}{T}_{rh}{R}_{hy}}& -\frac{1}{{T}_{w}}& -\frac{2}{{T}_{rh}}& \frac{2}{{T}_{rh}}-\frac{{2T}_{R}}{{T}_{gh}}& 0& 0& 0& 0& 0& 0& 0& 0& 0\\ 0& 0& 0& 0& 0& 0& 0& 0& 0& 0& 0& -\frac{1}{{T}_{rh}{T}_{gh}{R}_{hy}}& 0& -\frac{1}{{T}_{rh}}& \frac{1}{{T}_{rh}}-\frac{{T}_{R}}{{T}_{gh}}& 0& 0& 0& 0& 0& 0& 0& 0& 0\\ 0& 0& 0& 0& 0& 0& 0& 0& 0& 0& 0& -\frac{1}{{T}_{gh}{R}_{hy}}& 0& 0& -\frac{1}{{T}_{gh}}& 0& 0& 0& 0& 0& 0& 0& 0& 0\\ 0& 0& 0& 0& 0& 0& 0& 0& 0& 0& -1& 0& 0& 0& 0& 0& 1& {\beta }_{3}& 0& 0& 0& 0& 0& 0\\ 0& 0& 0& 0& 0& -{2\pi T}_{23}& 0& 0& 0& 0& 0& {2\pi T}_{23}& 0& 0& 0& 0& 0& -{2\pi T}_{23}& 0& 0& 0& 0& 0& 0\\ 0& 0& 0& 0& 0& 0& 0& 0& 0& 0& 0& 0& 0& 0& 0& 0& -\frac{{k}_{ps4}}{{T}_{p4}}& -\frac{1}{{T}_{p4}}& \frac{{k}_{ps4}}{{T}_{p4}}& 0& 0& 0& 0& \frac{{k}_{ps4}}{{T}_{p4}}\\ 0& 0& 0& 0& 0& 0& 0& 0& 0& 0& 0& 0& 0& 0& 0& 0& 0& 0& -\frac{1}{{T}_{cd}}& \frac{1}{{T}_{cd}}& 0& 0& 0& 0\\ 0& 0& 0& 0& 0& 0& 0& 0& 0& 0& 0& 0& 0& 0& 0& 0& 0& -\frac{{T}_{cb}}{{T}_{f}{b}_{g}{R}_{g}}& 0& -\frac{1}{{T}_{f}}& \frac{1}{{T}_{f}}-\frac{{T}_{cb}}{{T}_{f}{Y}_{g}}& \frac{{T}_{cb}}{{T}_{f}}(\frac{1}{{Y}_{g}}-\frac{{X}_{g}{c}_{g}}{{b}_{g}{Y}_{g}}{)x}_{22}& 0& 0\\ 0& 0& 0& 0& 0& 0& 0& 0& 0& 0& 0& 0& 0& 0& 0& 0& 0& -\frac{{X}_{g}}{{Y}_{g}{b}_{g}{R}_{g}}& 0& 0& \frac{1}{{Y}_{g}}& \frac{1}{{Y}_{g}}-\frac{{X}_{g}{c}_{g}}{{b}_{g}{Y}_{g}}& 0& 0\\ 0& 0& 0& 0& 0& 0& 0& 0& 0& 0& 0& 0& 0& 0& 0& 0& 0& -\frac{{X}_{g}}{{b}_{g}{R}_{g}}& 0& 0& 0& -\frac{{c}_{g}}{{b}_{g}}& 0& 0\\ 0& 0& 0& 0& 0& 0& 0& 0& 0& 0& 0& 0& 0& 0& 0& 0& -1& 1& 0& 0& 0& 0& 0& {\beta }_{4}\\ 0& 0& 0& 0& 0& 0& 0& 0& 0& 0& 0& 0& 0& 0& 0& 0& 0& 0& 0& 0& 0& 0& 0 & {2\pi T}_{23}\end{array}\right|$$

$${{\varvec{B}}}^{{\varvec{T}}}=\left[\begin{array}{cccccccccccccccccccccccc}0& 0& \frac{{\boldsymbol{\alpha }}_{1}}{{{\varvec{T}}}_{{\varvec{g}}1}}& 0& 0& 0& 0& 0& 0& 0& 0& 0& 0& 0& 0& 0& 0& 0& 0& 0& 0& 0& 0& 0\\ 0& 0& 0& 0& 0& 0& 0& 0& \frac{{\boldsymbol{\alpha }}_{2}}{{{\varvec{T}}}_{{\varvec{g}}2}}& 0& 0& 0& 0& 0& 0& 0& 0& 0& 0& 0& 0& 0& 0& 0\\ 0& 0& 0& 0& 0& 0& 0& 0& 0& 0& 0& 0& \frac{2{\boldsymbol{\alpha }}_{3}}{{{{\varvec{T}}}_{{\varvec{r}}{\varvec{h}}}{\varvec{T}}}_{{\varvec{g}}{\varvec{h}}}}& \frac{{\boldsymbol{\alpha }}_{3}}{{{{\varvec{T}}}_{{\varvec{r}}{\varvec{h}}}{\varvec{T}}}_{{\varvec{g}}{\varvec{h}}}}& \frac{{\boldsymbol{\alpha }}_{3}}{{{\varvec{T}}}_{{\varvec{g}}{\varvec{h}}}}& 0& 0& 0& 0& 0& 0& 0& 0& 0\\ 0& 0& 0& 0& 0& 0& 0& 0& 0& 0& 0& 0& 0& 0& 0& 0& 0& 0& 0& 0& \frac{{\boldsymbol{\alpha }}_{4}{{\varvec{X}}}_{{\varvec{g}}}}{{{\varvec{b}}}_{{\varvec{g}}}{{\varvec{Y}}}_{{\varvec{g}}}}& \frac{{\boldsymbol{\alpha }}_{4}}{{{\varvec{b}}}_{{\varvec{g}}}}{{\varvec{u}}}_{4}& 0& 0\end{array}\right]$$

$${{\varvec{\Gamma}}}^{{\varvec{T}}}=\left[\begin{array}{cccccccccccccccccccccccc}-\frac{{{\varvec{k}}}_{{\varvec{p}}{\varvec{s}}1}}{{{\varvec{T}}}_{{\varvec{p}}1}}& 0& 0& 0& 0& 0& 0& 0& 0& 0& 0& 0& 0& 0& 0& 0& 0& 0& 0& 0& 0& 0& 0& 0\\ 0& 0& 0& 0& 0& -\frac{{{\varvec{k}}}_{{\varvec{p}}{\varvec{s}}2}}{{{\varvec{T}}}_{{\varvec{p}}2}}& 0& 0& 0& 0& 0& 0& 0& 0& 0& 0& 0& 0& 0& 0& 0& 0& 0& 0\\ 0& 0& 0& 0& 0& 0& 0& 0& 0& 0& 0& -\frac{{{\varvec{k}}}_{{\varvec{p}}{\varvec{s}}3}}{{{\varvec{T}}}_{{\varvec{p}}3}}& 0& 0& 0& 0& 0& 0& 0& 0& 0& 0& 0& 0\\ 0& 0& 0& 0& 0& 0& 0& 0& 0& 0& 0& 0& 0& 0& 0& 0& 0& -\frac{{{\varvec{k}}}_{{\varvec{p}}{\varvec{s}}4}}{{{\varvec{T}}}_{{\varvec{p}}4}}& 0& 0& 0& 0& 0& 0\end{array}\right]$$

$${\varvec{C}}=\left[\begin{array}{cccccccc}1& 0& 0& 0& 0& 0& 0& 0\\ 0& 1& 0& 0& 0& 0& 0& 0\\ 0& 0& 1& 0& 0& 0& 0& 0\\ 0& 0& 0& 1& 0& 0& 0& 0\\ 0& 0& 0& 0& 1& 0& 0& 0\\ 0& 0& 0& 0& 0& 1& 0& 0\\ 0& 0& 0& 0& 0& 0& 1& 0\\ 0& 0& 0& 0& 0& 0& 0& 1\end{array}\right] \;\; \text{and} \;\;{\varvec{D}}=0$$

### The design of discrete OQAGC for four area power system

The procedures that are described in “The design of OQAGC control” and “A functional minimization method” sections are used to design Optimal Quadratic AGC (OQAGC) controllers for the four control areas. Based on Eq. (21), the cost function for four areas is

35 $$J=\frac{1}{2}\sum_{k={k}_{0}}^{\infty }(\left\{{\left({AEC}_{1}\right)}^{2}+{\left({AEC}_{2}\right)}^{2}+{\left({AEC}_{3}\right)}^{2}+{\left({AEC}_{4}\right)}^{2}\right\}+\left\{{\left(\sum_{k={k}_{0}}^{\infty }{AEC}_{1}\right)}^{2}+{\left(\sum_{k={k}_{0}}^{\infty }{AEC}_{2}\right)}^{2}+{\left(\sum_{k={k}_{0}}^{\infty }{AEC}_{3}\right)}^{2}+{\left(\sum_{k={k}_{0}}^{\infty }{AEC}_{4}\right)}^{2}\right\}+\left\{{{u}_{1}}^{2}+{{u}_{2}}^{2}+{{u}_{3}}^{2}+{{u}_{4}}^{2}\right\})$$

**Figure 4:**
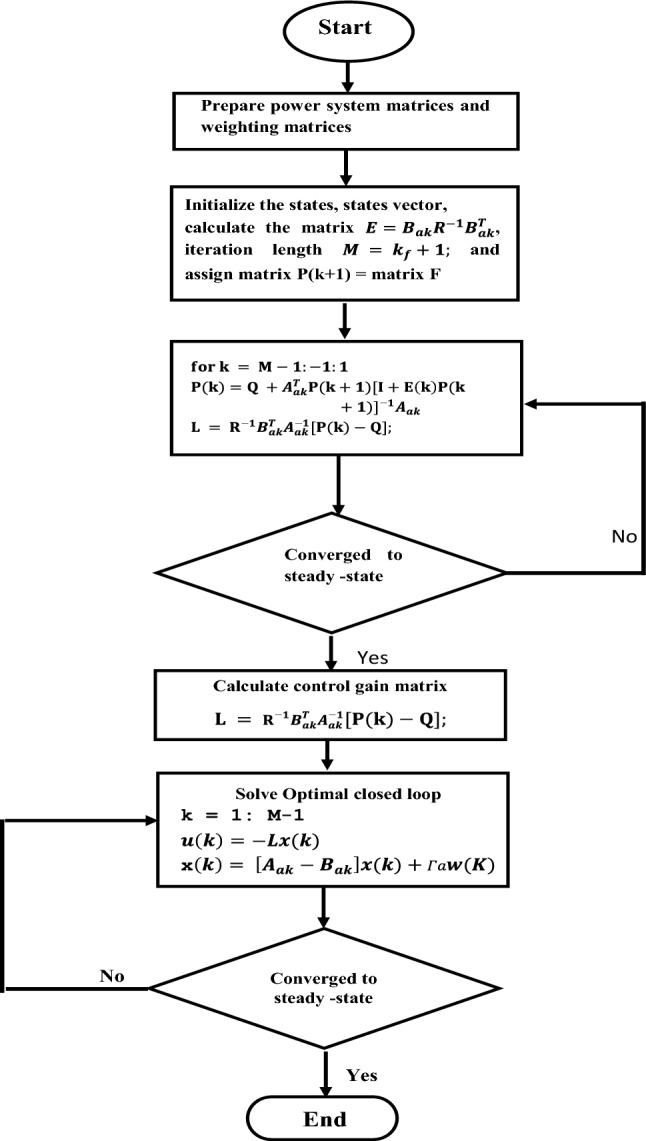
Flow chart of the OQAGC algorithm.

According to Eqs. (19 and 20), the area control errors (AEC1, AEC2, AEC3, and AEC4) and the integral of area control errors (IACE1, IACE2, IACE3, and IACE4) of the four control areas are defined as follows:

36 $$\begin{aligned} {ACE}_{1} & ={\beta }_{1}\Delta {f}_{1}+{\Delta P}_{tie1} \\ {ACE}_{2} & ={\beta }_{2}\Delta {f}_{2}+{\Delta P}_{tie2} \\{ACE}_{3} & ={\beta }_{3}\Delta {f}_{3}+{\Delta P}_{tie3} \\ {ACE}_{4} &={\beta }_{4}\Delta {f}_{4}+{\Delta P}_{tie4}\end{aligned}$$

and

37 $$\begin{aligned} {IACE}_{1} & =\sum_{k={k}_{0}}^{\infty }({\beta }_{1}\Delta {f}_{1}+{\Delta P}_{tie1}) \\ {IACE}_{2} & =\sum_{k={k}_{0}}^{\infty }({\beta }_{2}\Delta {f}_{2}+{\Delta P}_{tie2}) \\ I{ACE}_{3} &=\sum_{k={k}_{0}}^{\infty }({\beta }_{3}\Delta {f}_{3}+{\Delta P}_{tie3}) \\ I{ACE}_{4} & =\sum_{k={k}_{0}}^{\infty }({\beta }_{4}\Delta {f}_{4}+{\Delta P}_{tie4}) \end{aligned}$$

where ${\Delta P}_{tie1}$ , ${\Delta P}_{tie2},$ ${\Delta P}_{tie3}$ and ${\Delta P}_{tie4}$ are the sum of the tie-line deviations for the four areas respectively. From the block diagram shown in Fig. 4, the sum of tie-line power deviations in area 1, area 2, area 3 and area 4 respectively are obtained as follows:

38 $$\begin{aligned} {{\Delta P}_{tie1}}&={\Delta P}_{tie12+}+{a}_{34}{\Delta P}_{tie34} +{a}_{41}{\Delta P}_{tie41} \\ {\Delta P}_{tie2}&={a}_{12}{\Delta P}_{tie12}+{\Delta P}_{tie34} \\{\Delta P}_{tie3}&={a}_{23}{\Delta P}_{tie23}+{\Delta P}_{tie34} \\ {\Delta P}_{tie4}&={a}_{34}{\Delta P}_{tie34}+{\Delta P}_{tie41}\end{aligned}$$

Substituting ${\mathrm{ACE}}_{1}$, ${\mathrm{ACE}}_{2}, {\mathrm{ACE}}_{3}$,and ${\mathrm{ACE}}_{4} \mathrm{and} {\mathrm{IACE}}_{1}$, ${\mathrm{IACE}}_{2}$, ${\mathrm{IACE}}_{3},$ and ${\mathrm{IACE}}_{4}$) from Eqs. (36 and 37) to Eq. (35), the cost functional can be given by:

39 $$J=\frac{1}{2}\sum_{k={k}_{0}}^{\infty }{(\left({\beta }_{1}\Delta {f}_{1}+{\Delta P}_{tie1}\right)}^{2}+{({\beta }_{2}\Delta {f}_{2}+{\Delta P}_{tie2})}^{2}+{(\left({\beta }_{3}\Delta {f}_{3}+{\Delta P}_{tie3}\right)}^{2}+{(\left({\beta }_{4}\Delta {f}_{4}+{\Delta P}_{tie1}\right)}^{2})+\left\{{\left(I{ACE}_{1}\right)}^{2}+{\left(I{ACE}_{2}\right)}^{2}+{\left(I{ACE}_{3}\right)}^{2}+{\left(I{ACE}_{4}\right)}^{2}\right\}+\rho \left\{{{u}_{1}}^{2}+{{u}_{2}}^{2}+{{u}_{3}}^{2}+{{u}_{4}}^{2}\right\})$$

${\beta }_{1} , {\beta }_{2}$, ${\beta }_{3}$ and ${\beta }_{4}$ are the frequency bias for the four area power system respectively, ${\Delta P}_{tie12}$ , ${\Delta P}_{tie23}$, ${\Delta P}_{tie34}$ and ${\Delta P}_{tie41}$ are the tie-line deviations between areas 1 and 2, areas 2 and 3, areas 3 and 4 and areas 4 and 1 respectively and ${a}_{12}={a}_{23}={a}_{34}={a}_{41}=-1,$ is the constant coefficient that changes the sign of the tie-line power from one area to another. Hence, the vectors $x$ for four-area interconnected power systems with one type of generation unit in each area according to Eq. (34) is defined as follows

40 $${x}^{T}=\begin{array}{c}[{x}_{1} {x}_{2} {x}_{3} {x}_{4} {x}_{5} {x}_{6} {x}_{7} {x}_{8} {x}_{9} {x}_{10} {x}_{11} {x}_{12} {x}_{13} {x}_{14} \\ {x}_{15} {x}_{16} {x}_{17} {x}_{18} { x}_{19} {x}_{20} {x}_{21} {x}_{22} {x}_{23} {x}_{24}{]}^{T}\end{array}$$

where ${x}_{4}$, ${x}_{10},$ ${x}_{16}$ and ${x}_{23}$ are the integral of the Area control errors of areas 1, 2, 3 and 4 respectively. It can be observed that the functional of the cost function in Eq. (39) can be presented in the form of Eq. (41),where $f(.)$ is a functional of area control errors, integral of area control errors and control efforts $\left(f\left(.\right)=f\left(ACEs,IACEs,u\right)\right).$ By taking the partial derivatives of this function with respect to state variables as in Eq. (44) and control input signals in Eq. (45), then, the matrices ${Q}_{k}$ and ${R}_{k}$ can be derived based on the minimization approach of the function as follows:

1. Rewrite and extend the functional $f(.)$ as follows:
  41 $$f\left(.\right)=\frac{1}{2}(\left\{\left[{\left({\beta }_{1}\Delta {f}_{1}\right)}^{2}+2{\beta }_{1}\Delta {f}_{1}{\Delta P}_{tie1}+{{\Delta P}_{tie1}}^{2}\right]+\left[{\left({\beta }_{2}\Delta {f}_{2}\right)}^{2}+2 {\beta }_{2}\Delta {f}_{2}{\Delta P}_{tie2}+{{(\Delta P}_{tie2})}^{2}+{\left({\beta }_{3}\Delta {f}_{3}\right)}^{2}+2 {\beta }_{3}\Delta {f}_{3}{\Delta P}_{tie3}+{{(\Delta P}_{tie3})}^{2}+{\left({\beta }_{4}\Delta {f}_{4}\right)}^{2}+2 {\beta }_{4}\Delta {f}_{4}{\Delta P}_{tie4}+{{(\Delta P}_{tie4})}^{2}\right]\right\}+\left\{{\left(I{ACE}_{1}\right)}^{2}+{\left(I{ACE}_{2}\right)}^{2}+{\left(I{ACE}_{3}\right)}^{2}+{\left(I{ACE}_{4}\right)}^{2}\right\}+\rho \left\{{{u}_{1}}^{2}+{{u}_{2}}^{2}+{{u}_{3}}^{2}+{{u}_{4}}^{2}\right\})$$
  where $\rho$ is the participation factor. In this this paper we considered it 1 ( $\rho =1$) since we used only one type of generator in each area.
2. Define the state variables i.e. ${x}_{1}=\Delta {f}_{1},$ ${x}_{2}=\Delta {P}_{T1},$ ${x}_{3}=\Delta {P}_{G1}$, ${x}_{4}=I{ACE}_{1}$, ${x}_{5}={\Delta P}_{tie12}$, ${x}_{6}=\Delta {f}_{2}$, ${x}_{7}=\Delta {P}_{r1}$, ${x}_{8}=\Delta {P}_{T2}$,${x}_{9}=\Delta {P}_{G2,}$ ${x}_{10}=I{ACE}_{2}$, ${x}_{11}={\Delta P}_{tie23,}$ ${x}_{12}=\Delta {f}_{3}$, ${x}_{13}=\Delta {P}_{pen}$ ${x}_{14}=\Delta {P}_{HT}$*,*${x}_{15}=\Delta {P}_{Gh,}$ ${x}_{16}=I{ACE}_{3}$*,* ${x}_{17}={\Delta P}_{tie34}$,${x}_{18}=\Delta {f}_{4}$*, *${x}_{19}=\Delta {P}_{GT}$ ${x}_{20}=\Delta {P}_{GTF}$*,*${x}_{21}=\Delta {P}_{GTG,}$, ${x}_{22}=\Delta {P}_{VP,}{x}_{23}=I{ACE}_{4}$,${x}_{24}=\Delta {P}_{tie41}.$
3. Then substitute them into Eq. (40) without touching the tie-line variables which becomes
  42 $$f\left(.\right)=\frac{1}{2}(\left\{\left[{\left({\beta }_{1}{x}_{1}\right)}^{2}+2{\beta }_{1}{x}_{1}{\Delta P}_{tie1}+{{\Delta P}_{tie1}}^{2}\right]+\left[{\left({\beta }_{2}{x}_{6}\right)}^{2}+2 {\beta }_{2}{x}_{6}{\Delta P}_{tie2}+{{(\Delta P}_{tie2})}^{2}+{\left({\beta }_{3}{x}_{12}\right)}^{2}+2 {\beta }_{3}{x}_{12}{\Delta P}_{tie3}+{{(\Delta P}_{tie3})}^{2}+{\left({\beta }_{4}{x}_{18}\right)}^{2}+2 {\beta }_{4}{x}_{18}{\Delta P}_{tie4}+{{(\Delta P}_{tie4})}^{2}\right]\right\}+\left\{{\left({x}_{4}\right)}^{2}+{\left({x}_{10}\right)}^{2}+{\left({x}_{16}\right)}^{2}+{\left({x}_{23}\right)}^{2}\right\}+\rho \left\{{{u}_{1}}^{2}+{{u}_{2}}^{2}+{{u}_{3}}^{2}+{{u}_{4}}^{2}\right\})$$
4. Define the variables of the tie-lines power exchanges ${\Delta P}_{tie1},$ ${\Delta P}_{tie2}$, ${\Delta P}_{tie3}$ and ${\Delta P}_{tie4}$ from Eq. (37) and Fig. 3 as ${{\Delta P}_{tie1}=x}_{5}- {x}_{17}-{x}_{24}$, ${\Delta P}_{tie2}$=${-x}_{5}+ {x}_{11}$, ${\Delta P}_{tie3}={-x}_{11}+ {2x}_{17}$ and ${\Delta P}_{tie4}={-x}_{17}+ {x}_{24}$. Then substituting them into Eq. (41) which gives:
  43 $$\begin{aligned}f\left(.\right) & = \frac{1}{2}\left(\left\{\left[{{\beta }_{1}}^{2}{{x}_{1}}^{2}+2{\beta }_{1}{x}_{1}{(x}_{5}-{x}_{17}-{x}_{24})+{{x}_{5}}^{2}+{{x}_{17}}^{2}+{{x}_{24}}^{2}-2{x}_{5}{x}_{17}-2{x}_{5}{x}_{24}+2{x}_{17}{x}_{24}\right]\right.\right.\\&\quad\left.\left.+\left[{{\beta }_{2}}^{2}{{x}_{6}}^{2}+2 {\beta }_{2}{x}_{6}{(-x}_{5}+{x}_{11})+{{x}_{5}}^{2}-2{x}_{5}{x}_{11}+{{x}_{11}}^{2}]\right.\right.\right. \\&\quad \left.\left. \left. + \left[{{\beta }_{3}}^{2}{{x}_{12}}^{2}+2 {\beta }_{3}{x}_{12}\left(-{x}_{11}+{x}_{17}\right)+{{x}_{11}}^{2}-4{x}_{11}{x}_{17}+{4{x}_{17}}^{2}\right] \right.\right.\right.\\&\quad \left.\left.\left.+\left[{{\beta }_{4}}^{2}{{x}_{18}}^{2}+2 {\beta }_{4}{x}_{18} (-{x}_{17}+{x}_{24})+{{x}_{17}}^{2}-2{x}_{17}{x}_{24}+{{x}_{24}}^{2}\right]\right]\right\} \right. \\&\quad \left. + \left\{{{x}_{4}}^{2}+{{x}_{10}}^{2}+{{x}_{16}}^{2}+{{x}_{23}}^{2}\right\}+\rho \left\{{{u}_{1}}^{2}+{{u}_{2}}^{2}+{{u}_{3}}^{2}+{{u}_{4}}^{2}\right\}\right)\end{aligned}$$
5. When the functional $f(.)$ in Eq. (41) is partially differentiated with respect to the state variables $$\begin{array}{ccccc}{x}_{1}& {x}_{2}& {x}_{3}& \cdots & {x}_{24}\end{array}$$,), the following first order differential equations are obtained:
  44 $$\begin{aligned} 1/2df(.)/d{x}_{1} & ={{\beta }_{1}}^{2}{x}_{1}+{\beta }_{1}{x}_{5}-{\beta }_{1}{x}_{17}-{\beta }_{1}{x}_{24} \\ 1/2df(.)/d{x}_{2} &=0 \\ 1/2df(.)/d{x}_{3} & =0 \\ 1/2df(.)/d{x}_{4} & ={x}_{4} \\ 1/2df(.)/d{x}_{5} & ={\beta }_{1}{x}_{1}+2{x}_{5}-{\beta }_{2}{x}_{6}-{x}_{11}-{x}_{17}-{x}_{24} \\ 1/2df(.)/d{x}_{6} & =-{\beta }_{2}{x}_{5}+{{\beta }_{2}}^{2}{x}_{6}+{\beta }_{2}{x}_{11} \\ 1/2df(.)/d{x}_{7} & =0\\ 1/2df(.)/d{x}_{8} & =0 \\ 1/2df(.)/d{x}_{9} & =0 \\ 1/2df(.)/d{x}_{10} & ={x}_{10} \\ 1/2df(.)/d{x}_{11} & =-{x}_{5}+{\beta }_{2}{x}_{6}+2{x}_{11}-{\beta }_{3}{x}_{12}-2{x}_{17} \\ 1/2df(.)/d{x}_{12} & =-{\beta }_{3}{x}_{11}+{{\beta }_{3}}^{2}{x}_{12}++{\beta }_{3}{x}_{17} \\ 1/2df(.)/d{x}_{13} & =0 \\ 1/2df(.)/d{x}_{14} & =0 \\1/2df(.)/d{x}_{15}& =0 \\1/2df(.)/d{x}_{16} & ={x}_{16} \\ 1/2df(.)/d{x}_{17} & =-{{\beta }_{1}x}_{1}-{x}_{5}-{2x}_{11}+{\beta }_{3}{x}_{12}+3{x}_{17}-{\beta }_{4}{x}_{18} \\ 1/2df(.)/d{x}_{18} &={{\beta }_{4}}^{2}{x}_{18}-{\beta }_{4}{x}_{17}+{\beta }_{4}{x}_{24} \\1/2df(.)/d{x}_{19}&=0 \\1/2df(.)/d{x}_{20} & =0 \\ 1/2df(.)/d{x}_{21}& =0\\1/2df(.)/d{x}_{22} & =0 \\ 1/2df(.)/d{x}_{23} & ={x}_{23} \\1/2df(.)/d{x}_{24} & ={-{\beta }_{1}x}_{1}-{x}_{5}+{\beta }_{4}{x}_{18}+2{x}_{24} \end{aligned}$$

A similar concept can be used to control energy use, and the first-order partial derivatives with respect to control input signals can be found as follows:

45 $$\begin{aligned} df(.)/d{u}_{1}&={u}_{1} \\ df(.)/d{u}_{2} & ={u}_{2} \\ df(.)/d{u}_{3}&={u}_{3} \\df(.)/d{u}_{4} & ={u}_{4}\end{aligned}$$

The state weighing matrix, ${Q}_{k}$, and the control weighing matrix, ${R}_{k}$ can then be constructed using the above states partial (derivative Eq. (44) and the control partial derivatives from Eq. (45):

$${Q}_{k}=\left|\begin{array}{cccccccccccccccccccccccc}{\beta }_{1}^{2}& 0& 0& 0& {\beta }_{1}& 0& 0& 0& 0& 0& 0& 0& 0& 0& 0& 0& -{\beta }_{1}& 0& 0& 0& 0& 0& 0& -{\beta }_{1}\\ 0& 0& 0& 0& 0& 0& 0& 0& 0& 0& 0& 0& 0& 0& 0& 0& 0& 0& 0& 0& 0& 0& 0& 0\\ 0& 0& 0& 0& 0& 0& 0& 0& 0& 0& 0& 0& 0& 0& 0& 0& 0& 0& 0& 0& 0& 0& 0& 0\\ 0& 0& 0& 1& 0& 0& 0& 0& 0& 0& 0& 0& 0& 0& 0& 0& 0& 0& 0& 0& 0& 0& 0& 0\\ {\beta }_{1}& 0& 0& 0& 2& -{\beta }_{2}& 0& 0& 0& 0& -1& 0& 0& 0& 0& 0& -1& 0& 0& 0& 0& 0& 0& -1\\ 0& 0& 0& 0& -{\beta }_{2}& {\beta }_{2}^{2}& 0& 0& 0& 0& {\beta }_{2}& 0& 0& 0& 0& 0& 0& 0& 0& 0& 0& 0& 0& 0\\ 0& 0& 0& 0& 0& 0& 0& 0& 0& 0& 0& 0& 0& 0& 0& 0& 0& 0& 0& 0& 0& 0& 0& 0\\ 0& 0& 0& 0& 0& 0& 0& 0& 0& 0& 0& 0& 0& 0& 0& 0& 0& 0& 0& 0& 0& 0& 0& 0\\ 0& 0& 0& 0& 0& 0& 0& 0& 0& 0& 0& 0& 0& 0& 0& 0& 0& 0& 0& 0& 0& 0& 0& 0\\ 0& 0& 0& 0& 0& 0& 0& 0& 0& 1& 0& 0& 0& 0& 0& 0& 0& 0& 0& 0& 0& 0& 0& 0\\ 0& 0& 0& 0& -1& {\beta }_{2}& 0& 0& 0& 0& 2& -{\beta }_{3}& 0& 0& 0& 0& -2& 0& 0& 0& 0& 0& 0& 0\\ 0& 0& 0& 0& 0& 0& 0& 0& 0& 0& -{\beta }_{3}& {\beta }_{3}^{2}& 0& 0& 0& 0& {\beta }_{3}& 0& 0& 0& 0& 0& 0& 0\\ 0& 0& 0& 0& 0& 0& 0& 0& 0& 0& 0& 0& 0& 0& 0& 0& 0& 0& 0& 0& 0& 0& 0& 0\\ 0& 0& 0& 0& 0& 0& 0& 0& 0& 0& 0& 0& 0& 0& 0& 0& 0& 0& 0& 0& 0& 0& 0& 0\\ 0& 0& 0& 0& 0& 0& 0& 0& 0& 0& 0& 0& 0& 0& 0& 0& 0& 0& 0& 0& 0& 0& 0& 0\\ 0& 0& 0& 0& 0& 0& 0& 0& 0& 0& 0& 0& 0& 0& 0& 1& 0& 0& 0& 0& 0& 0& 0& 0\\ -{\beta }_{1}& 0& 0& 0& -1& 0& 0& 0& 0& 0& -2& {\beta }_{3}& 0& 0& 0& 0& 3& -{\beta }_{4}& 0& 0& 0& 0& 0& 0\\ 0& 0& 0& 0& 0& 0& 0& 0& 0& 0& 0& 0& 0& 0& 0& 0& -{\beta }_{4}& {\beta }_{4}^{2}& 0& 0& 0& 0& 0& {\beta }_{4}\\ 0& 0& 0& 0& 0& 0& 0& 0& 0& 0& 0& 0& 0& 0& 0& 0& 0& 0& 0& 0& 0& 0& 0& 0\\ 0& 0& 0& 0& 0& 0& 0& 0& 0& 0& 0& 0& 0& 0& 0& 0& 0& 0& 0& 0& 0& 0& 0& 0\\ 0& 0& 0& 0& 0& 0& 0& 0& 0& 0& 0& 0& 0& 0& 0& 0& 0& 0& 0& 0& 0& 0& 0& 0\\ 0& 0& 0& 0& 0& 0& 0& 0& 0& 0& 0& 0& 0& 0& 0& 0& 0& 0& 0& 0& 0& 0& 0& 0\\ 0& 0& 0& 0& 0& 0& 0& 0& 0& 0& 0& 0& 0& 0& 0& 0& 0& 0& 0& 0& 0& 0& 1& 0\\ -{\beta }_{1}& 0& 0& 0& -1& 0& 0& 0& 0& 0& 0& 0& 0& 0& 0& 0& 0& {\beta }_{4}& 0& 0& 0& 0& 0 & 2\end{array}\right|$$

and

$${R}_{k}=\left[\begin{array}{cccc}1& 0& 0& 0\\ 0& 1& 0& 0\\ 0& 0& 1& 0\\ 0& 0& 0& 1\end{array}\right]$$

The matrices of the power system and of the ${Q}_{k}$ and ${R}_{k}$ are used further for the calculation of the matrix of the Optimal Quadratic AGC controller by using the Eqs. (13–16). The flow chart of the OQAGC controller algorithm is shown in Fig. 4. The first parts of the algorithm are made to define the cost functional of the AGC problem based on the design requirements, secondly make use of the minimization of the functional of the cost function for selecting the state and control weighing matrices, thirdly, write the cost function in Eq. (35) in the form of Eq. (1). Then, through an iterative process the solution of discrete Riccati Eq. (13) can be found in the MATLAB environment^53^. The minimal values of the state and control weighing matrices as well as the solution of discrete Riccati Eq. (13) made it possible to calculate the optimal feedback gain matrix $L$ based on Eq. (14) which in turn optimises the closed-loop of the interconnected power system.

## Results and discussions

In this section, the developed discrete optimal quadratic controller is tested using MATLAB/Simulink simulation, namely the discrete optimal quadratic AGC controller with ACEs in a four-area power system. To demonstrate the feasibility of the optimal control technique, it is implemented in a discrete-time form with a sampling of 814 ms. The simulation study investigated the developed controller’s performance with and without disturbances. The parameters of the four-area power system are given in Table 1.

**Table 1:** The parameters of the four-area power systems^43–45^.

Parameter	Area 1	Area 2 $(i=2$)	Area 3	Area 4	Unit
${P}_{Ri}$	2000	2000	2000	2000	MW
${T}_{psi}$	20	$20$	20	20	s
${T}_{ti}$	0.3	0.3	0	0.23	s
${T}_{r1}$	0	0	1	0	s
${K}_{r1}$	0	0	0.3	0	–
${K}_{psi}$	$120$	120	120	120	$Hz/pu,MW$
${T}_{i}$	$0.0867$	0.0867	0.0867	0.0867	MW/Hz
${T}_{Gi}$	$0.08 \; \text{s}$	0.08	0.2	0.6	s
*Tcd*	0	0	0	0.2	s
*Tcr*	0	0	0	0.001	s
*bg*	0	0	0	0.05	s
*Cg*	0	0	0	1	mm
*Yg*	0	0	0	1	mm
*Tcb*	0	0	0	0.01	s
${T}_{rh}$	0	0	28.75	0	s
${T}_{w}$	0	0	1	0	s
${T}_{R}$	0	0	0.11		–
${R}_{i}$	$2.4$	2.4	2.4	2.4	$Hz/pu,MW$
${B}_{i}$	$0.425$	0.425	0.425	0.425	$pu MW/Hz$
${D}_{i}$	$0.0833$	0.0833			N/m s
$\Delta \delta$	${30}^{^\circ }$	${30}^{^\circ }$	${30}^{^\circ }$	${30}^{^\circ }$	Degree

### Calculation of the continuous and discrete matrices of the four-area power system

The state matrix $A$, the input matrix $B,$ and disturbance matrix $\Gamma$ from “Dynamics of a four area power system” section are obtained using the values of the parameters of non-reheat reheat, hydro and gas turbines represented in Table 1 as given in Eq. (44) below.

Due to the high speed of microcomputers, formulation and implementation of the optimal quadratic control problem in discrete form becomes an essential design step^15^. The continuous-time matrix A, B and E are converted to discrete-time matrices ${\mathrm{A}}_{k}$,${B}_{k}$ and ${\Gamma }_{k}$ using a one-step Euler discretization procedure^53^ and sampling time. In this procedure, the continuous-time and Discrete-time matrices are related to the formulas;

46 $$\begin{aligned} {\mathrm{A}}_{k} & =(I+T\times A) \\ {\mathrm{B}}_{k} &=(T\times B) \\{\Gamma }_{k} &=(T\times\Gamma ) \end{aligned}$$

where* I* is an identity matrix and *T* is the sampling period. Thus, the sampling period T was calculated based on Shannon's theorem and Nyquist's criteria to be 0.0814 s^54,55^.

$$A=\left[\begin{array}{cccccccccccccccccccccccc}-0.05 & 6& 0& 0& -6& 0& 0& 0& 0& 0& 0& 0& 0& 0& 0& 0& 0& 0& 0& 0& 0& 0& 0& 0\\ 0& 3.33 & -3.33 & 0& 0& 0& 0& 0& 0& 0& 0& 0& 0& 0& 0& 0& 0& 0& 0& 0& 0& 0& 0& 0\\ -5.21 & 0& -12.5& 6.79& 0& 0& 0& 0& 0& 0& 0& 0& 0& 0& 0& 0& 0& 0& 0& 0& 0& 0& 0& 0\\ 0.43 & 0& 0& 0& 1& 0& 0& 0& 0& 0& 0& 0& 0& 0& 0& 0& -1& 0& 0& 0& 0& 0& 0& 0\\ 1.632 & 0& 0& 0& 0& -0.545& 0& 0& 0& 0& 0& 0& 0& 0& 0& 0& 0& 0& 0& 0& 0& 0& 0& 0\\ 0& 0& 0& 0& 6& -0.0& 6& 0& 0& 0& 6& 0& 0& 0& 0& 0& 0& 0& 0& 0& 0& 0& 0& 0\\ 0& 0& 0& 0& 0& 0& -3.33& 3.33& 0& 0& 0& 0& 0& 0& 0& 0& 0& 0& 0& 0& 0& 0& 0& 0\\ 0& 0& 0& 0& 0& -1.563& 0& -1& 1& 2.038& 0& 0& 0& 0& 0& 0& 0& 0& 0& 0& 0& 0& 0& 0\\ 0& 0& 0& 0& 0& -5.21& 0& 0& 0& 6.7935& 0& 0& 0& 0& 0& 0& 0& 0& 0& 0& 0& 0& 0& 0\\ 0& 0& 0& 0& -1& 0.425& 0& 0& 0& 0& 1& 0& 0& 0& 0& 0& 0& 0& 0& 0& 0& 0& 0& 0\\ 0& 0& 0& 0& 0& 1.088& 0& 0& 0& 0& 0& -0.5438& 0& 0& 0& 0& 0& 0& 0& 0& 0& 0& 0& 0\\ 0& 0& 0& 0& 0& 0& 0& 0& 0& 0& 6& -0.05& 6& 0& 0& 0& -6& 0& 0& 0& 0& 0& 0& 0\\ 0& 0& 0& 0& 0& 0& 0& 0& 0& 0& 0& 0.7246& -1.6& 1.6696& 1.6696& -0.5687& 0& 0& 0& 0& 0& 0& 0& 0\\ 0& 0& 0& 0& 0& 0& 0& 0& 0& 0& 0& -0.3623& 0& -0.0348& -0.8348& 0.0023& 0& 0& 0& 0& 0& 0& 0& 0\\ 0& 0& 0& 0& 0& 0& 0& 0& 0& 0& 0& -2.0833& 0& 0& -5& 1.6349& 0& 0& 0& 0& 0& 0& 0& 0\\ 0& 0& 0& 0& 0& 0& 0& 0& 0& 0& -1& 0.425& 0& 0& 0& 1& -1& 0& 0& 0& 0& 0& 0& 0\\ 0& 0& 0& 0& -1& 0& 0& 0& 0& 0& 0& 1.0877& 0& 0& 0& 0& 0& -0.5438& 0& 0& 0& 0& 0& 0\\ 0& 0& 0& 0& 0& 0& 0& 0& 0& 0& 0& 0& 0& 0& 0& 0& 6& -0.0500& 6& 0& 0& 0& 0& -6\\ 0& 0& 0& 0& 0& 0& 0& 0& 0& 0& 0& 0& 0& 0& 0& 0& 0& 0& -5& 5& 0& 0& 0& 0\\ 0& 0& 0& 0& 0& 0& 0& 0& 0& 0& 0& 0& 0& 0& 0& 0& 0& -0.2174& 0& -4.347& 4.3043& -0.4783& 0.0728& 0\\ 0& 0& 0& 0& 0& 0& 0& 0& 0& 0& 0& 0& 0& 0& 0& 0& 0& -5& 0& 0& -1& -11& 1.6733& 0\\ 0& 0& 0& 0& 0& 0& 0& 0& 0& 0& 0& 0& 0& 0& 0& 0& 0& -8.3333& 0& 0& 0& -20& 2.7888& 0\\ 0& 0& 0& 0& 0& 0& 0& 0& 0& 0& 0& 0& 0& 0& 0& 0& 0& 0.4250& 0& 0& 0& 0& 0& 1\\ -0.544 & 0& 0& 0& -1& 0& 0& 0& 0& 0& 0& 0& 0& 0& 0& 0& 0& 1.0877& 0& 0& 0& 0& 0 & 0\end{array}\right]$$

$${{\varvec{B}}}^{{\varvec{T}}}=\left[\begin{array}{cccccccccccccccccccccccc}0& 0& 12.5& 0& 0& 0& 0& 0& 0& 0& 0& 0& 0& 0& 0& 0& 0& 0& 0& 0& 0& 0& 0& 0\\ 0& 0& 0& 0& 0& 0& 0& 3.75& 12.5& 0& 0& 0& 0& 0& 0& 0& 0& 0& 0& 0& 0& 0& 0& 0\\ 0& 0& 0& 0& 0& 0& 0& 0& 0& 0& 0& 0& -1.7391& 0.8696& 5& 0& 0& 0& 0& 0& 0& 0& 0& 0\\ 0& 0& 0& 0& 0& 0& 0& 0& 0& 0& 0& 0& 0& 0& 0& 0& 0& 0& 0& 0.5217& 12& 20& 0& 0\end{array}\right]$$

$${{\varvec{\Gamma}}}^{{\varvec{T}}}=\left[\begin{array}{cccccccccccccccccccccccc}-6\boldsymbol{ }\boldsymbol{ }& 0& 0& 0& 0& 0& 0& 0& 0& 0& 0& 0& 0& 0& 0& 0& 0& 0& 0& 0& 0& 0& 0& 0\\ 0& 0& 0& 0& 0& -6\boldsymbol{ }\boldsymbol{ }& 0& 0& 0& 0& 0& 0& 0& 0& 0& 0& 0& 0& 0& 0& 0& 0& 0& 0\\ 0& 0& 0& 0& 0& 0& 0& 0& 0& 0& 0& -6\boldsymbol{ }\boldsymbol{ }& 0& 0& 0& 0& 0& 0& 0& 0& 0& 0& 0& 0\\ 0& 0& 0& 0& 0& 0& 0& 0& 0& 0& 0& 0& 0& 0& 0& 0& 0& -6\boldsymbol{ }\boldsymbol{ }& 0& 0& 0& 0& 0& 0\end{array}\right]$$

Consequently, when the area control errors are taken into account, the discrete-time matrices in Eq. (46) are found.

$${A}_{k}=\left[\begin{array}{cccccccccccccccccccccccc}0.995& 0.488& 0& 0& -0.488& 0& 0& 0& 0& 0& 0& 0& 0& 0& 0& 0& 0& 0& 0& 0& 0& 0& 0& 0\\ 0& 0.729 & 0.271 & 0& 0& 0& 0& 0& 0& 0& 0& 0& 0& 0& 0& 0& 0& 0& 0& 0& 0& 0& 0& 0\\ -0.424 & 0& -0.018& 1.018& 0& 0& 0& 0& 0& 0& 0& 0& 0& 0& 0& 0& 0& 0& 0& 0& 0& 0& 0& 0\\ 0.035 & 0& 0& 1& 0.081& 0& 0& 0& 0& 0& 0& 0& 0& 0& 0& 0& -0.081& 0& 0& 0& 0& 0& 0& 0\\ 0.133 & 0& 0& 0& 1& -0.044& 0& 0& 0& 0& 0& 0& 0& 0& 0& 0& 0& 0& 0& 0& 0& 0& 0& 0\\ 0& 0& 0& 0& 0.488& 0.996& 0.488& 0& 0& 0& 0.488& 0& 0& 0& 0& 0& 0& 0& 0& 0& 0& 0& 0& 0\\ 0& 0& 0& 0& 0& 0& 0.729& 0.271& 0& 0& 0& 0& 0& 0& 0& 0& 0& 0& 0& 0& 0& 0& 0& 0\\ 0& 0& 0& 0& 0& -0.127& 0& 0.919& 0.081& 0.305& 0& 0& 0& 0& 0& 0& 0& 0& 0& 0& 0& 0& 0& 0\\ 0& 0& 0& 0& 0& -0.424& 0& 0& 1& 1.018& 0& 0& 0& 0& 0& 0& 0& 0& 0& 0& 0& 0& 0& 0\\ 0& 0& 0& 0& -0.081 & 0.035& 0& 0& 0& 1& 0.081& 0& 0& 0& 0& 0& 0& 0& 0& 0& 0& 0& 0& 0\\ 0& 0& 0& 0& 0& 0.089& 0& 0& 0& 0& 1& -0.044& 0& 0& 0& 0& 0& 0& 0& 0& 0& 0& 0& 0\\ 0& 0& 0& 0& 0& 0& 0& 0& 0& 0& 0.488& 0.996& 0.489& 0& 0& 0& -0.488& 0& 0& 0& 0& 0& 0& 0\\ 0& 0& 0& 0& 0& 0& 0& 0& 0& 0& 0& 0.059& 0.870& 0.136& 0.136 & -0.142& 0& 0& 0& 0& 0& 0& 0& 0\\ 0& 0& 0& 0& 0& 0& 0& 0& 0& 0& 0& -0.030& 0& 0.998& -0.068& 0.001& 0& 0& 0& 0& 0& 0& 0& 0\\ 0& 0& 0& 0& 0& 0& 0& 0& 0& 0& 0& -0.170& 0& 0& 0.593& 0.407& 0& 0& 0& 0& 0& 0& 0& 0\\ 0& 0& 0& 0& 0& 0& 0& 0& 0& 0& -0.081& 0.035& 0& 0& 0& 1& -0.081& 0& 0& 0& 0& 0& 0& 0\\ 0& 0& 0& 0& -1& 0& 0& 0& 0& 0& 0& 0.089& 0& 0& 0& 0& 1& -0.044& 0& 0& 0& 0& 0& 0\\ 0& 0& 0& 0& 0& 0& 0& 0& 0& 0& 0& 0& 0& 0& 0& 0& 0.488& 0.996& 0.488& 0& 0& 0& 0& -0.488\\ 0& 0& 0& 0& 0& 0& 0& 0& 0& 0& 0& 0& 0& 0& 0& 0& 0& 0& 0.593& 0.407& 0& 0& 0& 0\\ 0& 0& 0& 0& 0& 0& 0& 0& 0& 0& 0& 0& 0& 0& 0& 0& 0& -0.018& 0& 0.646& 0.350& -0.039& 0.043& 0\\ 0& 0& 0& 0& 0& 0& 0& 0& 0& 0& 0& 0& 0& 0& 0& 0& 0& -0.407& 0& 0& 0.919& -0.896& 0.977& 0\\ 0& 0& 0& 0& 0& 0& 0& 0& 0& 0& 0& 0& 0& 0& 0& 0& 0& -0.678& 0& 0& 0& -0.628& 1.628& 0\\ 0& 0& 0& 0& 0& 0& 0& 0& 0& 0& 0& 0& 0& 0& 0& 0& -0.081 & 0.035& 0& 0& 0& 0& 1& 0.081\\ -0.044 & 0& 0& 0& -1& 0& 0& 0& 0& 0& 0& 0& 0& 0& 0& 0& 0& 0.089& 0& 0& 0& 0& 0 & 1\end{array}\right]$$

$${B}_{k}^{T}=\left[\begin{array}{cccccccccccccccccccccccc}0& 0& 1.018& 0& 0& 0& 0& 0& 0& 0& 0& 0& 0& 0& 0& 0& 0& 0& 0& 0& 0& 0& 0& 0\\ 0& 0& 0& 0& 0& 0& 0& 3.75& 1.018& 0& 0& 0& 0& 0& 0& 0& 0& 0& 0& 0& 0& 0& 0& 0\\ 0& 0& 0& 0& 0& 0& 0& 0& 0& 0& 0& 0& -0.142& 0.071& 0.407& 0& 0& 0& 0& 0& 0& 0& 0& 0\\ 0& 0& 0& 0& 0& 0& 0& 0& 0& 0& 0& 0& 0& 0& 0& 0& 0& 0& 0& 0.043& 0.977& 1.628& 0& 0\end{array}\right]$$

$${\Gamma }_{k}^{T}=\left[\begin{array}{cccccccccccccccccccccccc}-0.488& 0& 0& 0& 0& 0& 0& 0& 0& 0& 0& 0& 0& 0& 0& 0& 0& 0& 0& 0& 0& 0& 0& 0\\ 0& 0& 0& 0& 0& -0.488 & 0& 0& 0& 0& 0& 0& 0& 0& 0& 0& 0& 0& 0& 0& 0& 0& 0& 0\\ 0& 0& 0& 0& 0& 0& 0& 0& 0& 0& 0& -0.488 & 0& 0& 0& 0& 0& 0& 0& 0& 0& 0& 0& 0\\ 0& 0& 0& 0& 0& 0& 0& 0& 0& 0& 0& 0& 0& 0& 0& 0& 0& -0.488 & 0& 0& 0& 0& 0& 0\end{array}\right]$$

### Simulation results of controlled systems

This section presents the simulation results of the developed control method using Eqs. (11 to 16). Referring to Eqs. (36 to 38), if ${\beta }_{1}={\beta }_{2}={\beta }_{3}={\beta }_{4}=0.425$ , ${a}_{12}=-1$ and using ${\Delta P}_{tie1} {\Delta P}_{tie2},$ ${\Delta P}_{tie3}$ and ${\Delta P}_{tie4}$ according to Eq. (38), the area control errors, the integral of the area control errors for four areas are obtained as follows

47 $$\begin{aligned} {ACE}_{1}&={0.425x}_{1}+{x}_{5}- {x}_{17}-{x}_{24} \\ {ACE}_{2} & =0.425{x}_{6}{-x}_{5}+ {x}_{11} \\ {ACE}_{3} & =0.425{x}_{12}{-x}_{11}+ {2x}_{17} \\ {ACE}_{4} &=0.425{x}_{18}{-x}_{17}+ {x}_{24}\end{aligned}$$

and

48 $$\begin{aligned} I{ACE}_{1} & =\sum_{k={k}_{0}}^{\infty }{(0.425x}_{1}+{x}_{5}- {x}_{17}-{x}_{24}) \\ I{ACE}_{2} & =\sum_{k={k}_{0}}^{\infty }(0.425{x}_{6}{-x}_{5}+ {x}_{11}) \\ I{ACE}_{3} & =\sum_{k={k}_{0}}^{\infty }(0.425{x}_{12}{-x}_{11}+ {2x}_{17}) \\ I{ACE}_{4} & =\sum_{k={k}_{0}}^{\infty }(0.425{x}_{18}{-x}_{17}+ {x}_{24}) \end{aligned}$$

Substituting Eqs. (47 and 48) into Eq. (43), the cost function becomes

49 $$\begin{aligned}J & = \frac{1}{2}\sum_{k={k}_{0}}^{\infty } \left(\left\{\left[0.180625{{x}_{1}}^{2}+0.85{x}_{1}{(x}_{5}-{x}_{17}-{x}_{24})+{{x}_{5}}^{2}+{{x}_{17}}^{2}+{{x}_{24}}^{2}-2{x}_{5}{x}_{17}-2{x}_{5}{x}_{24}+2{x}_{17}{x}_{24}\right]\right.\right. \\&\quad \left. \left. +\left[0.18062{{x}_{6}}^{2}+0.85{x}_{6}{(-x}_{5}+{x}_{11})+{{x}_{5}}^{2}-2{x}_{5}{x}_{11}+{{x}_{11}}^{2}] \right.\right.\right. \\&\quad \left.\left. \left. +[0.18062{{x}_{12}}^{2}+0.85{x}_{12}\left(-{x}_{11}+{x}_{17}\right)+{{x}_{11}}^{2}-4{x}_{11}{x}_{17}+{4{x}_{17}}^{2}]\right.\right.\right. \\&\quad \left.\left. \left.+0.18062{{x}_{18}}^{2}+0.85{x}_{18} (-{x}_{17}+{x}_{24})+{{x}_{17}}^{2}-2{x}_{17}{x}_{24}+{{x}_{24}}^{2}]\right]\right\}\right. \\&\quad \left.+\left\{{{x}_{4}}^{2}+{{x}_{10}}^{2}+{{x}_{16}}^{2}+{{x}_{23}}^{2}\right\}+\left\{{{u}_{1}}^{2}+{{u}_{2}}^{2}+{{u}_{3}}^{2}+{{u}_{4}}^{2}\right\} \right)\end{aligned}$$

where ${x}_{5}$, ${x}_{11}$, ${x}_{17}$ and ${x}_{24}$ are the tie-line power deviations between areas 1 and 2, 2 and 3, 3 and 4 and 4 and 1 respectively, ${x}_{1}$, ${x}_{6}$, ${x}_{12}$ and ${x}_{18}$ are frequency deviations of area-1 to 4 respectively,${x}_{4}$, ${x}_{10}$, ${x}_{16}$ and ${x}_{23}$ are the integrals of ACE 1 to 4 respectively.

The state and control weighting matrices ${Q}_{k}$ and ${R}_{k}$ are obtained on the basis of functional minimization procedure discussed in “The design of discrete OQAGC for four area power system” section and according to Eq. (44) as follows:

$${{\varvec{Q}}}_{{\varvec{k}}}=\left|\begin{array}{cccccccccccccccccccccccc}0.1806& 0& 0& 0& \boldsymbol{ }0.425& 0& 0& 0& 0& 0& 0& 0& 0& 0& 0& 0& -\boldsymbol{ }0.425& 0& 0& 0& 0& 0& 0& -\boldsymbol{ }0.425\\ 0& 0& 0& 0& 0& 0& 0& 0& 0& 0& 0& 0& 0& 0& 0& 0& 0& 0& 0& 0& 0& 0& 0& 0\\ 0& 0& 0& 0& 0& 0& 0& 0& 0& 0& 0& 0& 0& 0& 0& 0& 0& 0& 0& 0& 0& 0& 0& 0\\ 0& 0& 0& 1& 0& 0& 0& 0& 0& 0& 0& 0& 0& 0& 0& 0& 0& 0& 0& 0& 0& 0& 0& 0\\ \boldsymbol{ }0.425& 0& 0& 0& 2& -\boldsymbol{ }0.425& 0& 0& 0& 0& -1& 0& 0& 0& 0& 0& -1& 0& 0& 0& 0& 0& 0& -1\\ 0& 0& 0& 0& -\boldsymbol{ }0.425& 0.1806& 0& 0& 0& 0& \boldsymbol{ }0.425& 0& 0& 0& 0& 0& 0& 0& 0& 0& 0& 0& 0& 0\\ 0& 0& 0& 0& 0& 0& 0& 0& 0& 0& 0& 0& 0& 0& 0& 0& 0& 0& 0& 0& 0& 0& 0& 0\\ 0& 0& 0& 0& 0& 0& 0& 0& 0& 0& 0& 0& 0& 0& 0& 0& 0& 0& 0& 0& 0& 0& 0& 0\\ 0& 0& 0& 0& 0& 0& 0& 0& 0& 0& 0& 0& 0& 0& 0& 0& 0& 0& 0& 0& 0& 0& 0& 0\\ 0& 0& 0& 0& 0& 0& 0& 0& 0& 1& 0& 0& 0& 0& 0& 0& 0& 0& 0& 0& 0& 0& 0& 0\\ 0& 0& 0& 0& -1& \boldsymbol{ }0.425& 0& 0& 0& 0& 2& -\boldsymbol{ }0.425& 0& 0& 0& 0& -2& 0& 0& 0& 0& 0& 0& 0\\ 0& 0& 0& 0& 0& 0& 0& 0& 0& 0& -\boldsymbol{ }0.425& 0.1806& 0& 0& 0& 0& \boldsymbol{ }0.425& 0& 0& 0& 0& 0& 0& 0\\ 0& 0& 0& 0& 0& 0& 0& 0& 0& 0& 0& 0& 0& 0& 0& 0& 0& 0& 0& 0& 0& 0& 0& 0\\ 0& 0& 0& 0& 0& 0& 0& 0& 0& 0& 0& 0& 0& 0& 0& 0& 0& 0& 0& 0& 0& 0& 0& 0\\ 0& 0& 0& 0& 0& 0& 0& 0& 0& 0& 0& 0& 0& 0& 0& 0& 0& 0& 0& 0& 0& 0& 0& 0\\ 0& 0& 0& 0& 0& 0& 0& 0& 0& 0& 0& 0& 0& 0& 0& 1& 0& 0& 0& 0& 0& 0& 0& 0\\ -\boldsymbol{ }0.425& 0& 0& 0& -1& 0& 0& 0& 0& 0& -2& \boldsymbol{ }0.425& 0& 0& 0& 0& 3& -\boldsymbol{ }0.425& 0& 0& 0& 0& 0& 0\\ 0& 0& 0& 0& 0& 0& 0& 0& 0& 0& 0& 0& 0& 0& 0& 0& -\boldsymbol{ }0.425& 0.1806& 0& 0& 0& 0& 0& \boldsymbol{ }0.425\\ 0& 0& 0& 0& 0& 0& 0& 0& 0& 0& 0& 0& 0& 0& 0& 0& 0& 0& 0& 0& 0& 0& 0& 0\\ 0& 0& 0& 0& 0& 0& 0& 0& 0& 0& 0& 0& 0& 0& 0& 0& 0& 0& 0& 0& 0& 0& 0& 0\\ 0& 0& 0& 0& 0& 0& 0& 0& 0& 0& 0& 0& 0& 0& 0& 0& 0& 0& 0& 0& 0& 0& 0& 0\\ 0& 0& 0& 0& 0& 0& 0& 0& 0& 0& 0& 0& 0& 0& 0& 0& 0& 0& 0& 0& 0& 0& 0& 0\\ 0& 0& 0& 0& 0& 0& 0& 0& 0& 0& 0& 0& 0& 0& 0& 0& 0& 0& 0& 0& 0& 0& 1& 0\\ -\boldsymbol{ }0.425& 0& 0& 0& -1& 0& 0& 0& 0& 0& 0& 0& 0& 0& 0& 0& 0& \boldsymbol{ }0.425& 0& 0& 0& 0& 0\boldsymbol{ }& 2\end{array}\right|$$

The solution of the matrix difference Riccati equation ${P}_{k}$ according to Eq. (13) at steady-state is obtained with an iteration process starting from the initial condition $P\left(0\right)=0$ as:

$$P=\left|\begin{array}{cccccccccccccccccccccccc}17.9& 12.97& 2.21& 7.12 & 26.3& 9.983& 11.66& 7.42& 0.249& -11.61& 39.48& -4.76& 1.46& 18.1& -0.99& -23.6& 16.3& -2.1& 0.05& 1.29& 0.76& -0.29& -0.63& -4.75\\ 12.9& 13.22& 2.70& 9.15& 4.48& 0.566& 1.948& 2.66& 0.156& -7.717& 8.127& -2.04& -1.36& 6.26& -0.95& -10.5& 5.67& -3.2& -1.61& 0.04& 0.54& -0.28& -0.45& -5.29\\ 2.2& 2.70& 0.593& 2.08& -0.560& -0.399 & -0.184& 0.23& 0.025& -1.33& -0.120& -0.25& -0.39& 0.62& -0.18& -1.4& 0.46& -0.7& -0.45& -0.09& 0.08& -0.05& -0.08& -1.01\\ 6.5& 8.95& 2.06& 65.& -36.2& -22.74& -16.47& -5.47& -0.140& 26.54& -108.1& -12.5& -40.4& -78.7 & -0.63& 60.4& -61.3& -3.1& -1.96& -1.02& -1.2& 0.63& 16.00& -45.88\\ 27& 5.47 & -0.400& -32.5& 163.2& 92.38& 84.30& 36.78& 0.729& -52.17& 334.& -10.9& 57.2& 154.3& 0.49& -164& 115.9& 6.9& 5.69& 2.09& -0.54& 0.43& -10.22& 53.68\\ 11.3& 1.29& 0.296 & -21.6& 94.1& 70.28& 60.04& 23.33& 0.496& -16.60& 236.5& -9.42& 39.4& 94.6& 2.25& -102& 61.9& 3.2& 1.19& -0.62& -0.20& 0.02& -5.81& 36.7\\ 12.6& 2.54& -0.090& -14.2& 83.9& 58.47& 52.91& 22.63& 0.600& -12.81& 191.1& -10.9& 25.3& 72.4& 0.89& -78& 52.6& 2.2& 1.17& -0.16& -0.08& 0.01& -4.17& 26.09\\ 7.6& 2.6& 0.262& -4.65& 35.6& 22.16& 22.03& 11.45& 0.555& -4.33& 71.73& -5.20& 6.08& 24.6& -0.27& -27& 21.9& 0.27& 0.17& -0.09& -0.01& -0.01& -1.37& 8.96\\ 0.3& 0.156& 0.025& -0.129& 0.697& 0.471& 0.582& 0.550& 0.150& 0.994& 1.954& -0.151& -0.07& 0.247& -0.03& -0.3& 0.69& -0.01& -0.01& -0.01& -0.01& 0.1& -0.01& 0.26\\ -122 & -7.95& -1.36& 26.1& -54.8& -18.50& -14.47& -4.99& 0.984& 75.57& -111.6& -10.6& -42.7& -98.3& 1.07& 92& -70.7& -1.50& -1.73& -1.31& -0.65 & 0.26& 13.53& -37.49\\ 44.6& 10.7 & 0.230& -98.7& 346.3& 244.4& 201.0& 76.74& 2.043 & -105.3& 916.9& -3.04& 195.8& 414.0& 8.9& -446& 270.6& 8.06& 0.57& -3.80& -0.47& -0.17& -32.58& 173.56\\ -4.85& -2.06& -0.244& -12.4& -11.19& -9.348& -11.11& -5.336& -0.152& -10.60& -1.689& 21.5& 20.7& 10.6& 0.70& -16& 16.6& -5.9& -4.90& -1.52& 1.08& -0.69& -5.46& 16.27\\ 1.92 & -1.26& -0.391& -40.2& 60.85& 42.48& 27.67& 6.800& -0.067& -42.83& 206.1& 20.5& 86.3& 122& 8.33& -112& 47.3& 2.05& -1.98& -2.73& 0.66& -0.64& -15.17& 57.13\\ 21.1& 7.42& 0.750& -76.9& 170.4& 106.3& 82.66& 28.50& 0.297& -98.56& 451.8& 10.6& 121.6& 311& -4.37& -285& 191.6& 2.80& 0.6& -1.02& 0.08& -0.18& -34.40& 109.72\\ -1.27& -1.10& -0.206& -0.926& -0.421& 1.676& 0.312& -0.557& -0.040& 0.970& 7.196& 0.662& 8.46& -3.80& 3.60& 6.1& -14.2& 2.06& 0.8& -0.01& 0.15& -0.11& 0.6& -0.10\\ -27.3& -12.2& -1.64& 55.5& -178.3& -112.5& -88.28& -31.54& -0.416& 89.93& -476.1& -15.1& -108.2& -273& 5.89& 314& -218.4& 1.37& 2.11& 2.09& 1.76& -0.82& 20.76& -112.78\\ 19.4& 7.19& 0.666& -56.5& 125.4& 68.47& 59.55& 25.12& 0.744& -67.99& 285.7& 15.6& 42.4& 175.9& -13.7& -211& 240.5& -2.67& 8.23& 11.70& 3.39& -0.80& -17.56& 67.41\\ -2.34& -3.39& -0.743& -3.33& 6.135& 2.753& 1.689& -0.031& -0.013& -1.505& 6.796& -6.03& 2.32& 3.44& 2.12& 1.56& -3.08& 15.09& 12.57& 6.28& 2.28& -0.86& 15.30& -0.94\\ -0.186& -1.76& -0.471 & -2.21& 5.049& 0.892& 0.789& -0.068& -0.007& -1.798& -0.304& -4.99& -1.72& 1.49& 0.90& 2.1& 7.95& 12.51& 13.07& 8.67& 3.99& -1.59& 13.98& -8.14\\ 1.14& -0.06& -0.114& -1.20& 1.737& -0.764& -0.356& -0.230& -0.006& -1.359& -4.254& -1.59& -2.57& -0.45& 0.01& 2.05& 11.5& 6.19& 8.62& 7.47& 5.09& -2.26& 10.42& -9.59\\ 0.727& 0.511& 0.078& -1.38& -0.494& -0.160& -0.065& -0.026& -0.004& -0.749& -0.255& 1.07& 0.726& 0.611& 0.10& 1.4& 3.67& 2.19& 3.90& 5.03& 1050& -5.55& 18.36& -4.02\\ -0.286& -0.266& -0.048& 0.703& 0.374& -0.014& -0.021& -0.011& 0.002& 0.314& -0.325& -0.69& -0.67& -0.43& -0.08& -0.58& -0.97& -0.82& -1.54& -2.23& -5.60& 2.98& -9.70& 1.39\\ -0.907& -0.516& -0.077& 16.6& -12.78& -7.582& -5.582& -1.853& -0.018& 14.32& -39.61& -5.69& -15.9& -37.9& 1.01& 26.2& -21.9& 15.64& 14.34& 10.69& 18.49& -9.76 & 68.09& -14.77\\ -3.50& -4.64& -0.920& -43.8 & 57.55& 39.10& 28.82& 10.24& 0.284 & -36.40& 180.03& 16.02& 55.20& 102.8& 0.10& -110& 67.87& -0.55& -7.83& -9.44& -4.19& 1.51& -12.59& 74.68\end{array}\right|$$

Based on Eq. (15), the constant feedback gain matrix values for the closed-loop system at steady-state are computed as follows:

$$L=\left[\begin{array}{cccccccccccccccccccccccc}1.149& 1.868& 0.449& 1.729& -1.317& -0.500& -0.428& -0.038& 0.012& -0.810& -0.920& -0.089& -0.299& 0.158& -0.120& -0.702 & -0.104& -0.527& -0.395& -0.156& 0.031& -0.027& -0.036& -0.597\\ 1.405& 0.683& 0.087& -0.640& 5.211& 3.072& 3.342& 2.169& 0.253& 0.550& 10.406& -0.832& 0.374& 2.920 & -0.117& -3.420& 3.462& -0.026& -0.011& -0.022& -0.005& 0.001 & -0.1600& 1.285\\ 0.586& 0.240& 0.027& 0.030& 2.159& 1.309& 1.370& 0.600& 0.011& -0.300& 2.639& -1.606& -0.930& 1.909& -0.175& -0.712& 1.218& 0.516& 0.591& 0.349& 0.019& 0.016& -0.028& -0.782\\ 0.299& 0.150& 0.016& -0.193& -0.053& -0.237& -0.146& -0.053& -0.001 & -0.203& -0.844& 0.132& -0.326& -0.179& -0.047& 0.350& 2.096& 0.497& 1.084& 1.243& 1.367& -0.669& 2.162& -1.683\end{array}\right]$$

Hence, the optimal state feedback control law for a discrete Optimal Quadratic AGC controller is:

50 $$u(k)=-Lx(k)$$

and, the corresponding closed-loop system’s Eigenvalues are found as given in Table 2. All the real parts of Eigenvalues of the discrete closed-loop are less than 1 which guarantees the system stability associated with oscillation due to the imaginary parts of Eigenvalues.

**Table 2:** The systems’ states and their corresponding closed-loop Eigenvalues.

State	${x}_{1}$	${x}_{2}$	${x}_{3}$	${x}_{4}$	${x}_{5}$	${x}_{6}$	${x}_{7}$	${x}_{8}$	${x}_{9}$	${x}_{10}$	${x}_{11}$	${x}_{12}$
Eigenvalue	$-0.62$	$-0.08$	0.43	$0.8 + 0.34i$	0.8 − 0.34i	$0.51$	$0.9 + 0.29i$	$0.9- 0.29i$	$0.9 + 0.22i$	0.9 − 0.217i	0.8 + 0.158i	0.8 − 0.16i

State	${x}_{13}$	${x}_{14}$	${x}_{15}$	${x}_{16}$	${x}_{17}$	${x}_{18}$	${x}_{19}$	${x}_{20}$	${x}_{21}$	${x}_{22}$	${x}_{23}$	${x}_{24}$
Eigenvalue	0.97 + 0.07i	0.97 − 0.07i	0.94	0.93 + 0.03i	0.93 − 0.03i	0.9 + 0.04i	0.9 − 0.04i	0.9	0.7	0.8	0.73	0.73

The simulations were conducted for two different cases which are discussed below:

*Case 1:* load disturbances ${w}^{T}=0.01$ are imposed on the power system at t = 0. The parameters of four area control are utilized. The initial tie-line power is set at 0 MW (0pu) and the initial values of the frequency deviations in four areas are set at 0.0 Hz for each area. The Discrete Optimal Quadratic AGC controller Eq. (48), is assumed to act on the four-area state model when the area control errors are considered. The simulation is run in the MATLAB environment for 1800 iterations with ${k}_{0}=0$ and ${k}_{f}=1800.$

*Case 2:* Step load disturbances ${w}^{T}={\left[{\Delta P}_{D1}\boldsymbol{ }\boldsymbol{ }\boldsymbol{ }\boldsymbol{ }{\Delta P}_{D2} { \Delta P}_{D3} {\Delta P}_{D4}\right]}^{{\varvec{T}}}$ are added simultaneously to each area. The initial tie-line power is set at 0 MW (0pu) and the initial values of the frequency deviations are set at 0.0 Hz for each area. The Discrete Optimal Quadratic AGC controller is assumed to act on the four-area state model when the area control errors are considered. We considered this mainly to test** t**he feasibility of the developed controller and its robustness against disturbances. The simulation is run in the MATLAB environment for 1800 iterations with ${k}_{0}=0$ and ${k}_{f}=1800$. The magnitude of the load disturbances for the four areas is ${\Delta P}_{D1}=10$ MW (0.01pu), ${\Delta P}_{D2}=10$ MW (0.01pu) , ${\Delta P}_{D3}=10$ MW (0.01pu) and ${\Delta P}_{D4}=10$ MW (0.01pu) respectively.

**Figure 5:**
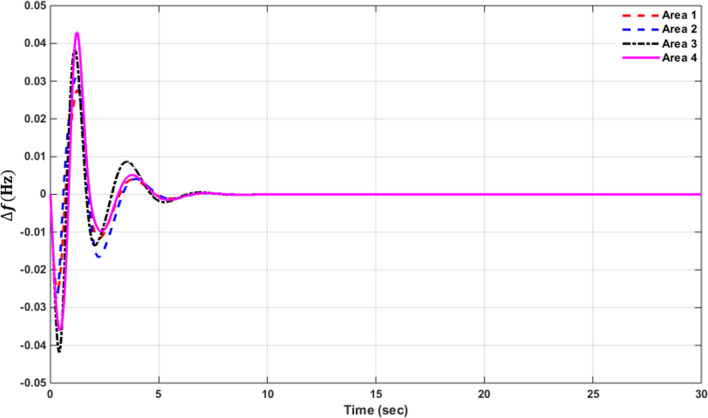
Frequency deviations in four control areas without disturbances.

Figure 5 shows the frequency deviations of OQAGC when area control errors are considered in areas 1, 2, 3 and 4 respectively. From the simulations, it can be observed that the frequency deviations converge to zero in less than 8 s due to area control errors. It is noted that the frequency deviations undershot and overshoot with an oscillatory response before the signals return to zero in the steady state.

**Figure 6:**
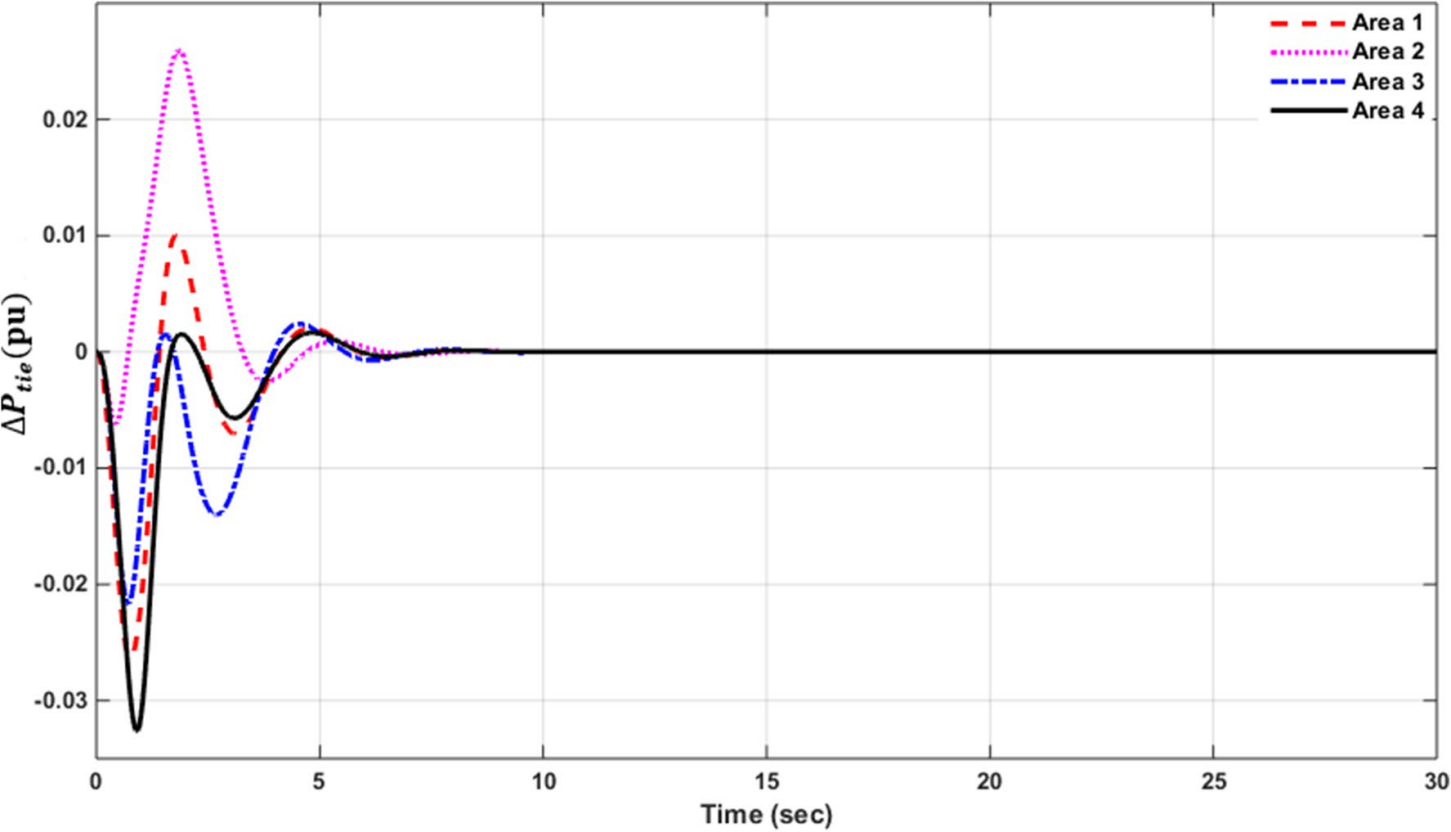
Tie-line power deviations in four areas when no disturbance is imposed.

The OQAGC model with area control errors provides that deviation of tie-line power for the four areas shown in Fig. 6. It is observed that the tie-line deviation converges to zero in less than 8 s. Tie-line power deviations for the four areas overshoot and undershot around the zero with oscillation before the signals return to zero. Furthermore, we can see that the reheat turbine thermal power system was associated with the highest overshoot, while the hydropower system was associated with the smallest overshoot. For gas power systems, the amplitude of the overshoot is constant compared to other power systems.

**Figure 7:**
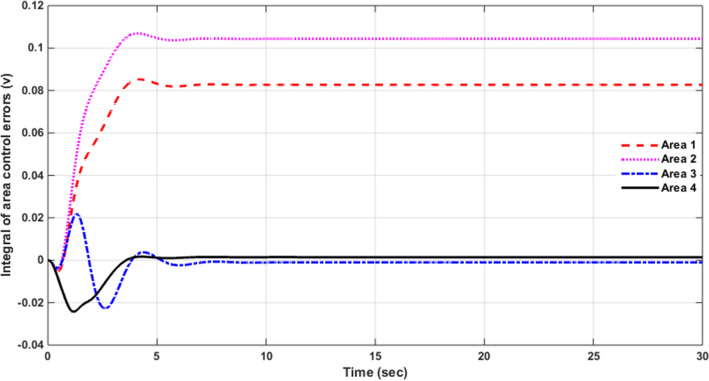
Integral of area control errors in four areas with no disturbances.

Figure 7 shows the integral of area control errors for four area power systems when area control errors are considered. It can be observed that the integral of area control errors converges to zero in less than 9 s. The integral of area control error deviations for the four areas overshoot and undershot around the zero with oscillation before the signals return to zero.

These results reveal that the discrete OQAGC controller with ACEs can bring the fluctuations associated with frequency deviations, tie-line deviations, and the integral of area control errors to their nominal values within a reasonable time. In addition, the frequency deviations, tie-line deviations, and the integral of area control errors for the four areas are associated with overshoot and undershot around the zero with oscillation during the transient period. Moreover, the non-reheat and reheat turbine thermal power systems were associated with the highest overshoot for the integral of the area control areas, while the hydro and gas power systems were associated with the smallest overshoot for the integral of the area control areas.

**Figure 8:**
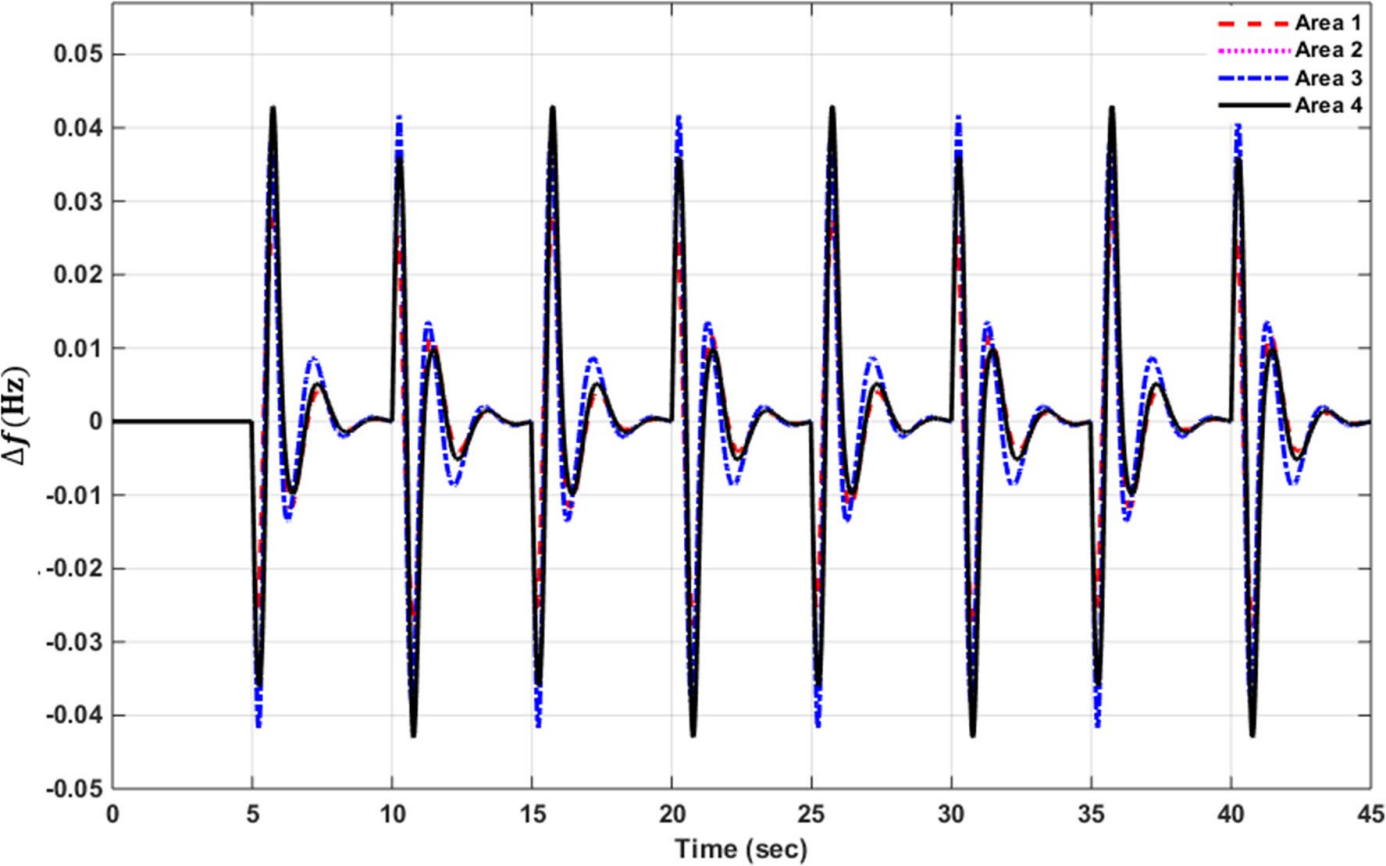
Frequency deviations of OQAGC for four areas with load disturbances.

In case 2 simulations, 1% power loads are added simultaneously to the system random 5 s intervals since the load demands can change at any time during the day. Figure 8 shows the plots of the frequency deviations for area 1 to 4 respectively, when area control errors are considered. It is observed that the OQAGC controller rejects the disturbances significantly at 4 s or more. The discrete OQAGC has the greatest overshoot and undershoots in the frequency deviations $\Delta {f}_{3}$ and $\Delta {f}_{4}$ while all frequency deviations have same disturbance rejection (robustness) and same settling time.

**Figure 9:**
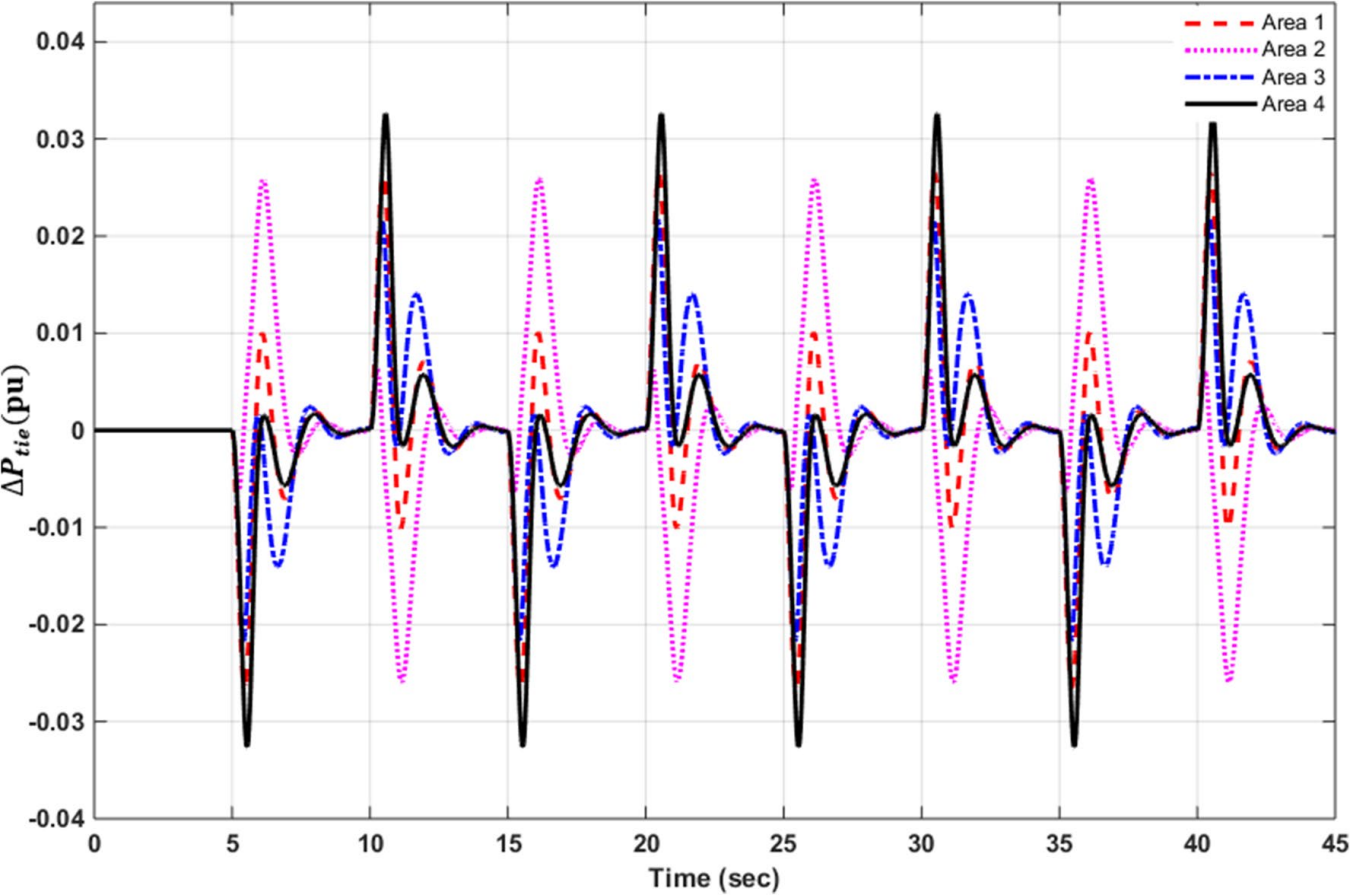
OQAGC Tie-line deviations for four area power systems with load disturbances.

The deviations in the tie-line power for the four areas are shown in Fig. 9. The tie-line power deviation between areas 2 and 3 has the shortest undershot among tie-line deviations while the tie-line power deviation between areas 4 and 1 has the fastest disturbance rejection (robustness) compared to other ones in the presence of large magnitude load disturbances.

**Figure 10:**
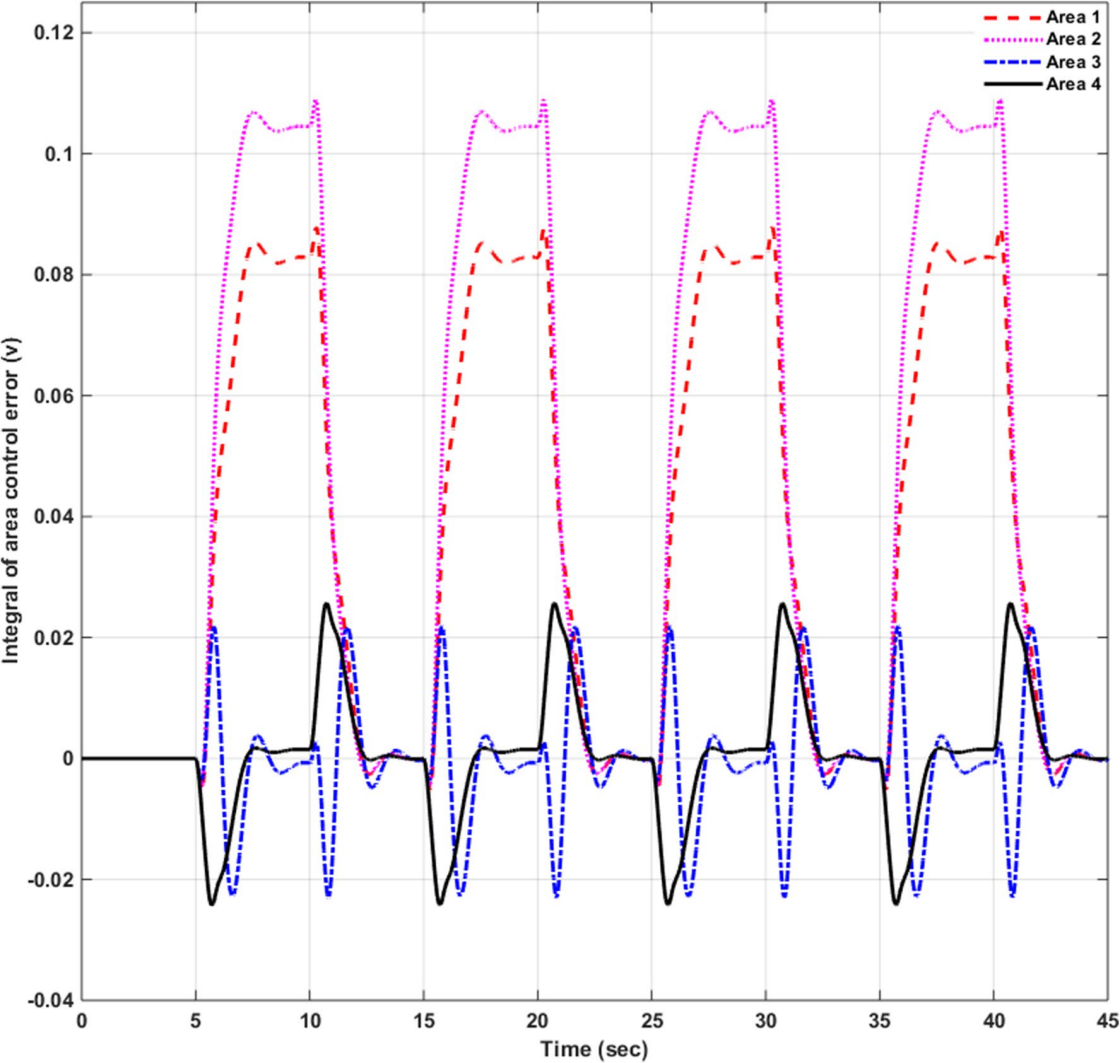
The integral of Area control errors for OQAGC with disturbances.

Finally, Fig. 10 shows the plots of the integral of area control errors (IACE1, IACE2, IACE3 and IACE4) in the presence of load disturbances. It can be observed that IACE4 has the fastest disturbance rejection (robustness), while IACE1, IACE2 and IACE3 approximately have the same disturbance rejection.

The parameter settings for the two cases are summarized in Table 3. Table 4 shows the comparison of the frequency deviation for two cases with, and without disturbances and with area control errors. The results illustrate that the OQAGC controller provides good dynamic performance in terms of rising time, settling time, and overshoot. The controller also shows good disturbance rejection time response.

**Table 3:** Parameters settings for the two simulations cases.

Case	$\Delta {f}_{i}$	${\Delta \mathrm{P}}_{\mathrm{tieij}}$	${AEC}_{i}$	${\Delta P}_{Di}$	${IAEC}_{i}$	${\Delta \mathrm{P}}_{\mathrm{T}}$
1	$0.0 \; \mathrm{ Hz}$	$0\;\mathrm{ Pu}$	0	0.01 pu	0	0
2	$0\;\mathrm{ Hz}$	$0\;\mathrm{Pu}$	0	0.01 pu	0	0

**Table 4:** Performance of OQAGC controller without and with disturbances.

Controller	Frequency deviations, Tie-line power deviations and Integral of area control errors									
	Area	Case 1					Case 2			
		State variables	Overshoot	Undershoot	Peak time (s)	Settling time (s)	Overshoot	Undershoot	Peak time (s)	Disturbance rejection time response (s)
OQAGC-controller-without and with disturbances	1	$\Delta {f}_{1}$	0.027	− 0.024	0.425	8	0.028	− 0.0253	0.75	4.75
	2	$\Delta {f}_{2}$	0.031	− 0.028	0.325	8	0.31	− 0.028	0.7	4.3
	3	$\Delta {f}_{3}$	0.04	− 0.042	0.5	8	0.038	− 0.042	0.7	4.35
	4	$\Delta {f}_{4}$	0.043	− 0.036	0.6	8	0.043	− 0.036	0.72	4 0.36
	1	${\Delta \mathrm{P}}_{\mathrm{tie}12}$	0.01	− 0.0026	0.3	7.7	0.026	− 0.0263	0.425	4.6
	2	${\Delta \mathrm{P}}_{\mathrm{tie}23}$	0.026	− 0.006	0.9	7.7	0.026	− 0.006	1.15	4.35
	3	${\Delta \mathrm{P}}_{\mathrm{tie}34}$	0.0024	− 0.022	0.275	7.5	0.022	− 0.04	0.43	4.4
	4	${\Delta \mathrm{P}}_{\mathrm{tie}41}$	0.0015	− 0.025	0.8	7.5	0.022	− 0.033	0.58	4.4
	1	${IACE}_{1}$	0.085	− 0.005	0.625	7	0.0085	− 0.005	2.55	8.32
	2	${IACE}_{2}$	0.0107	− 0.0046	0.725	7	1.07	− 0.004	2.4	8.32
	3	${IACE}_{3}$	0.022	− 0.023	0.575	8.8	0.022	− 0.0035	0.8	4.5
	4	${IACE}_{4}$	0.0022	− 0.024	0.02	6.8	0.022	− 0.0243	2.75	4.1

The performance of discrete OQAGC is compared with the hybrid neuro-fuzzy (ANFIS) presented by Prakash and Sinha^56^, 2014 as shown in Tables 5 and 6. Both controllers are imposed with 1% load disturbance at $t=0$ second (case1) for four area interconnected power system. The simulation results shown in Tables 1 and 2 show that discrete OQAGC is better in terms of peak undershoot and settling time and provides superior performance compared to ANFIS controller. The main disadvantage of the discrete OQAGC control structure is that the frequency deviations, tie-line deviations, and the integral of area control errors for the four areas are associated significantly with overshoot and undershot around the zero during the transient period. In addition, the system model grows and expands as the number of areas increases. This poses computational and design challenges.

**Table 5:** Comparative study of under peak overshoot^56^.

Controllers	$\Delta f$ area 1	$\Delta f$ area 2	$\Delta f$ area 3	$\Delta f$ area 4	$\Delta {P}_{tie}$( thermal-thermal)	$\Delta {P}_{tie}$( thermal-hydro)
ANFIS	− 0.061	− 0.061	− 0.054	− 0.054	− 0.052	− 0.054
OQAGC	− *0.024*	− *0.028*	− *0.042*	− *0.036*	− 0.0026	− 0.022

The designed Optimal Quadratic Automatic Generation Controller with peak overshoot values are in italics.

**Table 6:** Comparative study of settling time^56^.

Controllers	$\Delta f$ area 1	$\Delta f$ area 2	$\Delta f$ area 3	$\Delta f$ area 4	$\Delta {P}_{tie}$(thermal-thermal)	$\Delta {P}_{tie}$(thermal-hydro)
ANFIS	18	18	17	17	15	27
OQAGC	*8*	8	8	8	7.7	7.7

The designed Optimal Quadratic Automatic Generation Controller settling time values are in italics.

### Sensitivity analysis

The values of system parameters often change owing to environmental influences, equipment component replacement, changes in operating circumstances, and system component aging. The system's performance may suffer and it may become unstable^18^. As a result, sensitivity analysis is carried out to establish the resilience of the discrete OQAGC controller gains at nominal levels across large variations in operating load condition. The developed controller is compared with the Fuzzy gain scheduling controllers from literature in order to study the sensitivity analysis using the identical operating conditions for both controllers. The operational load condition and tie-line synchronization coefficient are adjusted from their nominal values in Table 1 by 25% and 50% of the nominal 1 percent size of step load perturbation (SLP) in changing step size of 25%, as shown in Table 7.

**Table 7:** Variations in the four-area interconnected power system's specified parameters.

Parameters variations rate	Variation of the tie-line synchronizing coefficient	Variations Load operating condition
Nominal	0.0866	1%
− 25% of nominal	0.06495	0.0075
− 50% of nominal	0.0433	0.005
+ 25% of nominal	0.10825	0.0125
+ 50% of nominal	0.1299	0.015

**Figure 11:**
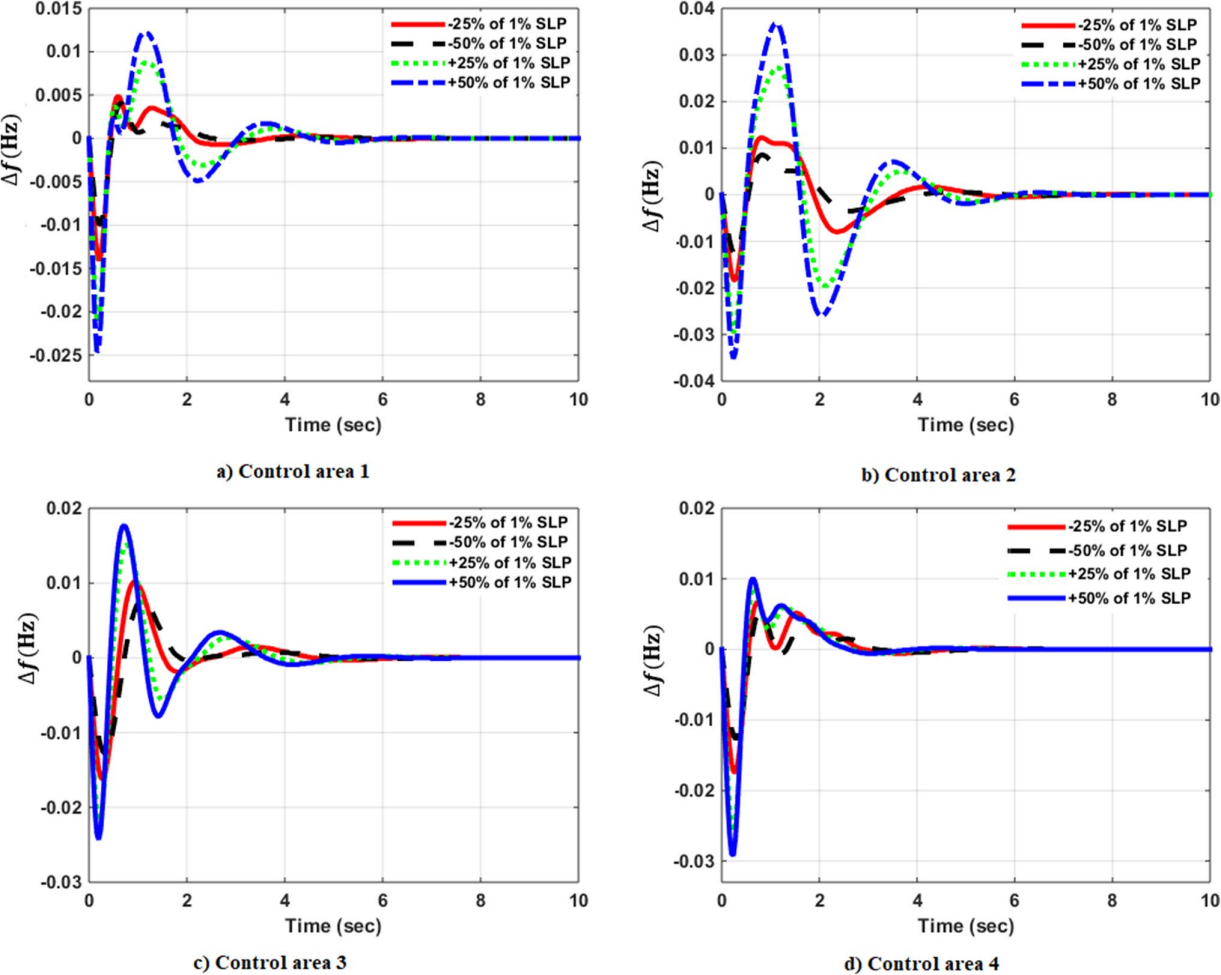
Dynamic response of Frequency deviations with (**a**) non-reheat turbine, (**b**) reheat turbine, (**c**) hydro turbine, (**d**) gas turbine when changes in % SLP applied at $\mathrm{t}= 0$ sec.

**Figure 12:**
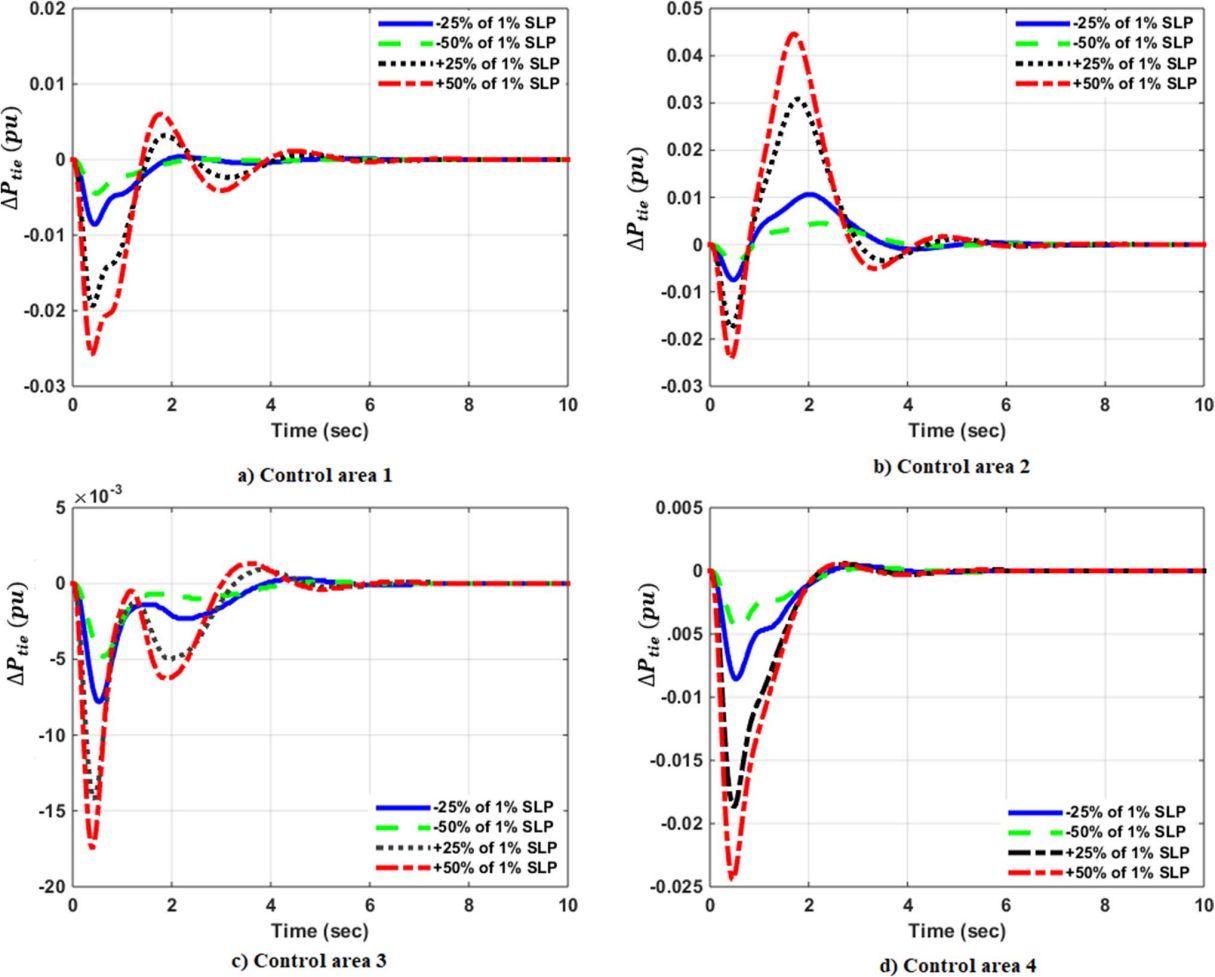
Dynamic responses of Tie-lie power deviations with (**a**) non-reheat turbine, (**b**) reheat turbine, (**c**) hydro turbine, (**d**) gas turbine when changes in % SLP applied at t = 0 sec.

Figures 11 and 12, show the simulated responses to frequency deviations and tie line power fluctuations from the sensitivity study. Figure 11 depicts the dynamic response of frequency deviations when 25% and 50% variations in nominal 1 percent magnitude of step load perturbation (SLP) in percent SLP are applied at t = 0 s. The results clearly reveal that all dynamic responses of frequency (Fig. 11) and tie-lie deviations (Fig. 12) have a similar pattern of peak of undershoot, peak of overshoot, and all have a settling period of 4 secs with small differences across areas. The settling time of the reaction time of reheat, hydro, and gas power plants, on the other hand, is almost the same, although the peak overshoot reactions varied slightly. During the transient period, however, all reaction times are connected to oscillations, with the exception of the reheat turbine plant's high peak overshoot response. Interaction factors between the subsystems produce variations in the peak overshot response, which must be adjusted for.

A close examination of all findings indicates that they are almost identical and have not varied much from the nominal values. As a result, the discrete OQAGC controller is resistant to fluctuations in load operating circumstances. The results of sensitivity analysis of discrete OQAGC is compared with Fuzzy gain scheduling controllers (Arya and Kumar^57^). The comparison is investigated for the highest overshoot and setting time for the frequency deviation and tie line deviation of thermal power systems (Figs. 11 and 12a). The simulation results in Table 8 reveal that Fuzzy gain scheduling controller has superior dynamic responses in terms of oscillations, peak of overshoot, and settling time compared to discrete OQAGC controller. For discrete OQAGC, the variations in peak overshot response during the transient period are produced by interaction variables across control regions, which must be adjusted for in the future by developing a discrete decentralized OQAGC controller.

**Table 8:** Comparative study of overshoot and settling time for sensitivity analysis^57^.

Controller	${\Delta f}_{1}$( reheat thermal system)		${\Delta P}_{tie12}$ between thermal -thermal	
	Settling time (s)	Peak overshoot	Settling time (s)	Peak overshoot
Fuzzy gain scheduling controller	3	− 0.08	4	− 0.008
OQAGC	5	− 0.025	6.5	− 0.027

.

### Multi-area power system model with renewable energy sources

**Figure 13:**
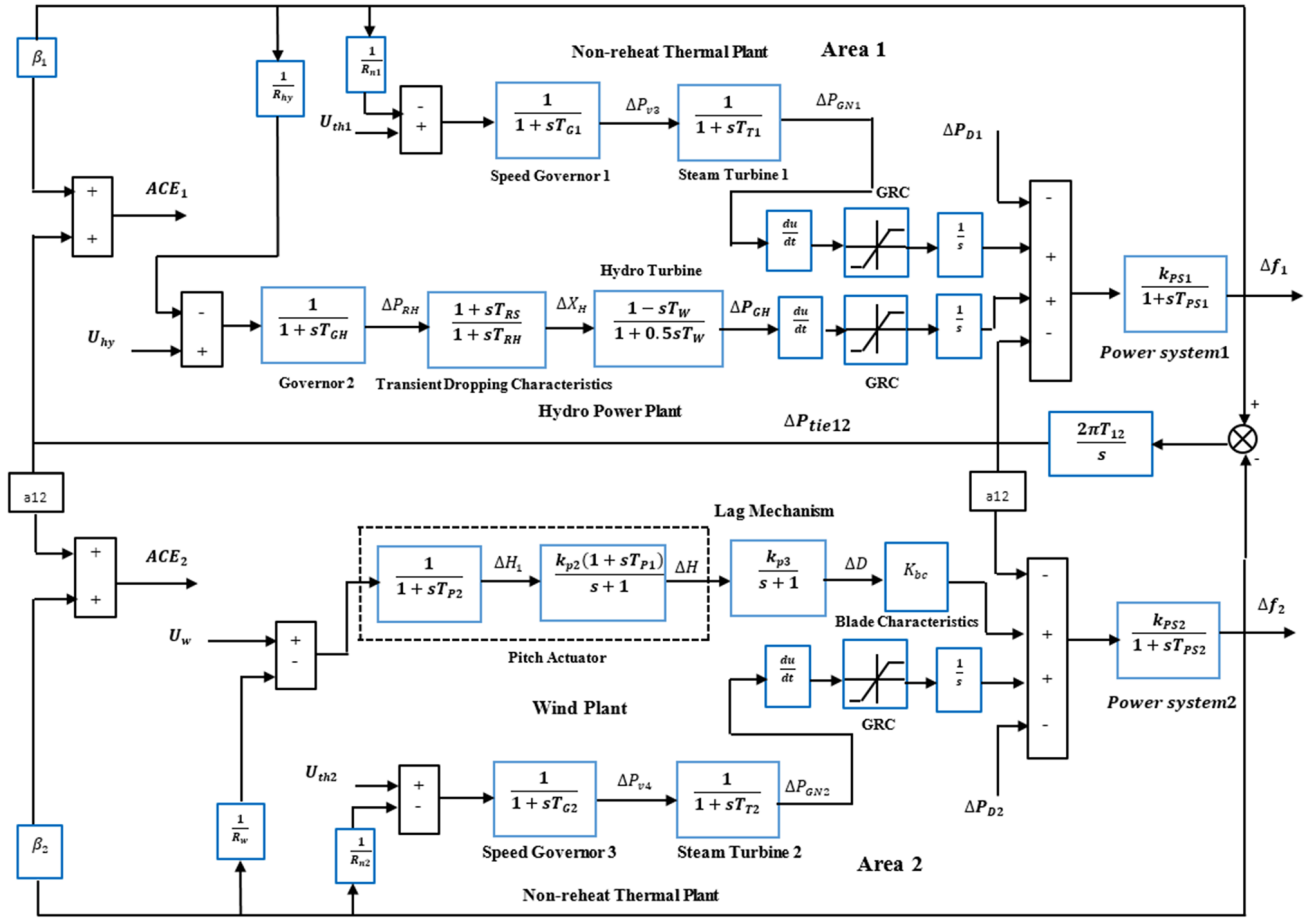
Transfer function model of multi-source power system.

To demonstrate the ability of the proposed OQAGC controller, the study is further extended to a multi-area multi-source interconnected power system with renewables as depicted in Fig. 13. Area 1 comprises non-reheat thermal and hydro plants while area 2 comprises a wind power plant and non-reheat thermal plant. The linear model of a wind power plant includes the transfer function for the pitch actuator, the transfer function for the lag mechanism that matches the phase/gain characteristic of the model, and a blade characteristics block^58^^.^ It is crucial to include the important inherent requirements and the basic physical constraints in the model in order to get an accurate understanding of the AGC problem ^43,45,59,60^. The important constraints which affect the power system performance are boiler dynamics, Generation Rate Constraint (GRC), and Governor Dead Band (GDB). In this study, the GRC was integrated into the system model as illustrated in Fig. 13 in order to test whether the proposed controller could be realistically implemented. It is assumed that the participation factors for thermal and hydro plants^33^ are 0.543470 and 0.3226034, respectively, and that the participation factor for wind turbine^55^ is 0.125.

#### State space equation of a multi-source power system with renewables

The procedure discussed in “Dynamics of a four area power system” section is applied here to obtain the state space equation of a multi-source power system with renewable shown in Fig. 13. The typical parameters of non-reheat thermals and Hydro plants are taken from Table 1, while wind turbine parameters are adopted from the work conducted by Sahu et al.^59^, where ${T}_{p1}=6$; ${T}_{p2}=0.04$, ${k}_{p2}=1.25$, ${k}_{p3}=1.4,$ ${T}_{g2}=0.08$, ${k}_{bc}=08$ ${R}_{w}=2.4$ and ${\beta }_{2}=0.425$. The system in Fig. 13 has 15 states variables where ${x}_{1}=\Delta {f}_{1}$, ${x}_{2}=\Delta {P}_{GN1}$, ${x}_{3}=\Delta {P}_{v3},$ ${x}_{4}={IACE}_{1},$ ${x}_{5}=\Delta {P}_{GH}$, ${x}_{6}=\Delta {X}_{H},$ ${x}_{7}=\Delta {P}_{RH,}$, ${x}_{8}=\Delta {P}_{tie12}$. ${x}_{9}=\Delta {f}_{2},$ ${x}_{10}=\Delta D,$ ${x}_{11}=\Delta H$, ${x}_{12}=\Delta {H}_{1}$, ${x}_{13}={IACE}_{2},$ ${x}_{14}=\Delta {P}_{GN2},$ ${x}_{15}=\Delta {P}_{v4}$. As result the state, control input and disturbance vectors of a linear state-space model for a two area multi-source power systems can be described as follows:

51 $$\begin{aligned} {x}^{T} & =[[\Delta {f}_{1} \Delta {P}_{GN1} \Delta {P}_{v3} {IACE}_{1} {\Delta P}_{GH} \Delta {X}_{H} \Delta {P}_{RH} \Delta {P}_{tie12} {IACE}_{2} {\Delta P}_{tie12} \Delta {f}_{2} \Delta D \Delta H {\Delta H}_{1} {IACE}_{2} {\Delta P}_{GN2} \Delta {P}_{v4}{]}^{T} \\ {u}^{T} & ={\left[{U}_{th1} {U}_{hy} { U}_{w} {U}_{th2}\right]}^{T} \\ {w}^{T} & ={\left[{\Delta P}_{D1}\boldsymbol{ }\boldsymbol{ }\boldsymbol{ }\boldsymbol{ }{\Delta P}_{D2}\right]}^{{\varvec{T}}} \\ {C}^{T} & ={\left[\boldsymbol{ }{\Delta f}_{1}\boldsymbol{ }\boldsymbol{ }\boldsymbol{ }{\Delta f}_{2} { \Delta P}_{tie12} \right]}^{{\varvec{T}}} \end{aligned}$$

The continuous the continuous state matrix ${A}_{m}$, the input matrix ${B}_{m},$ and disturbance matrix ${\Gamma }_{m}$ are given below:

52 $$\begin{aligned} {A}_{m} & =\left[\begin{array}{ccccccccccccccc}-\frac{1}{{T}_{ps1}}& \frac{{k}_{ps1}}{{T}_{ps1}}& 0& 0& \frac{{k}_{ps1}}{{T}_{ps1}}& 0& 0& -\frac{{k}_{ps1}}{{T}_{ps1}}& 0& 0& 0& 0& 0& 0& 0\\ 0& -\frac{1}{{T}_{T1}}& \frac{1}{{T}_{T1}}& 0& 0& 0& 0& 0& 0& 0& 0& 0& 0& 0& 0\\ -\frac{1}{{R}_{n1}{T}_{G1}}& 0& -\frac{1}{{T}_{G1}}& 0& 0& 0& 0& 0& 0& 0& 0& 0& 0& 0& 0\\ {\beta }_{1}& 0& 0& 0& 0& 0& 0& 1& 0& 0& 0& 0& 0& 0& 0\\ -\frac{{2T}_{RS}}{{T}_{RH}{T}_{GH}{R}_{hy}}& 0& 0& 0& -\frac{2}{{T}_{W}}& \frac{2}{{T}_{W}}-\frac{2}{{T}_{RH}}& \frac{2}{{T}_{RH}}-\frac{2{T}_{RS}}{{T}_{RH}{T}_{GH}}& 0& 0& 0& 0& 0& 0& 0& 0\\ -\frac{{T}_{RS}}{{T}_{RH}{T}_{GH}{R}_{hy}}& 0& 0& 0& 0& -\frac{1}{{T}_{RH}}& \frac{1}{{T}_{RH}}-\frac{{T}_{RS}}{{T}_{RH}{T}_{GH}}& 0& 0& 0& 0& 0& 0& 0& 0\\ -\frac{1}{{T}_{GH}{R}_{hy}}& 0& 0& 0& 0& 0& -\frac{1}{{T}_{GH}}& 0& 0& 0& 0& 0& 0& 0& 0\\ 2\pi {T}_{12}& 0& 0& 0& 0& 0& 9& -2\pi {T}_{12}& 0& 0& 0& 0& 0& 0& 0\\ 0& 0& 0& 0& 0& 0& 0& \frac{{k}_{ps2}}{{T}_{ps2}}& -\frac{1}{{T}_{ps2}}& \frac{{k}_{ps2}{k}_{bc}}{{T}_{ps2}}& 0& 0& 0& \frac{{k}_{ps2}}{{T}_{ps2}}& 0\\ 0& 0& 0& 0& 0& 0& 0& 0& 0& -1& {k}_{3}& 0& 0& 0& 0\\ 0& 0& 0& 0& 0& 0& 0& 0& 0& -\frac{{k}_{p2 }{T}_{p1}}{{R}_{w}{T}_{p2}}& 0& -1& {k}_{p2}-\frac{{k}_{p2}{T}_{p1}}{{T}_{p2}}& 0& 0\\ 0& 0& 0& 0& 0& 0& 0& 0& -\frac{1}{{{R}_{w}T}_{p2}}& 0& 0& -\frac{1}{{T}_{p2}}& 0& 0& 0\\ 0& 0& 0& 0& 0& 0& 0& -1& {\beta }_{2}& 0& 0& 0& 0& 0& 0\\ 0& 0& 0& 0& 0& 0& 0& 0& 0& 0& 0& 0& 0& -\frac{1}{{T}_{T2}}& \frac{1}{{T}_{t2}}\\ 0& 0& 0& 0& 0& 0& 0& 0& -\frac{1}{{R}_{n2}{T}_{G2}}& 0& 0& 0& 0& 0& -\frac{1}{{T}_{G2}}\end{array}\right] \\ {B}_{m}^{T} & =\left[\begin{array}{ccccccccccccccc}0& 0& \frac{0.54347}{{T}_{G1}}& 0& 0& 0& 0& 0& 0& 0& 0& 0& 0& 0& 0\\ 0& 0& 0& 0& \frac{0.3260842{T}_{RS}}{{T}_{RH}{T}_{GH}}& \frac{0.3226084*{T}_{RS}}{{T}_{RH}{T}_{GH}}& \frac{0.322684}{{T}_{GH}}& 0& 0& 0& 0& 0& 0& 0& 0\\ 0& 0& 0& 0& 0& 0& 0& 0& 0& 0& \frac{{0.125 T}_{p1}{k}_{p2}}{{T}_{p2}}& \frac{0.125}{{T}_{p2}}& 0& 0& 0\\ 0& 0& 0& 0& 0& 0& 0& 0& 0& 0& 0& 0& 0& 0& \frac{0.4344705}{{T}_{G2}}\end{array}\right] \\ {\Gamma }_{m}^{T}& =\left[\begin{array}{ccccccccccccccc}-\frac{{k}_{ps1}}{{T}_{ps1}}& 0& 0& 0& 0& 0& 0& 0& 0& 0& 0& 0& 0& 0& 0\\ 0& 0& 0& 0& 0& 0& 0& 0& -\frac{{k}_{ps1}}{{T}_{ps1}}& 0& 0& 0& 0& 0& 0\end{array}\right] \end{aligned}$$

#### Simulation results of multi-source interconnected power system

the design procedures described in “The design of OQAGC control” and “A functional minimization method” sections are applied to the Multi-Area Power system model with renewable energy sources as shown in Fig. 13. Based on the functional minimization method (FFM) in “A functional minimization method” section, Eqs. (18)–(31), the state and control weighting matrices ${Q}_{m}$ and ${R}_{m}$ were formulated for a multi-area power system model with renewable energy sources. The excursions ${ACE}_{i}$, ${IACE}_{i}$ and control input u are substituted into Eq. (18). As a result, the cost function $J$ for the systems can be written as:

53 $$J=\frac{1}{2}\sum_{k={k}_{0}}^{\infty }\left[{{{{B}_{1}}^{2}x}_{1}}^{2}+2{\beta }_{1}{x}_{1}{x}_{8}+{{x}_{8}}^{2}\right]+\left[{{{{B}_{2}}^{2}x}_{9}}^{2}-2{\beta }_{1}{x}_{9}{x}_{8}+{{x}_{8}}^{2}\right]+{\left({x}_{4}\right)}^{2}+{\left({x}_{13}\right)}^{2}+\alpha [{{U}_{th1}}^{2}+{{U}_{hy}}^{2}+{{U}_{w}}^{2}+{{U}_{th2}}^{2}]$$

where $\alpha$ is the vector of participation factors, ${U}_{th1}$, ${U}_{hy}$, ${U}_{w}$ and ${U}_{th2}$ are control signals applied to non-reheat thermal 1, hydro, wind turbine , and non-reheat thermal 2 plants respectively.

The partial differential state and control equations: (24–31) were used and organized to derive the state weighting matrix ${Q}_{m}$ and control weighting matrix. The numerical values for weighting matrices ${Q}_{m}$ and ${R}_{m}$ are found as follows:

54 $${{\varvec{Q}}}_{{\varvec{m}}}=\left[\begin{array}{ccccccccccccccc}0.1806& 0& 0& 0& 0& 0& 0& 0.4250& 0& 0& 0& 0& 0& 0& 0\\ 0& 0& 0& 0& 0& 0& 0& 0& 0& 0& 0& 0& 0& 0& 0\\ 0& 0& 0& 0& 0& 0& 0& 0& 0& 0& 0& 0& 0& 0& 0\\ 0& 0& 0& 1& 0& 0& 0& 0& 0& 0& 0& 0& 0& 0& 0\\ 0& 0& 0& 0& 0& 0& 0& 0& 0& 0& 0& 0& 0& 0& 0\\ 0& 0& 0& 0& 0& 0& 0& 0& 0& 0& 0& 0& 0& 0& 0\\ 0& 0& 0& 0& 0& 0& 0& 0& 0& 0& 0& 0& 0& 0& 0\\ 0.4250& 0& 0& 0& 0& 0& 9& 2.0000& -0.4250& 0& 0& 0& 0& 0& 0\\ 0& 0& 0& 0& 0& 0& 0& -0.4250& 0.1806& 0& 0& 0& 0& 0& 0\\ 0& 0& 0& 0& 0& 0& 0& 0& 0& 0& 0& 0& 0& 0& 0\\ 0& 0& 0& 0& 0& 0& 0& 0& 0& 0& 0& 0& 0& 0& 0\\ 0& 0& 0& 0& 0& 0& 0& 0& 0& 0& 0& 0& 0& 0& 0\\ 0& 0& 0& 0& 0& 0& 0& 0& 0& 0& 0& 0& 1& 0& 0\\ 0& 0& 0& 0& 0& 0& 0& 0& 0& 0& 0& 0& 0& 0& 0\\ 0& 0& 0& 0& 0& 0& 0& 0& 0& 0& 0& 0& 0& 0& 0\end{array}\right], \quad {{\varvec{R}}}_{{\varvec{m}}}=\left[\begin{array}{cccc}0.5435& 0& 0& 0\\ 0& 0.3226& 0& 0\\ 0& 0& 0.1250& 0\\ 0& 0& 0& 0.5444\end{array}\right]$$

According to Eq. (15), the numerical values of optimal feedback gains matrix is found as follows:

55 $${L}_{m}=\left[\begin{array}{ccccccccccccccc}0.3737& 0.8423& 0.2264& 1.2059& 1.3067& 1.2566& -0.5067& -0.2141& -0.0358& -0.1855& -0.0506& 0.3073 & -0.1243& -0.1521& -0.0398\\ 0.2297& 0.3636 & 0.0877& 0.4660& 0.4872& 0.8368& -0.2258& -0.0120& -0.0804& -0.1183 & -0.0172& 0.0843& -0.0753& -0.1175 & -0.0252\\ -0.1754& -0.4655& -0.1187& 0.1967& -0.5828 & -0.0179& 0.0939& 1.5877& -0.3347& 2.3510& 0.5688& -4.1123& 1.6726& 1.9994& 0.5394\\ -0.0461& -0.1506& -0.0389& 0.1472& -0.1878& -0.0138& 0.0315& 0.5995& 0.0192& 0.8870& 0.2752& -1.7271& 0.6529& 0.7322& 0.2111\end{array}\right]$$

**Figure 14:**
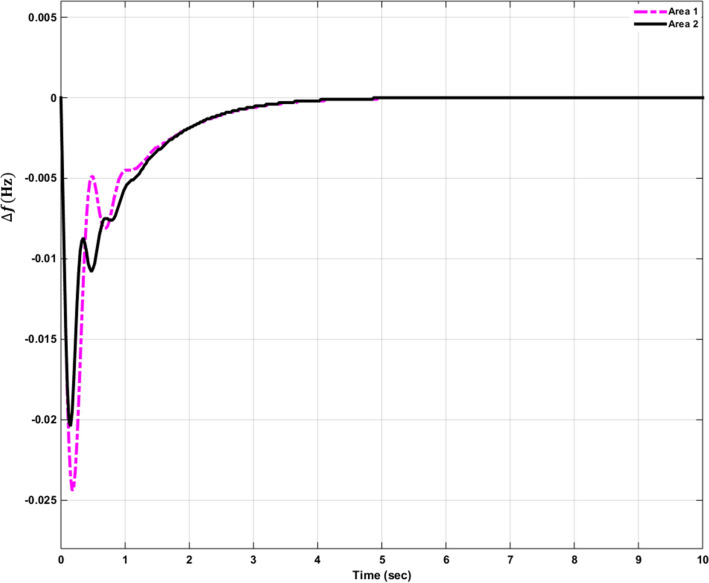
Frequency deviations for Areas 1 and 2.

**Figure 15:**
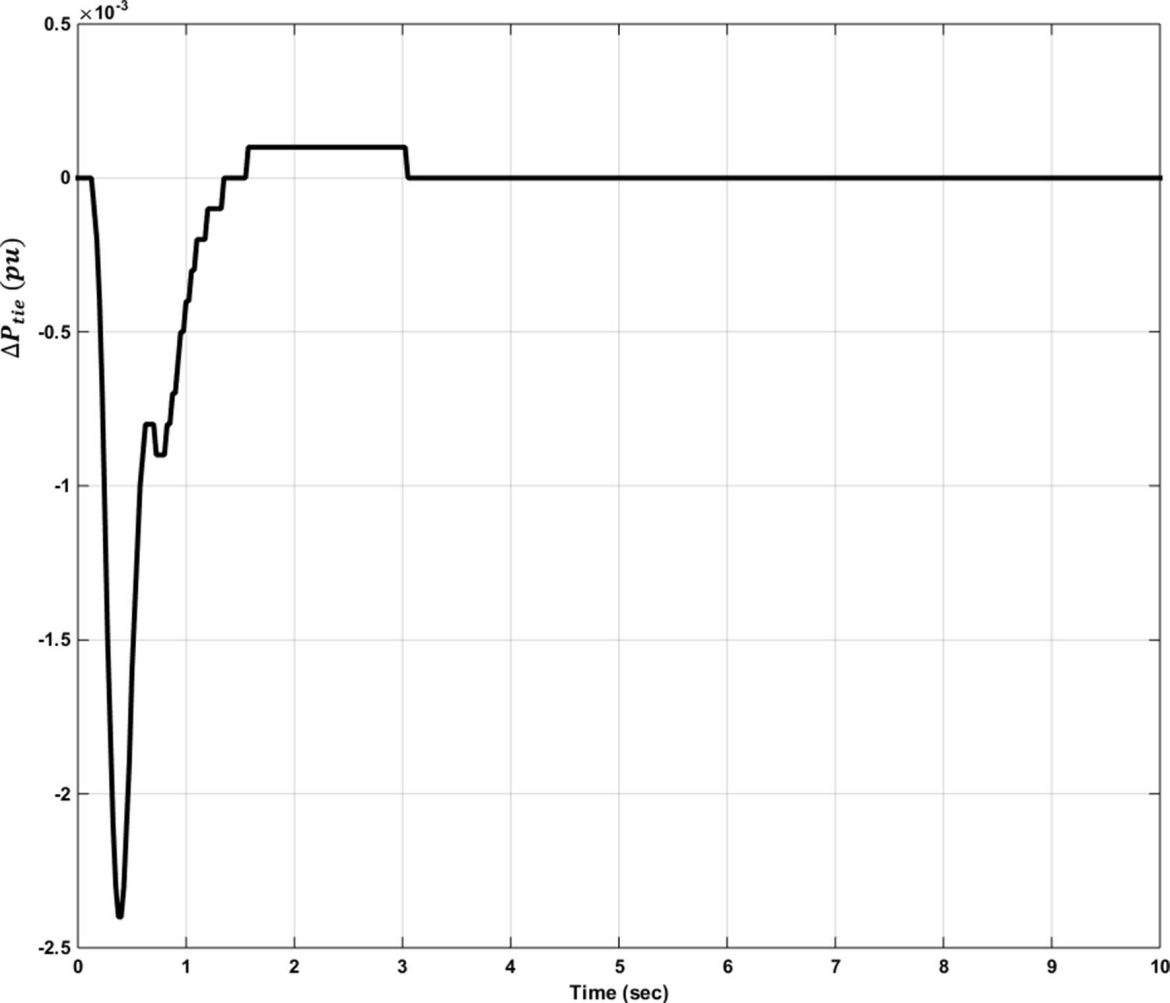
Tie-line power deviation between areas 1 and 2.

**Figure 16:**
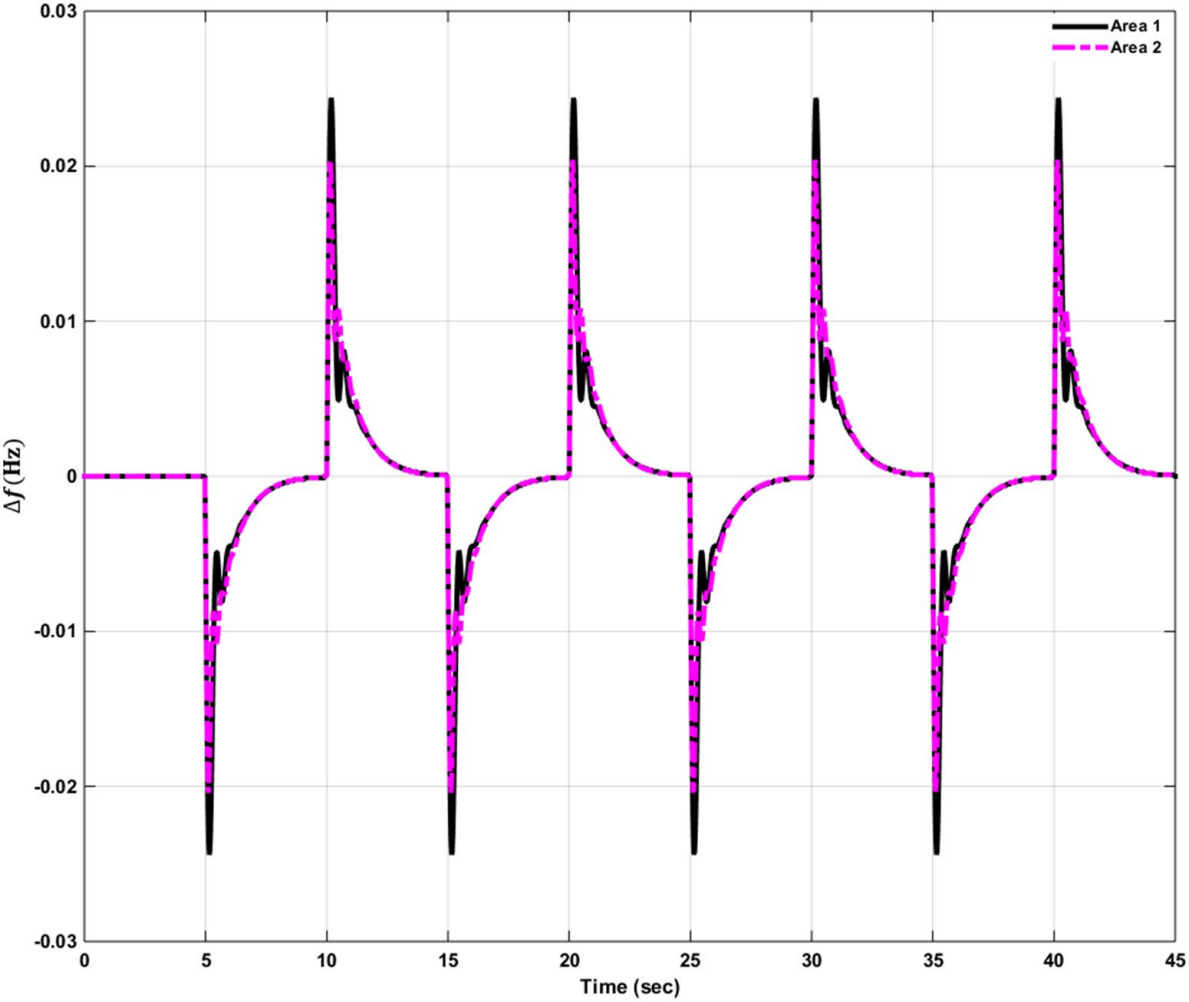
Frequency deviation results of two areas 1 and 2 with simultaneous SLPs.

**Figure 17:**
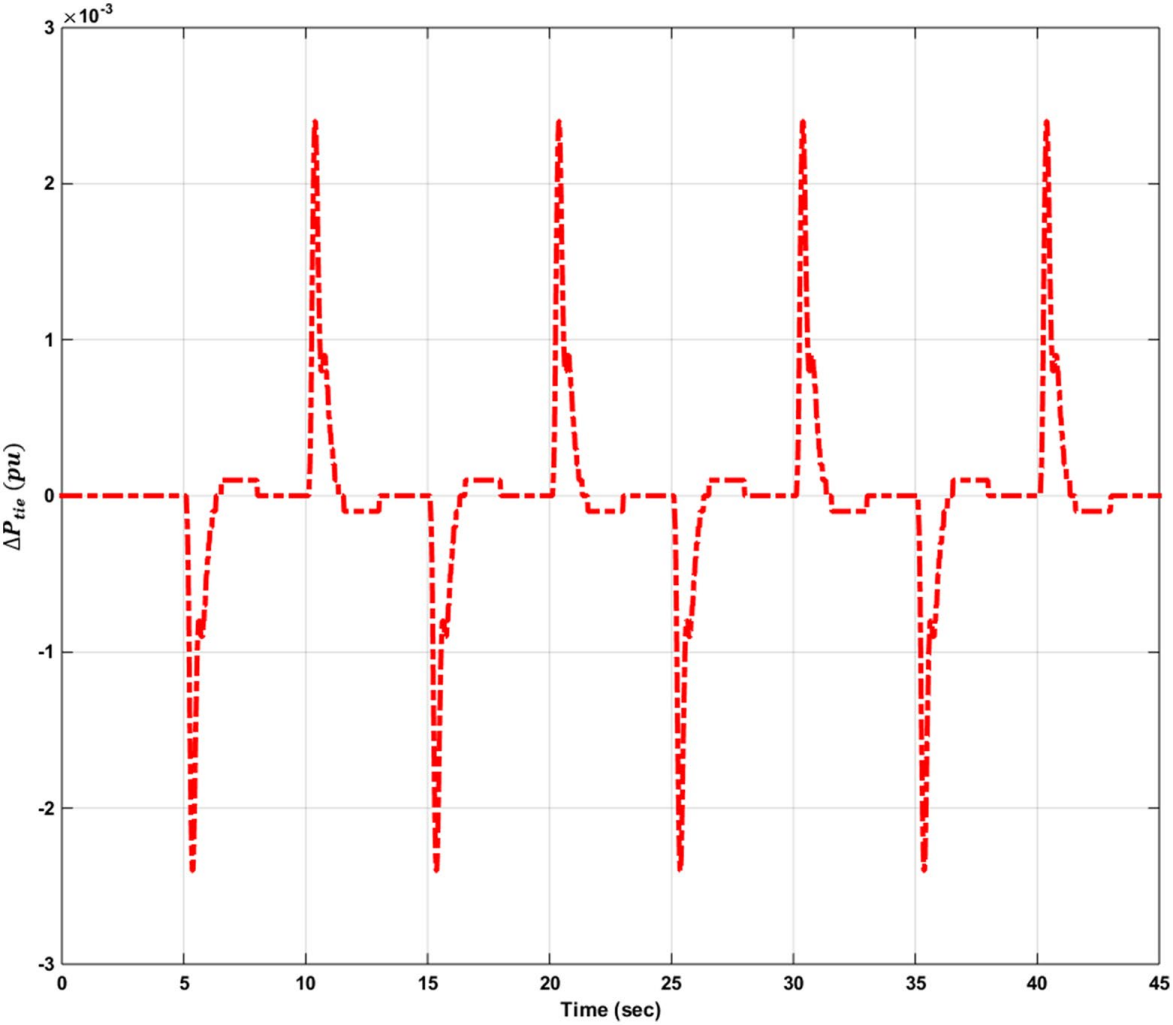
Tie-line power deviation result in both areas with simultaneous SLPs.

The computer simulations were conducted with OQAGC controllers’ gains for two different cases. First, the simulation responses of the closed-loop systems are obtained by setting the initial values of the step load perturbation (SLP) at 0.01 pu in area 1, while keeping the rest of the initial values to zeros. Secondly, the simulation results of the closed loop are obtained for 1% SLP which are added simultaneously to each area while the rest of the initial variables are set to zero. The simulation results are presented in Figs. 14, 15, 16, 17 and detailed explanations of those simulation results are presented below.

Figures 14, 15 show simulation results for frequency deviations and Tie-line power deviations. These are the results obtained when the step load perturbation (SLP) set at 0.01 pu in area1 at the initial time $t=0$ while keeping the rest of the initial values to zeros.

Simulation results for frequency deviations, and Tie-line power deviations are shown in Figs. 16 and 17 respectively.

The simulations result for frequency deviations and tie-lie power deviations of both areas are summarized in Tables 9 and 10, respectively. It is observed that the OQAGC controller achieves almost the same frequency deviation responses of peak overshoots when the Step Load Perturbations are applied respectively ( SLP $=0.01\mathrm{pu}$) at t $=0 sec$ and simultaneously at various time intervals.. The area 2 has 18% difference of peak overshoot compared to that of area1 for both cases. The settling time of area 2 is smaller than the settling time of area 1 with zero steady state errors in both cases. The settling time and peak overshoot are same for the tie-line power deviations. The tie line deviation has lesser disturbance rejection time response when compared to disturbance rejection time response of the frequency deviations.

**Table 9:** Performance of COQAGC in terms of peak overshoot (POS,) settling time and disturbance rejection response time (DRRT).

Frequency deviations					
Type of controller	Area	Settling time	Peak OS	DRRT	Steady state error
OQAGC controller with $SLP=0.01 p.u at t=0$	1	4.95 s	− 0.0244	–	0
	2	4.875 s	− 0.0201	–	0
Difference %	–	2%	18%	–	0%
DOQAGC controller with simultaneous $SLP=0.01p.u$	1	4.95 s	− 0.0244	4.85 s	0
	2	4.875 s	− 0.0201	4.925 s	0
Difference %	-	2%	18%	2%	0%

**Table 10:** Performance of COQAGC in terms of POS, ST and DRRT with simultaneous disturbances.

Tie-line power deviations (pu)					
Type of controller	Between areas	Settling time (s)	Peak OS	DRRT	Steady state error
DOQAGC controller with $SLP=0.1p.u \; at \; t=0$	1 and 2	3.05	− 0.0024	–	0
DOQAGC controller with simultaneous $SLP=0.1p.u$	1 and 2	3.05	− 0.0024	3.05 s	0

#### Generation rate constraint

**Figure 18:**
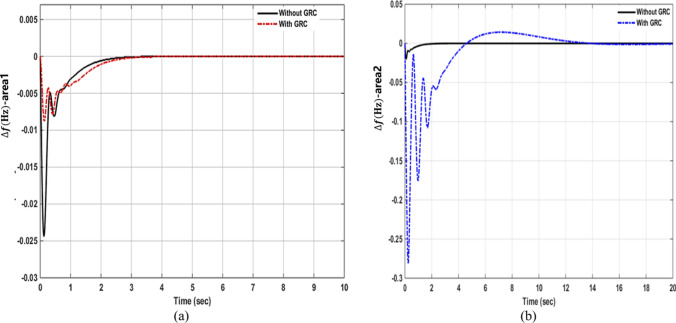
Frequency deviation response to 1% SLP in multi-source power system with and without GRC for thermal plant (**a**) Area 1, (**b**) Area 2.

**Figure 19:**
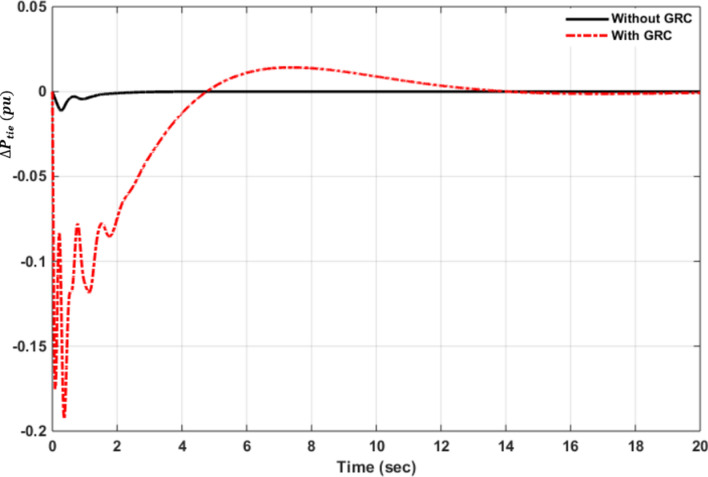
Tie-line deviation response to 1% SLP in multi-source power system with and without GRC.

An additional test of the effectiveness of the OQAGC controller is conducted by considering the GRC of thermal and hydroelectric plants in the multi-source power system model discussed above. This study considers a GRC of 10%/min (0.0017 pu/s) for a single non-reheat thermal plant and a GRC for the hydro plant of 270%/min (+ 0.045pu/s) for raising a generation, and 360%/min (– 0.06pu/s) for a lowering generation for the hydro plant. Simulations are conducted for non-reheat and hydro-generating plants with and without the GRC with limits described above. The effect of GRC on the frequency deviation responses of multi-source power system obtained with OQAGC controller at 1% step load perturbation are shown in Figs. 18 and 19 respectively. These results show that the OQAGC controller exhibits a larger peak overshoot and a longer settling time when using GRC. However, in these particular two cases, the dynamic of the multi-source power system with GRC meets the automatic generation control requirements as described in the literature by Parmar^43^. It also can be seen that control area 2 is more affected by generation rate constraints than control area 1 in terms of longer settling time and oscillations due to wind plant characteristics.

It has been observed that the dynamic performance of the system deteriorates if GRC limit are applied in existence of wind plant. It is therefore necessary to take GRC into account when studying the system realistically. The results of GRC of discrete OQAGC is compared with optimal output control (Parmar^43^). The comparison is investigated for the highest overshoot and setting time for the frequency deviation (Fig. 18b). The simulation results in Table 11 reveal that the discrete OQAGC controller has superior dynamic responses in terms of peak of overshoot, and settling time compared to Optimal output control.

**Table 11:** Comparative study of overshoot with and without GRC for tie line power deviations^43^.

Controllers	Thermal power system	
	Settling time (s)	Peak overshoot
Optimal output control	25	− 0.091
OQAGC	16	− 0.25

## Conclusion

For systematically determining state and control weighting matrices for load frequency management in N regions of interconnected power systems with and without disturbances, a generalized functional minimization technique has been designed. Discrete OQAGC controllers for a four-area power system and two area multi source power system is developed using the optimum control theory and the Lagrangian conventional multipliers approach. The study took into account the model of a four-area power system with area control errors, and the integral of area control errors were added to the model's state vector to assure zero steady-state errors.

The simulation findings reveal the following:

- The discrete optimum quadratic AGC controllers based on functional cost reduction are more resistant to disturbance rejection.
- This work developed an OQAGC approach for minimizing the cost function by taking into account area control errors, the integral of area control errors, and energy expenditure control.
- The functional minimization approach is used to choose the state and control weighting matrices.
- The developed discrete OQAGC control and simulation results in this paper are based on discrete quadratic optimal control theory and their application can be used to solve the problem of complex large-scale power systems.
- The sensitivity study analysis prove that the developed discrete OQAGC controller is resistant to fluctuations in load operating circumstances.
- The GRC analysis prove that the developed discrete optimal AGC controller meets the requirements of the automatic generation problem.

## Data Availability

All data generated or analyzed during this study are included in this article and its supplementary information files are provided as well. References for all data are listed below: Reference 1: Title: A More Realistic Model of Centralized Automatic Generation Control in Real-time Environment https://www.tandfonline.com/doi/pdf/10.1080/15325008.2015.1076540?needAccess=true. Reference 2: Title: Load frequency control of a realistic power system with multi-source power generation https://reader.elsevier.com/reader/sd/pii/S0142061512001676?token=CAA77117B84E5194AAD76BFC2696949586B6035A8504929E4B92B1912DAEEA331AD69EB8557111E43ED07B06F5448ED4&originRegion=eu-west-1&originCreation=20220617205627.

## List of symbols

$\Delta {\mathrm{P}}_{\mathrm{tieij}}$: Tie-line power deviation
$\Delta {\mathrm{f}}_{\mathrm{i}}$: Frequency deviation for the ith area (Hz)
$\Delta {\mathrm{P}}_{\mathrm{Di}}$: The load input deviation for the ith area (pu.MW)
${\mathrm{IAEC}}_{\mathrm{i}}$: The ith area ‘s integral deviation of area control error
${\mathrm{T}}_{\mathrm{psi}}$: The time constant of the power system for the ith area (s)
${\mathrm{T}}_{\mathrm{ti}}$: The turbine time constant for the ith area (s)
${\mathrm{T}}_{\mathrm{Gi}}$: Governor time constant for the ith area (s)
${\mathrm{T}}_{\mathrm{ij}}$: The tie-line synchronizing coefficient between the ith area and the jth area (pu.MW)
${\mathrm{k}}_{\mathrm{psi}}$: The power system’s gain for the ith area
${\mathrm{ACE}}_{\mathrm{i}}$: Area control error for the ith area
${\mathrm{R}}_{\mathrm{nr}}={\mathrm{R}}_{\mathrm{th}}={\mathrm{R}}_{\mathrm{hy}} \; \mathrm{ and }\; {\mathrm{R}}_{\mathrm{g}}$: The governor speed regulation parameters of thermal, hydro, and gas power plants respectively (Hz.pu.${\mathrm{MW}}^{-1}$)
${\mathrm{B}}_{\mathrm{i}}$: The ith area’s frequency biasing factor
$\mathrm{x}$: The states of the overall interconnected power system
${\mathrm{k}}_{\mathrm{i}}$: The quadratic optimal control constant vector for the ith area
${\mathrm{u}}_{\mathrm{i}}$: The control law signal for the ith area
${\mathrm{P}}_{\mathrm{R}}$: The rated power
$\Delta {\updelta }_{\mathrm{ij}}$: The degree deviation between any two areas
N: Number of interconnected power systems
${\mathrm{T}}_{\mathrm{SG}}$: Speed governor time constant (s)
${\mathrm{k}}_{\mathrm{r}}$: Steam turbine reheat constant
${\mathrm{T}}_{\mathrm{r}}$: Steam turbine reheat time constant (s)
${\mathrm{T}}_{\mathrm{w}}$: The nominal starting time of water in penstock (s)
${\mathrm{T}}_{\mathrm{rs}}$: Time constant for resetting the hydro turbine speed governor (s)
${\mathrm{T}}_{\mathrm{rh}}$: Hydro turbine speed governor transient drop time constant (s)
${\mathrm{T}}_{\mathrm{gh}}$: Hydro turbine speed governor main servo time constant (s)
${\mathrm{X}}_{\mathrm{g}}$: The gas turbine speed governor’s lead time constant (s)
${\mathrm{Y}}_{\mathrm{g}}$: Valve positioner constant of the gas turbine (s)
${\mathrm{C}}_{\mathrm{g}}$: Gas turbine valve positioner
${\mathrm{b}}_{\mathrm{g}}$: Gas turbine constant of valve positioner (s)
${\mathrm{T}}_{\mathrm{f}}$: Time constant for gas turbine (s)
${\mathrm{T}}_{\mathrm{cr}}$: Gas turbine combustion reaction time delay (s)
${\mathrm{T}}_{\mathrm{cd}}$: Volume-time constant of gas turbine compressor discharge (s)
${\mathrm{\alpha }}_{\mathrm{n}}={\mathrm{\alpha }}_{\mathrm{th}}={\mathrm{\alpha }}_{\mathrm{hy}} \; \mathrm{ and } \; {\mathrm{\alpha }}_{\mathrm{g}}$: Thermals, hydro and gas generating units all have participating factors.
${a}_{1}$: Constant of valve positioner
GRC: Generation Rate Constraints
${\Delta P}_{n}$: Generation output power deviation of non-reheat thermal plant in pu. MW.
${\Delta P}_{v1}$: Non-reheat thermal plant governor valve position deviation pu.
${\Delta P}_{r}$: Generation output power deviation of reheat thermal plant in pu. MW.
${\Delta P}_{Rt}$: A deviation in the intermediate governor output of the reheat thermal plant in pu.
${\Delta P}_{v2}$: A deviation in the output of the steam turbine governor of the reheat thermal plant in Pu
${\Delta P}_{hy}$: Generation output power deviation of hydro plant in pu.MW
${\Delta X}_{h}$: Hydro plant governor valve position deviation in Pu
${\Delta P}_{rh}$: Governor valve servo motor position deviation of hydro plant in pu
${\Delta P}_{g}$: Generation output power deviation of gas plant in pu.MW
${\Delta P}_{fc}$: Deviation in intermediate state of fuel system and combustor of gas turbine in pu
${\Delta X}_{g}$: Deviation in intermediate state of gas turbine speed in pu

## References

[1] Shankar R, Pradhan SR, Chatterjee K, Mandal R (2017). A comprehensive state of the art literature survey on LFC mechanism for power system. Renew. Sustain. Energy Rev. J..

[2] Tungadio DH, Ramesh B, Mukwanga S (2018). Optimal control of active power of two micro-grids interconnected with two AC tie-lines. Electr. Power Compon. Syst..

[3] Alrifai MT, Hassan MF, Zribi M (2011). Decentralized load frequency controller for a multi-area interconnected power system. Int J. Electr. Power Energy Syst..

[4] Esmail M, Tzoneva R, Krishnamurthy S (2017). Review of automatic generation control in deregulated environment. IFAC-PapersOnline..

[5] Athay TM (1987). Generation scheduling and control. Proc. IEEE.

[6] Wood, A. J., Wollenberg, B.F & Sheblé, G.B. Power *Generation, Operation, and Control*, 3ed, Chapter 10, 468–500 (Wiley Inc, 2014).

[7] Tielens P, Van Hertem D (2016). The relevance of inertia in power systems. Renew. Sustain. Energy Rev..

[8] Masood, N. A., Modi, N. & Yan, R. Low inertia power systems: Frequency response challenges and a possible solution. In *Proceedings of the 2016 Australasian Universities Power Engineering Conference (AUPEC)*, (2016).

[9] Tielens, P. & van Hertem, D. Grid inertia and frequency control in power systems with high penetration of renewables. In *Proceedings of the Young Researcher’s Symposium in Electrical Power Engineering* (2012).

[10] Alhelou HH, Hamedani-Golshan ME, Zamani R, Heydarian-Forushani E, Siano P (2018). Challenges and opportunities of load frequency control in conventional, modern and future smart power systems: A comprehensive review. Energies.

[11] Cohn, N. Transactions of the american institute of electrical engineers. Part III: Power Apparatus and Systems **75** (3), (1956).

[12] Kumar IP, Kothari DP (2005). Recent philosophies of automatic generation control strategies in power systems. IEEE Trans. Power Syst..

[13] Delassi A, Arif S, Mokrani L (2015). Load frequency control problem in interconnected power systems using robust fractional PIλ D controller’. Ain Shams Eng. J..

[14] Tan W, Xu Z (2009). Robust analysis and design of load frequency controller for power systems. Electr. Power Syst. Res..

[15] Drouin M, Abou-Kandil H, Mariton M (1991). Control of Complex Systems Methods and Technology.

[16] Shayeghi H, Shayanfar HA, Jalili A (2009). Load frequency control strategies: A state-of-the-art survey for the researcher. Energy Convers. Manag..

[17] Ullah K, Basit A, Ullah Z, Aslam S, Herodotou H (2021). Automatic generation control strategies in conventional and modern power systems: A comprehensive overview. Energies.

[18] Debbarma, S., Saikia, L. C. & Sinha, N. Automatic generation control of multi-area system using two degree of freedom fractional order PID controller: A preliminary study. In *Proceedings of the 2013 IEEE PES nAsia-Pacific Power and Energy Engineering Conference (APPEEC), Kowloon, China, 8–11 December 1–6* (2013).

[19] Dash, P., Saikia, L. C. & Sinha, N. AGC of a multi-area interconnected system with FACTS and firefly optimized 2DOF PID controller. In *Proceedings of the 2014 International Conference on Control, Instrumentation, Energy and Communication (CIEC), Calcutta, India, 31 January*, 397–401 (2014).

[20] Naidu K, Mokhlis H, Bakar AA (2014). Multiobjective optimization using weighted sum artificial bee colony algorithm for load frequency control. Int. J. Electr. Power Energy Syst..

[21] Mohanty B, Panda S, Hota P (2014). Controller parameters tuning of differential evolution algorithm and its application to load frequency control of multi-source power system. Int. J. Electr. Power Energy Syst..

[22] Ali E, Abd-Elazim S (2013). BFOA based design of PID controller for two area Load Frequency Control with nonlinearities. Int. J. Electr. Power Energy Syst..

[23] Shiva CK, Mukherjee V (2015). Automatic generation control of interconnected power system for robust decentralized random load disturbances using a novel quasi-oppositional harmony search algorithm. Int. J. Electr. Power Energy Syst..

[24] Pati, T. K., Nayak, J. R. & Sahu, B. K. Application of TLBO algorithm to study the performance of automatic generation control of a two-area multi-units interconnected power system. In *Proceedings of the 2015 IEEE International Conference on Signal Processing, Informatics, Communication and Energy Systems (SPICES), Kozhikode, India, 19–21 February* 1–5 (2015).

[25] Dash P, Saikia LC, Sinha N (2015). Comparison of performances of several FACTS devices using Cuckoo search algorithm optimized 2DOF controllers in multi-area AGC. Int. J. Electr. Power Energy Syst..

[26] Gholipour E, Nosratabadi SM (2015). A new coordination strategy of SSSC and PSS controllers in power system using SOA algorithm based on Pareto method. Int. J. Electr. Power Energy Syst..

[27] Arya Y, Kumar N (2016). Fuzzy gain scheduling controllers for AGC of two-area interconnected electrical power systems. Elec. Power Compon. Syst..

[28] Arya Y (2021). AGC performance amelioration in multi-area interconnected thermal and thermal-hydro-gas power systems using a novel controller. Eng. Sci. Technol. Int. J..

[29] Fogel DB (1995). Evolutionary Computation Toward a New Philosophy of Machine Intelligence.

[30] Fosha CE, Elgerd OI (1970). The megawatt frequency control problem: A new approach via optimal control theory. IEEE Trans. Power Apparatus Syst..

[31] Elgerd OI, Fosha C (1970). Optimum megawatt frequency control of multi-area electric energy systems. IEEE Trans. Power Apparatus Syst..

[32] Ko HS, Leeb KY, Kim HC (2004). Intelligent based LQR controller design to power system stabilization. Electr. Power Syst. Res..

[33] Ibraheem NH, Kumar P (2013). Optimal automatic generation control of interconnected power system considering new structures of matrix Q. Electr. Power Compon. Syst..

[34] Pathak N, Nasiruddin I, Singh Bhatti T (2015). A More realistic model of centralized automatic generation control in real-time environment. Electr. Power Compon. Syst. J..

[35] Yang T, Zhang Y, Li W, Zomaya AY (2020). Decentralized networked load frequency control in interconnected power systems based on stochastic jump system theory. IEEE Trans. Smart Grid..

[36] Vlahakis E, Dritsas L, Halikias G (2019). Distributed LQR design for a class of large-scale multi-area power systems. Energies.

[37] Bimarta RV, Tran T, Kim KH (2018). Frequency-adaptive current controller design based on LQR State feedback control for a grid-connected inverter under distorted grid. Energies.

[38] Chitu C (2013). Controller design for an electric power steering system based on LQR techniques. Int. J. Comput. Math. Electr. Electron. Eng..

[39] He JB, Wang QG, Lee TH (2000). PI/PID controller tuning via LQR approach. Chem. Eng. Sci..

[40] Das S, Pan I, Halder K, Das S, Gupta A (2013). LQR based improved discrete PID controller design via optimum selection of weighting matrices using fractional order integral performance index. Appl. Math. Model..

[41] Ogata K (1994). Discrete–Time Control Systems.

[42] Naidu DS (2002). Optimal Control System.

[43] Parmar KPS, Majhi S, Kothar DP (2012). Load frequency control of a realistic power system with multi-source power generation. Electr. Power Energy Syst..

[44] Kundur PS (1994). Power System Stability and Control.

[45] Arya Y, Kumar N, Ibraheem R (2016). AGC of a two-area multi-source power system interconnected via AC/DC parallel links under restructured power environment. Optim. Control Appl. Methods..

[46] Vrdoljak K, Perić N, Petrović I (2010). Sliding mode based load-frequency control in power systems. Electr. Power Syst. Res..

[47] Zare K, Mehrdad Hagh MT, Morsali J (2015). Effective oscillation damping of an interconnected multi-source power system with automatic generation control and TCSC. Electr. Power Energy Syst..

[48] Sahu BK, Pati TK, Nayak JR, Panda S, Kar SK (2016). A novel hybrid LUS–TLBO optimized fuzzy-PID controller for load frequency control of multi-source power system. Electr. Power Energy Syst..

[49] Morsali J, Zare K, Hagh MT (2016). Performance comparison of TCSC with TCPS and SSSC controllers in AGC of realistic interconnected multi-source power system. Ain Shams Eng. J..

[50] Hakimuddin, N., Nasiruddin, I., Bhatti, T. S. & Arya, Y. Optimal automatic generation control with hydro, thermal, gas, and wind power plants in 2-area interconnected power system. *Electr. Power Compon. Syst.* **48**, 6–7, 558–571 (2020).

[51] Rakhshani E, Remo D, Mir A (2016). Analysis of derivative control based virtual inertia in multi-area high-voltage direct current interconnected power systems. Pedro Rodriguez IET Gener. Transm. Distrib..

[52] Deepak MJ, Abraham J (2015). Load following in a deregulated power system with Thyristor Controlled Series Compensator. Int J. Electr. Power Energy Syst..

[53] Mathworks. MATLAB User's Guide (R2014b) (Accessed 10 April 2022) www.mathworks.com/help/pdf_doc/matlab/index.html (2014).

[54] Markus K, Ӧgel, M. & Findeisen, R. Discrete-time robust model predictive control for continuous-time nonlinear system. In *American Control Conf, Palmer House Hilton, Chicago, IL, USA*, 1–3 (2015).

[55] Boukas E, Al-Sunni FM (2011). Mechatronic Systems Analysis, Design and Implementation.

[56] Prakash S, Sinha SK (2014). Simulation based neuro-fuzzy hybrid intelligent PI control approach in four-area load frequency control of interconnected power system. Appl. Soft Comput..

[57] Arya Y, Kumar N (2016). Fuzzy gain scheduling controllers for automatic generation control of two-area interconnected electrical power systems. Electr. Power Compon. Syst..

[58] Dasa D, Aditya SK, Kothari DP (1999). Dynamics of diesel and wind turbine generators on an isolated power system. Electr. Power Energy Syst..

[59] Sahu R, Gorripotu T, Pand S (2016). Automatic generation control of multi-area power systems with diverse energy sources using Teaching Learning Based Optimization algorithm. Eng. Sci. Int. J..

[60] Mohanty B, Panda S, Hota PK (2014). Differential evolution algorithm based automatic generation control for interconnected power systems with non-linearity. Alexandria Eng. J..

